# Identification of *DDX31* as a Potential Oncogene of Invasive Metastasis and Proliferation in PDAC

**DOI:** 10.3389/fcell.2022.762372

**Published:** 2022-02-14

**Authors:** Yongjie Xie, Yang Liu, Jinsheng Ding, Guangming Li, Bo Ni, Huifang Pang, Xin Hu, Liangliang Wu

**Affiliations:** ^1^ Department of Pancreatic Cancer, Key Laboratory of Cancer Prevention and Therapy, Tianjin Medical University Cancer Institute and Hospital, National Clinical Research Center for Cancer, Tianjin’s Clinical Research Center for Cancer, Tianjin, China; ^2^ The Graduate School, Tianjin Medical University, Tianjin, China; ^3^ Department of General Surgery, Tianjin General Surgery Institute, Tianjin Medical University General Hospital, Tianjin, China; ^4^ Department of Gastroenterology, Digestive Endoscopy Unit, Tongliao City Hospital, Tongliao, China; ^5^ Department of Epidemiology and Biostatistics, Tianjin Medical University Cancer Institute and Hospital, National Clinical Research Center for Cancer, Key Laboratory of Cancer Prevention and Therapy of Tianjin, Tianjin’s Clinical Research Center for Cancer, Key Laboratory of Molecular Cancer Epidemiology, Tianjin, China; ^6^ Key Laboratory of Cancer Prevention, Department of Gastric Cancer, National Clinical Research Center for Cancer, Tianjin’s Clinical Research Center for Cancer, Tianjin Medical University Cancer Institute and Hospital, Tianjin, China

**Keywords:** PDAC, risk model, WGCNA, DDX31, invasive metastasis, proliferation

## Abstract

**Background:** Pancreatic ductal adenocarcinoma (PDAC) is one of the deadliest malignant tumors worldwide and has poor prognosis. DEAD box proteins31 (DDX31) participate in cellular processes involving RNA secondary structure changes. However, the functions of *DDX31* in PDAC remain to be elucidated.

**Methods:** The key gene *DDX31* was identified using a combination of a risk model and weighted gene co-expression network analysis (WGCNA) with R software. The biological functions of *DDX31* in PDAC were investigated through bioinformatics analysis and *in vitro* experiments.

**Results:** Combining with WGCNA and risk model, *DDX31* was identified as a potential factor of the invasive metastasis properties of PDAC, and its expression was closely related to the malignant differentiation of PDAC. The results of gene set enrichment analysis (GSEA) showed that *DDX31* was correlated with cell invasive metastasis and proliferation by activating *MAPK* signaling pathway. The inhibition of *DDX31* inhibited the invasion and migration of PDAC cells. Survival analysis showed that *DDX31* expression was negatively associated with the poor prognosis in patients with PDAC.

**Interpretation:** *DDX31* may be a potential factor for PDAC. The inhibition of *DDX31* may be a potential way to treat PDAC.

## Research in Context

### Evidence Before This Study

Pancreatic ductal adenocarcinoma (PDAC) is one of the deadliest malignant tumors worldwide and has poor prognosis. New biomarkers can highlight biological differences among PDAC samples and help predict survival outcomes. However, new biomarkers with high sensitivity and specificity have not been discovered to date. Although various potential biomarkers of PDAC have been predicted using bioinformatics methods, they have not been identified in large samples. *DDX31*, a member of the DEAD box protein family, participates in cellular processes involving RNA secondary structure changes. However, the functions of *DDX31* in PDAC remain to be elucidated.

### Added Value of This Study

The key gene *DDX31* was identified by using a combination of a risk model and weighted gene co-expression network analysis. The biological functions of *DDX31* in PDAC were investigated through bioinformatics analysis and *in vitro* experiments. The results of gene set enrichment analysis showed that *DDX31* was correlated with cell invasive metastasis and proliferation. We found that the inhibition of *DDX31* inhibited the invasive migration of PDAC cells. Survival analysis showed that *DDX31* overexpression predicted poor prognosis in patients with PDAC.

### Implications of All Available Evidence

The findings of our research indicate that *DDX31* may be a potential prognostic biomarker for PDAC. The inhibition of *DDX31* may be a potential way to treat PDAC. Combining *DDX31* with other well-known biomarkers could be used to predict functional outcomes in patients with PDAC.

## Introduction

The 5-year survival rate of pancreatic cancer (PC) is less than 8%. It is difficult to diagnose early and treat because of its high malignancy (Balachandran et al., 2019; Klein, 2021; Neoptolemos et al., 2021). The most common type of PC is pancreatic ductal adenocarcinoma (PDAC), which accounts for >90% of exocrine PC (Fenocchio et al., 2019). Most patients with PDAC present with metastasis when diagnosed and have lost the chance to receive radical surgery treatments. However, PDAC is not sensitive to chemotherapy and radiation therapy (Anderson et al., 2021). As is well known, PDAC is a genetic disease with a large number of genetic alterations (Hayashi et al., 2021; Singhi and Wood, 2021). Thus, effective biomarkers for early diagnosis and new therapeutic targets in PC are urgently needed. Cellular proliferation and invasive metastasis are known to contribute to cancer progression, metastasis, and other malignant behaviors (Thiery et al., 2009). DDX31, a member of the Asp–Glu– Ala–Asp (DEAD) box RNA helicase (DDX) family, was first identified as an RNA helicase correlated with RNA metabolism. The critical functions of DDX family members in bacteria and archaea have been revealed in previous studies (Linder and Jankowsky, 2011). Parts of DDX family members can bind to other important nuclear proteins via the ATP-dependent pathway to form large complexes and then perform additional functions in the cytoplasm and nucleus (Jankowsky, 2011). Elevated expression of some DDX family members has been found in some types of cancer in previous studies (Abdelhaleem, 2004). Some of them play an important role in the development of cancer including promoting cancer cell proliferation, invasion, and other tumor malignant biological behaviors. Recently, DDX31 was found to perform important functions in promoting invasion and migration in muscle-invasive bladder cancer (Daizumoto et al., 2018). In recent years, extensive research has been carried out on EMT-related signaling pathways and signal targets such as transforming growth factor (TGF)/SMADs, Ras/ERK1/2, Wnt/β-catenin, aiming to find the molecular mechanism of inhibiting or reversing EMT in tumor cells, so as to develop targeted drugs for controlling tumor invasion and metastasis (Sun et al., 2015). In our study, we analyzed differentially expressed genes (DEGs) and used a combination of a risk prediction model and weighted gene co-expression network analysis (WGCNA) to identify the key functional gene, DDX31. Through comprehensive analysis and *in vitro* and *in vivo* experiments, we first reveal its clinical significance and properties in promoting tumorigenesis and invasive metastasis in PDAC.

In our study, we analyzed differentially expressed genes (DEGs) and used a combination of a risk prediction model and weighted gene co-expression network analysis (WGCNA) to identify the key functional gene, DDX31. Through comprehensive analysis and *in vitro* and *in vivo* experiments, we first reveal its clinical significance and properties in promoting tumorigenesis and invasive metastasis in PDAC.

## Materials and Methods

### Data Download and Data Preprocessing

The 3-level mRNA expression data and corresponding phenotypic data of 160 primary PC specimens were downloaded from TCGA database. Gene expression was transformed by log2 (normalized RSEM count 1). Standardized RNA data for GSE129455 and GSE62452, GSE102238 come from the GEO database. GSE129455 contains 7500 cells of four KPC mouse samples (Elyada et al., 2019), GSE62452 contains 69 PDAC samples (Yang et al., 2016), and GSE102238 contains 50 PDAC samples (Yang et al., 2020). The GSE62452 and GSE102238 datasets were used as validation sets. Quality control was implemented using the relative expression (RLE) and standardized scale-free standard error (NUSE) in the affyPLM package provided by Bioconductor (Wang et al., 2021).

### Screening of DEGs

DEGs were screened by limma (Ritchie et al., 2015) package. TCGA and GEO datasets contained DEGs of metastatic and non-metastatic patients as well as cancer and normal tissues. The results were displayed by volcano plot, which were drawn using ggplot2 (Ito and Murphy, 2013).

### Functional Enrichment Analysis

The clusterProfile (Yu et al., 2012) package was used to perform Gene Ontology (GO) enrichment analysis, including biological processes (BPs), molecular functions (MFs), and cellular components (CCs), and Kyoto Encyclopedia of Genes and Genomes (KEGG) (Kanehisa and Goto, 2000) enrichment analysis. An adjusted p value <0.05 was considered statistically significant. GSEA local software was used for enrichment analysis and visualization. The threshold of gene set enrichment analysis (GSEA) (Subramanian et al., 2005) was set to adjusted p value <0.05 and FDR <0.25 after correction. “c2. cp. kegg. v7.0. symbols. gmt” was selected as in review the reference gene set.

Lasso-Cox regression analysis was conducted using glmnet (Friedman et al., 2010) package. The expression of the selected genes was combined with multivariate Cox regression coefficient to establish a prognostic model. The risk score of each patient is equal to the sum of gene expression and regression coefficient. The median risk score was used as the threshold to evaluate the model’s prediction effect to stratified PC patients. Patients with a risk score higher than the threshold were assigned to the high-risk group, and the rest were assigned to the low-risk group. Log-rank test was used to evaluate the difference of survival rate between the two groups. In addition, on the basis of the increase in risk value, the distribution of patients’ death events is shown by the point diagram. The heat map was used to observe the expression distribution of each characteristic gene in two different risk groups. The time-dependent receiver operating characteristic (ROC) curve was used to evaluate the risk score, and the specificity and sensitivity of predicting the survival rate of PC in 1-, 3-, and 5-year follow-up were observed. TNM staging (III–IV/I–II) and histological grade (4/3/2/1) were transformed into classification variables. The clinical features and risk scores of PDAC patients in TCGA cohort were analyzed by univariate and multivariate Cox regression analysis. The clinical features and risk scores were used to establish a nomogram model. The calibration analysis (Guy et al., 2019) was performed aligned with nomogram. Decision curve analysis (DCA) (Van Calster et al., 2018) was conducted to verify the risk model.

### Weighted Gene Co-Expression Network Analysis

WGCNA (Guy et al., 2019) was conducted using the WGCNA package. It is a systematic way for effectively acquiring the expression patterns of multiple genes in different samples, which can obtain a gene cluster with the same expression pattern.

### Human Tissue Specimens and Immunohistochemical Analysis

We were allowed by the Ethics Committee of Tianjin Cancer Institute and Hospital (Tianjin, China) to acquire the paraffin sections of four patients. A total of 86 patients received radical pancreaticoduodenectomy from January 2017 to September 2020. We randomly selected clinical patients who had been diagnosed as TNM3, TNM2, and TNM1. IHC of DDX31 was performed on tumor tissues of patients and matched normal pancreas tissues. Fourteen cases of fresh clinical tumor and normal pancreas tissue samples were collected to identify the expression of DDX31 by Western blot analysis. IHC analysis of the PDAC tissue for DDX31 (NOVUS; NBP1-21322, 1:400) was performed using a DAB substrate kit (ORIGENE, ZLI-9019). Three representative images of PC tissues IHC stain (100×, 200× magnification) and representative paired normal and tumor tissues IHC stain (100×, 200× magnification) were evaluated under a light microscope.

### Cell Culture and Reagents

Human PC cell lines (BxPC-3, MIA-PaCa2, SW 1990, and L3.7) were purchased from the ATCC (Rockville, MD). All PC cell lines were cultured in a 5% CO2 incubator at 37°C. PC cell lines were cultured in RPMI-1640 medium and DMEM (GIBCO) with 10% fetal bovine serum (FBS).

### Plasmid Construction and Cell Transfection

DDX31 overexpression in PC cell lines and lentivirus-mediated plasmid was conducted using the pCDH-cDNA system (Biosettia) following the manufacturer’s instructions. Lentiviruses were produced in 293T cells for the stable transfection of cell lines. Human DDX31 cDNA was cloned into a pCDH plasmid expression vector (pCDH-DDX31), and the pCDH vector was used as the control. Stable cell lines were generated using puromycin. The overexpression efficiency was confirmed by Western blot analysis. Stable knockdown PC cell lines and shRNA were designed using http://biosettia.com/support/shrna-designer. PLVi-shRNA-bsd vectors were purchased from Biasatti. Three shRNA sequences for DDX31 were synthesized and cloned into the plasmid. Detailed information of the shRNA sequence for DDX31 is listed in Supplementary Table S1. The most effective one was used for the next experiments. The most effective shRNA was identified by Western blot analysis.

### Animal Studies in the Subcutaneous PC Mouse Model

Five-week-old female nude NU/NU mice were purchased from SiPeiFu Biotechnology Co. All mice were maintained in a barrier facility on HEPA-filtered racks. All animal studies were conducted under an approved protocol (Zhao et al., 2020). Tumor cells were harvested by trypsinization, washed with ice PBS, and resuspended at 1 × 10^7^ cells per milliliter in PBS. Subsequently, 1 × 10^6^ cells were used to establish every subcutaneous xenotransplant tumor model of human PC in nude mice. In the log phase, BxPC-3 was implanted subcutaneously in nude mice and observed three times a week.

### Statistical Analysis

Statistical analysis was performed with GraphPad Prism version 8.0 (San Diego, CA, United States), R software 3.4.0.3, and SPSS version 26.0 (IBM SPSS, Armonk, NY, United States). Lasso-Cox regression analysis was performed using the R software package glmnet. In addition, the survdiff (Tan et al., 2020) function in the survival package was used for the logarithmic rank test. Time-dependent ROC was analyzed by timeROC (Blanche et al., 2013) package. Volcano plot was drawn with ggplot2 package. The establishment and application of line graph were realized with package rms (Sheng Zhang et al., 2019). The enrichment analysis was conducted with the R package cluster filer. Each experiment was conducted in triplicate, and data were presented as the mean ± SD unless otherwise stated. The variance between groups was statistically compared. Student’s t-test was conducted to compare mean values. Correlations between DDX31 expression level and patients’ survival time after surgery were determined by Kaplan–Meier method. The categorical data were analyzed by Chi-square test. *p < 0.05, **p < 0.01, ***p < 0.001, and ****p < 0.0001 indicated significant differences, and NS meant nonsignificant.

## Results

### Identification of DEGs and Functional Enrichment Analysis

**FIGURE 1:**
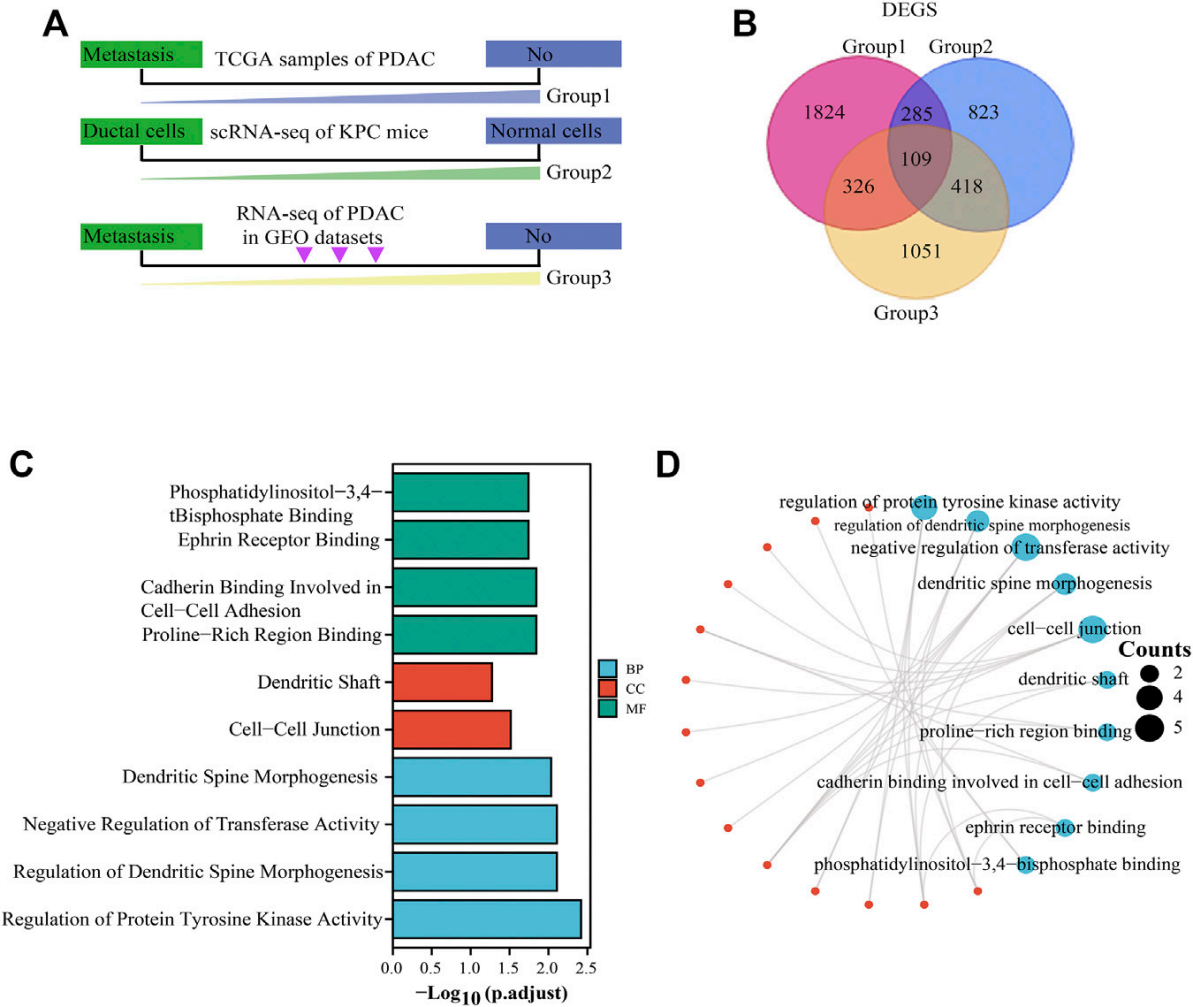
DEG screening and functional enrichment analysis. **(A)**: Workflow of DEG screening. Group 1 represents TCGA sample screening (divided into “with metastasis” and “without metastasis” group), group 2 represents KPC mouse single-cell transcriptome sequencing (divided into “ductal cells” and “normal cells” group), and group 3 represents GEO data sample (divided into “with metastasis” and “without metastasis” group). **(B)**: Concomitant upregulated DEGs in group 1, group 2, and group 3. Red indicates group 1, blue indicates group 2, and yellow indicates group 3. **(C)**: GO analysis of upregulated DEGs. Green represents BP, red represents CC, and blue represents MF. Adjusted *p* < .05. **(D)**: Network diagram of GO analysis of upregulated DEGs. The red dots represent the interacting genes, and the blue dots represent the ID of the pathway, and the number of genes is represented by the size of the circle. DEGs: differentially expressed genes.

Workflow of identification of DEGs among the sequencing data of TCGA, KPC-scRNAseq, and GSE36668 in PC was shown in Figure 1A. The concrete work flowchart was presented in Supplementary Figure S1. TCGA and GSE36668 datasets were divided into “with metastasis” and “without metastasis” group; KPC mouse single-cell transcriptome sequencing datasets were divided into “ductal cells” and “normal cells” group; The flowchart showed our study aimed to screen the differentially expressed genes based on screening conditions that are highly expressed and prone to metastasis in cancer. The intersection of upregulated DEGs among the sequencing data of TCGA, KPC-scRNAseq, and GSE36668 in PC was composed of 109 genes (Figure 1B), and the DEGs are shown in the volcano plot in Supplementary Figures S2A–C. The unsupervised clustering viable cells from KPC-scRNAseq were presented in Supplementary Figures S2D,E. All the differentially expressed genes were showed in Supplementary Table S1, and the results indicated that 104 genes such as ADAMTS2, BIRC5, MYC, ID1, VIM, CDH2, SMAD7, AXIN2, FAP, CAP1, SNAI1, CD59, ANXA8, YAP1, DUSP4, SOX2, KRT9, KRT13 et al. were enriched in “metastasis group” and had high expression in pancreatic ductal adenocarcinoma. Part of these genes have been reported in basic studies of pancreatic cancer and are closely related to the invasion and metastasis of pancreatic cancer according to the literature, including MYC (Sodir et al., 2020), CDH2 (Shintani et al., 2008), SNAI1 (Dai et al., 2017), YAP1, SOX2 (Zhang et al., 2017). Besides, these genes, ADAMTS2 (Dey et al., 2020), KRT9 (Andolino et al., 2018), KRT13 (Nguyen et al., 2021), LY6D (Barros-Silva et al., 2018), had been reported in a variety of cancers. GO enrichment analysis (Table 1) was performed on the intersections. The results showed that the upregulated DEGs were mainly enriched in BPs, such as Cadherin Binding involved in cell-cell Adhesion, Ephrin Receptor Binding, and Proline-Rich Region Binding, and CCs, such as tight junctions, Dendritic Shaft, and Cell-Cell Junction. Moreover, they mainly play the molecular function (MF) of Negative Regulation of transferase Activity and Regulation of Protein Tyrosine Kinase Activity (Figures 1C,D).

**TABLE 1:** Relationships of *DDX31* expression and clinicopathological characteristics in 86 patients with PC.

Feature	All	*DDX31* expression		χ^2^	*p*
		Low	High		
	86	*n* = 47	*n* = 39		
Age(year)				0.28901	0.5909
<60	48	25	23		
≥60	38	22	16		
Gender				0.53181	0.4658
Male	56	29	27		
Female	30	18	12		
pTNM stage				5.8091	0.0159
I	19	15	4		
II–III	67	32	35		
Histological grade				4.3641	0.0369
G1	20	15	5		
G2/G3	66	32	34		
Tumor diameter				3.9681	0.0464
<5	41	27	14		
≥5	45	20	25		
Lymph node metastasis				9.9801	0.0016
No	49	34	15		
Yes	37	13	24		
Vascular invasion				3.2031	0.0735
No	69	41	28		
Yes	17	6	11		
Nerve invasion				1.1831	0.2768
No	80	45	35		
Yes	6	2	4		

### Lasso-Cox Analysis Screening Key Target Genes

**FIGURE 2:**
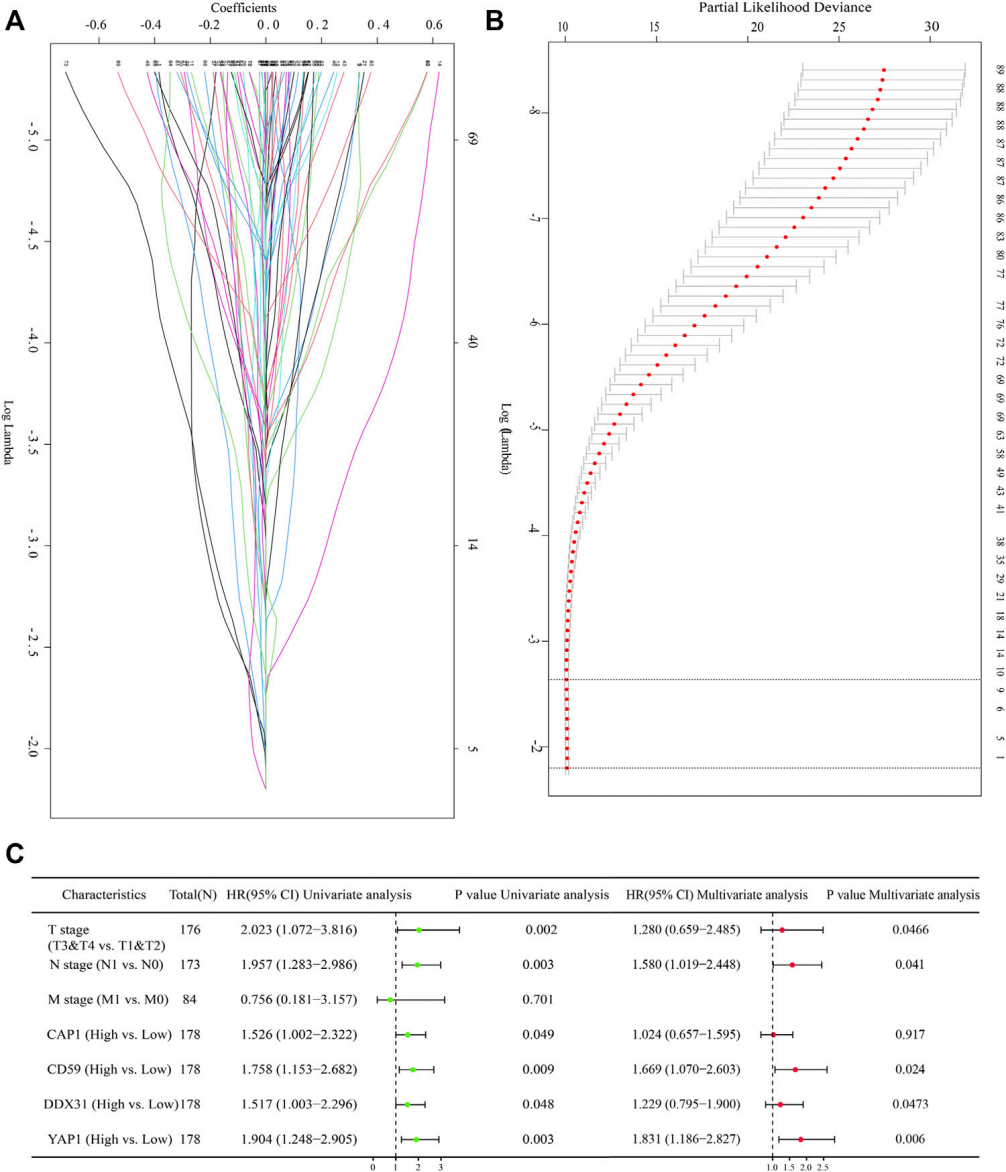
Screening of prognostic genes with Lasso regression analysis. **(A)**: Lasso-Cox regression confirmed the regression coefficients of each gene. Lasso method can achieve the effect of variable selection, the insignificant variable coefficient compressed to 0; With the increase of lambda, its absolute value is compressed accordingly, and some relatively unimportant variables are compressed to 0. The ordinate represents the regression coefficient, The horizontal coordinate represents the log λ value; **(B)**: Identifying the suitable parameter (λ). Fit curve is a curve that matches the lasso regression to show the appropriate log λ value. The log λ value of the range of the two straight lines in the middle perpendicular to the X axis is the optimal log λ value range. **(C)**: Forest diagram showing the selected variables with univariate and multivariate cox regression analysis. The selected variables contained *DDX31*, *CAP1*, *CD59*, *YAP1*; The common clinical staging parameters such as T, N, M staging were included in multivariate Cox regression analysis. P value less than .05 is considered to be statistically significant, and HR > 1 has the significance of promoting risk; HR < 1 has the significance of inhibiting risk.

Lasso is another data dimensionality reduction method, which is not only suitable for linear cases, but also suitable for nonlinear cases. Lasso selects the variables of sample data based on the penalty method. By compressing the original coefficients, the originally small coefficients are directly compressed to 0, so that the variables corresponding to these coefficients are regarded as non significant variables and the non-significant variables are directly discarded. In our study, Lasso-Cox regression analysis was conducted on the selected DEGs according to the coefficients and log(λ) value. Four variables (including *CAP1*, *CD59*, *YAP1*, and *DDX31*) were screened through the change of regression parameters and the final regression to zero variables (Figures 2A,B). Lasso regression showed that the four variables contributed significantly to the penalty coefficient of pancreatic cancer patients’ outcomes, which could be used as candidate variables for further analysis. In order to further determine whether these four variables affect the survival of patients with pancreatic cancer, univariate cox regression analysis and multivariate cox regression analysis were conducted on these four variables and clinical factors (Figure 2C). These clinical factors contained T stage (T3&T4 vs T1&T2), N stage (N1 vs N0) and M stage (M1 vs M0), which were often used to reflect important clinical risk level for tumor progression. The results were summarily displayed in the forest plot, including *CAP1* (HR = 1.526, *p* = 0.049), *CD59* (HR = 1.758, *p* = 0.009), *DDX31* (HR = 1.517, *p* = 0.048), *YAP1* (HR = 1.904, *p* = 0.003), T stage (HR = 2.023, *p* = 0.002), N stage (HR = 1.957, *p* = 0.003), and M stage (HR = 0.756, *p* = 0.701) in univariate analysis and *YAP1* (HR = 1.831, *p* = 0.005), *CD59* (HR = 1.669, *p* = 0.024), *DDX31* (HR = 1.229, *p* = 0.0453) T stage (HR = 1.280, *p* = 0.0466), and N stage (HR = 1.580, *p* = 0.041) in multivariate analysis (Figure 2C). The T, N, M stage can reflect the ability of tumor invasion and metastasis, progression and survival of patients. It is commonly used to assess the survival status of cancer patients. Thus, we found that these four parameters combined with T, N, M staging may be a pathogenic factor in pancreatic cancer. Therefore, we used these four variables to jointly construct a prediction model to assess the invasion and metastasis and survival status of patients with pancreatic cancer.

We still found in the analysis results that a small number of patients with high expression of DDX31 had HR less than 1 (DDX31′s HR value partially crossed 1), but the analysis results of most patients showed that it might be a risk factor of pancreatic cancer patients. But we still need to explore the reasons for its low risk in subsequent experiments.

### Construction of the Risk Prediction Model With *CAP1*, *CD59*, *YAP1* and *DDX31*

**FIGURE 3:**
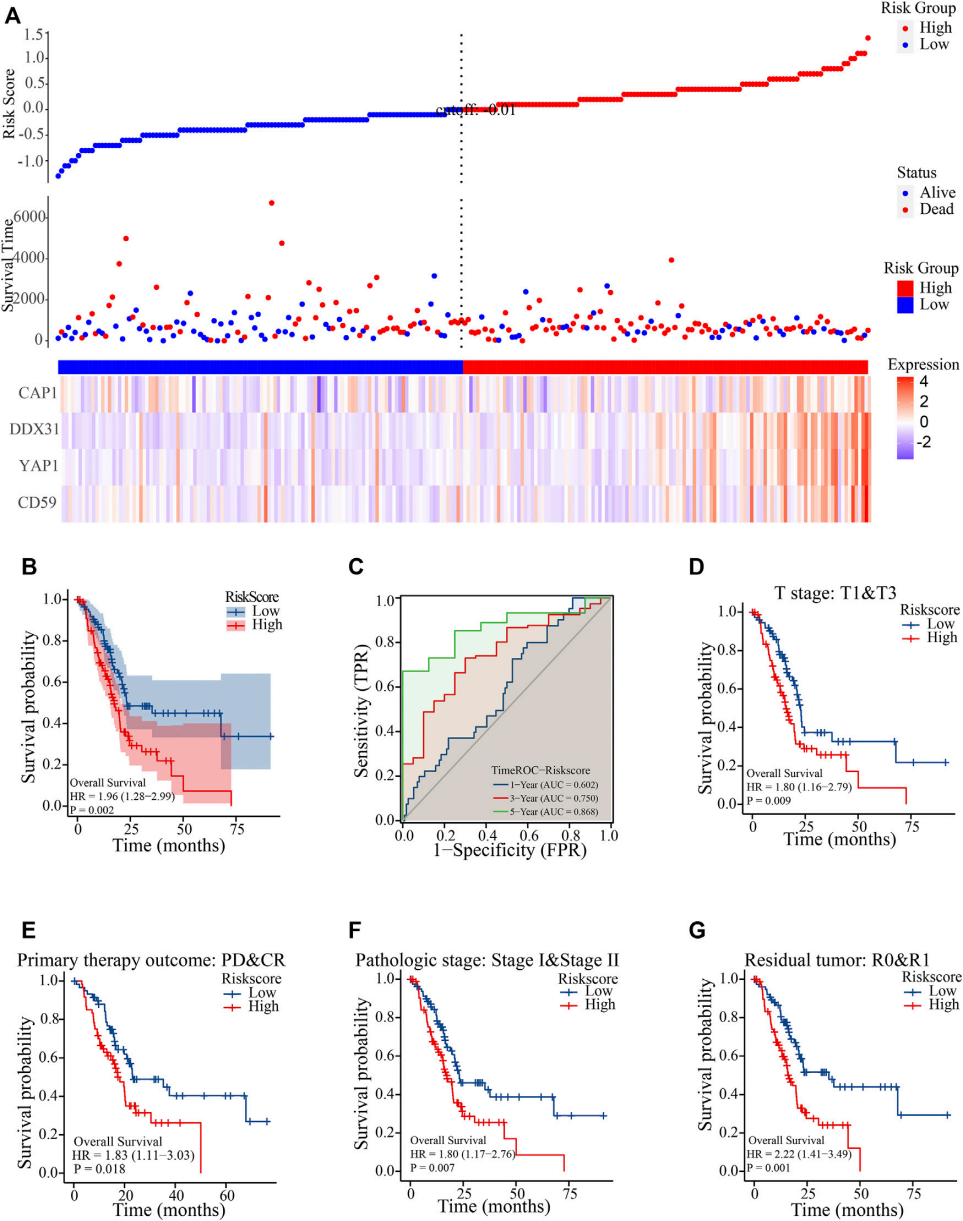
Construction of the risk model. **(A)**: Constructing the risk prediction model with prognostic genes. Red represents the high-risk group, and blue represents the low-risk group. The risk model diagram contains three parts: risk value, patient’s survival state and expression level of risk factors. The expression heat map of risk factors is below, and the risk score curve is above. **(B)**: Survival K–M curve analysis of high- and low-risk groups. Red represents high-risk group, and blue represents low-risk group. *p* value less than 0.05 is considered to be statistically significant, and HR > 1 has the significance of promoting risk; HR < 1 has the significance of inhibiting risk. **(C)**: Time-dependent ROC curve of risk scores in prediction analysis of 1-, 3-, and 5-year survival time. The ordinate represents the sensitivity, The horizontal coordinate represents the 1-specitivity; the blue curve indicates 1-survival time, the red curve indicates 3-survival time, the green curve indicates 5-survival time; **(D–G)**: Survival K–M analysis of patients in high- and low-risk groups in subgroups of clinical stages **(D)**, pathological grade **(E)**, tumor recurrence **(F)**, and primary treatment prognosis **(G)**. P value less than .05 is considered to be statistically significant, and HR > 1 has the significance of promoting risk; HR < 1 has the significance of inhibiting risk. the blue curve indicates low-risk score, the red curve indicates high-risk score.

A metastasis-related prognostic model was constructed on the basis of the four selected prognostic genes (*CAP1*, *CD59*, *YAP1*, *DDX31*). In our model, the probability of survival of patients was positively correlated with the risk score accompanying with the expression of *CAP1*, *CD59*, *YAP1*, and *DDX31* (Figure 3A). the mRNA expression of CAP1, CD59, YAP1 and DDX31 was elevated obviously in high risk groups, and ultimately the high-risk group had more deaths than low-risk groups. A total of 178 PDAC samples in TCGA cohort study were used to evaluate the risk model. The results showed that the survival rate of the high-risk group was significantly poorer than that of the low-risk group (*p* < 0.0001), and the median survival time was significantly shortened (Figure 3B). In clinical epidemiological research, disease status and markers change over time (time-to-event outcomes). The early disease-free individuals may have a late onset due to the long follow-up time, and their markers may change from baseline during the follow-up. If the traditional ROC is used, the disease status or the time dependence of markers will be ignored. At this time, the time-dependent ROC is more appropriate. So, we performed a time-dependent ROC diagnosis and prediction analysis. We found that the 1-year survival rate prediction rate (0.602), 3-year survival rate prediction rate (0.745), and 5-year survival rate prediction rate (0.819) of the model was reliable, which were more than 0.55, further proving the effectiveness of the model (Figure 3C). In order to further evaluate the prediction performance of the risk model, we selected different clinical parameters as subgroups between high- and low-risk groups to verify the model. The median overall survival (OS) of the high-risk group was significantly shorter than that of the low-risk group in clinical stage (stage I and stage II, HR = 1.80, *p* = 0.007, Figure 3D), pathological stage (stage I and stage II, HR = 1.62, *p* = 0.026, Figure 3E), residual tumor recurrence (R0 and R1, HR = 2.22, *p* = 0.001, Figure 3F), and primary tumor treatment efficacy (PD and CR, HR = 1.83, *p* = 0.018, Figure 3G). In our model, the higher the risk score, the earlier the patient event occurred. The above results showed that we had established an effective metastasis-related model in PC. To exclude the possibility of overfitting of the model in TCGA, the risk model was verified in the GEO datasets. We analyzed the survival rate in the risk model between the high- and low-risk groups in the GEO datasets. The results showed that the probability of survival in the high-risk group was poor (HR = 2.04, *p* = 0.019), and the median survival time in the high-risk group was significantly shorter than that in the low-risk group (Supplementary Figure S3A). We also performed a time-dependent ROC diagnostic prediction analysis (Supplementary Figure S3B) and found that the 1-year survival rate prediction (0.661), 3-year survival rate prediction (0.703), and 5-year survival rate prediction (0.779) all had valuable predictive effects, which were greater than 0.55, indicating the effectiveness of the model. In addition, we carried out DCA (Supplementary Figure S3C) and found that the risk score has good decision-making effect. The nomogram has been regarded as a reasonable tool to create a easy intuitive graph for a statistical predictive model that quantifies the risk of a clinical event. The nomogram prediction model was also constructed (Supplementary Figure S3D) on the basis of risk factors and clinical characteristics. The 1-, 3-, and 5-year survival rate prediction of patients can match well. Furthermore, the prediction effect of the risk score was higher than that of clinical parameters, reflecting the prognostic value of the risk model. Calibration analysis was also conducted with risk scores. The results showed that the calibration of 1-, 3-, and 5-year survival corresponded to the expected prediction effect (Supplementary Figure S3E). The above results indicate that the risk model can be applied on different platforms. The percentage of copy number variation (*CAP1*, 4%; *CD59*, 4%; *DDX31*, 4%; *YAP1*, 1.8%) was calculated in TCGA samples (Supplementary Figures S4A,C). In addition, we further analyzed the clinical pathological factors and risk scores (*p* < .05, HR > 1) by univariate and multivariate Cox regression analysis (Supplementary Figures S3F,G), which further showed the prognostic value of the risk model.

### Function of *DDX31*

**FIGURE 4:**
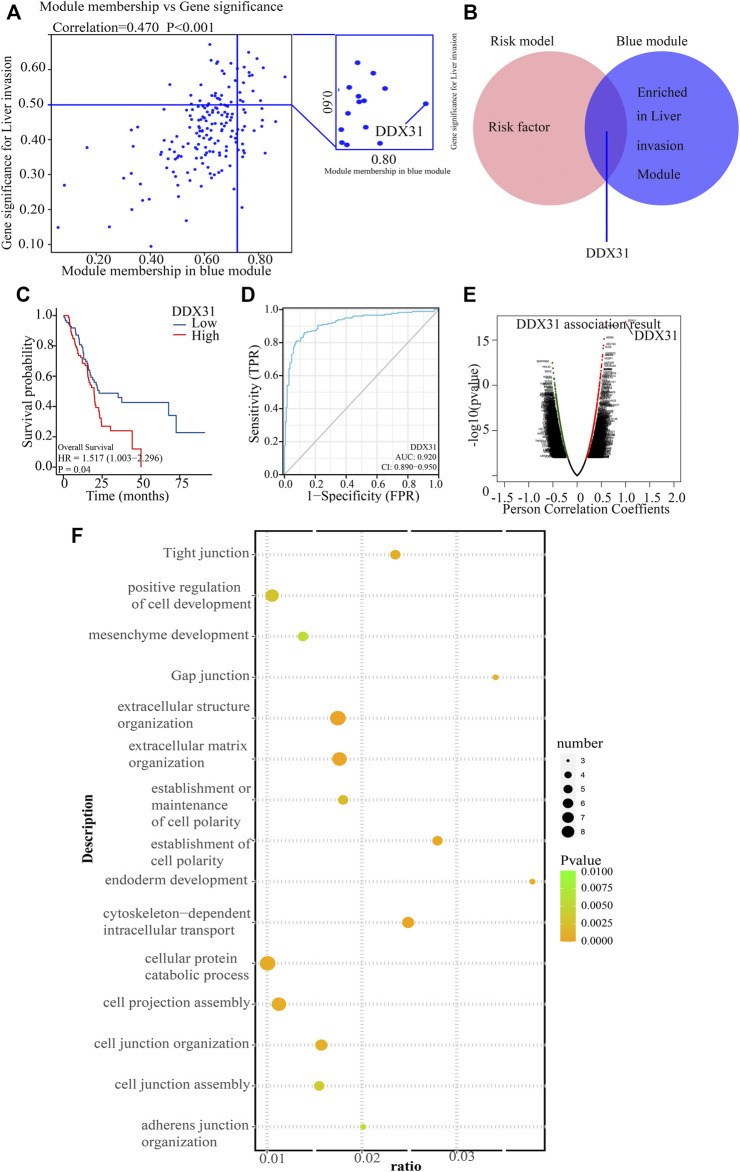
Functional analysis of target gene *DDX31*. **(A)**: The blue module containing *DDX31* was correlated with PDAC liver metastasis. A scatter plot was mapped between GS and MM (blue module and liver metastasis) in PDAC patients. **(B)**: The intersected gene, *DDX31*, was identified between risk factors in the risk model, and genes contained in blue modules with Venn diagram. **(C)**: Difference in survival K–M curve of *DDX31* in PC. Red represents the high-expression group; blue represents the low-expression group. P value less than .05 is considered to be statistically significant, and HR > 1 has the significance of promoting risk; HR < 1 has the significance of inhibiting risk. **(D)**: ROC prediction analysis of *DDX31* in PC. AUC value is on the lower right. **(E)**: Volcano plot of *DDX31*-positive related genes. **(F)**: GO enrichment analysis of positively correlated genes with *DDX31*. **(G)**: KEGG enrichment analysis of positively correlated genes with *DDX31*.

WGCNA is a integrated way for effectively acquiring the expression patterns of multiple genes in different samples, which can obtain a gene group with the same expression pattern. The association between modules and phenotype of samples such as clinical characteristics can be studied. A total of 170 samples with clinical characteristics were included in WGCNA (Supplementary Figure S5A). In this study, the power of β = 9 (scale-free R2 = 0.8Figure 50) was selected as the soft threshold to establish the scale-free network (Supplementary Figures S5B,C). Therefore, three co-expressed modules were identified after removing the gray modules by combined dynamic tree cutting (Supplementary Figure S5D). The TOM was mapped to 2 Figures, 544 genes selected in the analysis, indicating that each module was independently verified (Supplementary Figure S5F). We found that the blue module containing *DDX31* was correlated with PDAC liver metastasis (R2 = 0.38, *p* = 0.04). A scatter plot was mapped between GS and MM (blue module and liver metastasis) in PDAC patients (correlation = 0.470, *p* = 0.70). Therefore, *DDX31* was considered to be one of the, critical genes in the blue module (Figure 4A). *DDX31* was identified as the common target gene between the risk model and weighted co-expressed network through Venn diagram. Then, we determined *DDX31* is a prognostic target gene (Figure 4B). We compared the probability of survival between patients with high *DDX31* expression and patients with low *DDX31* expression. The results showed that the OS of patients with high *DDX31* expression was significantly decreased, and the median survival time was significantly shortened (Figure 4C). ROC diagnostic prediction analysis (Figure 4D) was conducted, and the AUC was 0.920, indicating that *DDX31* had a good predictive effect on PC metastasis. Then, 135 significant genes that were positively correlated with *DDX31* were selected and shown in a volcano plot (Figure 4E). Then, we conducted GO analysis on these genes and found that they were mainly enriched in ribosome biogenesis, nuclear export, and rRNA metabolic process. These genes mainly play a critical role in the production of nuclear material components, signal transmission, and structural shape (Figure 4F). In addition, KEGG pathway enrichment analysis was performed, and the results showed that gap junction, extracellular structure organization, cell junction assembly, invasion and metastasis, and cell adhesion were significantly enriched (Figure 4G).

### *DDX31* Might Be a Potential Oncogene in PDAC

**FIGURE 5:**
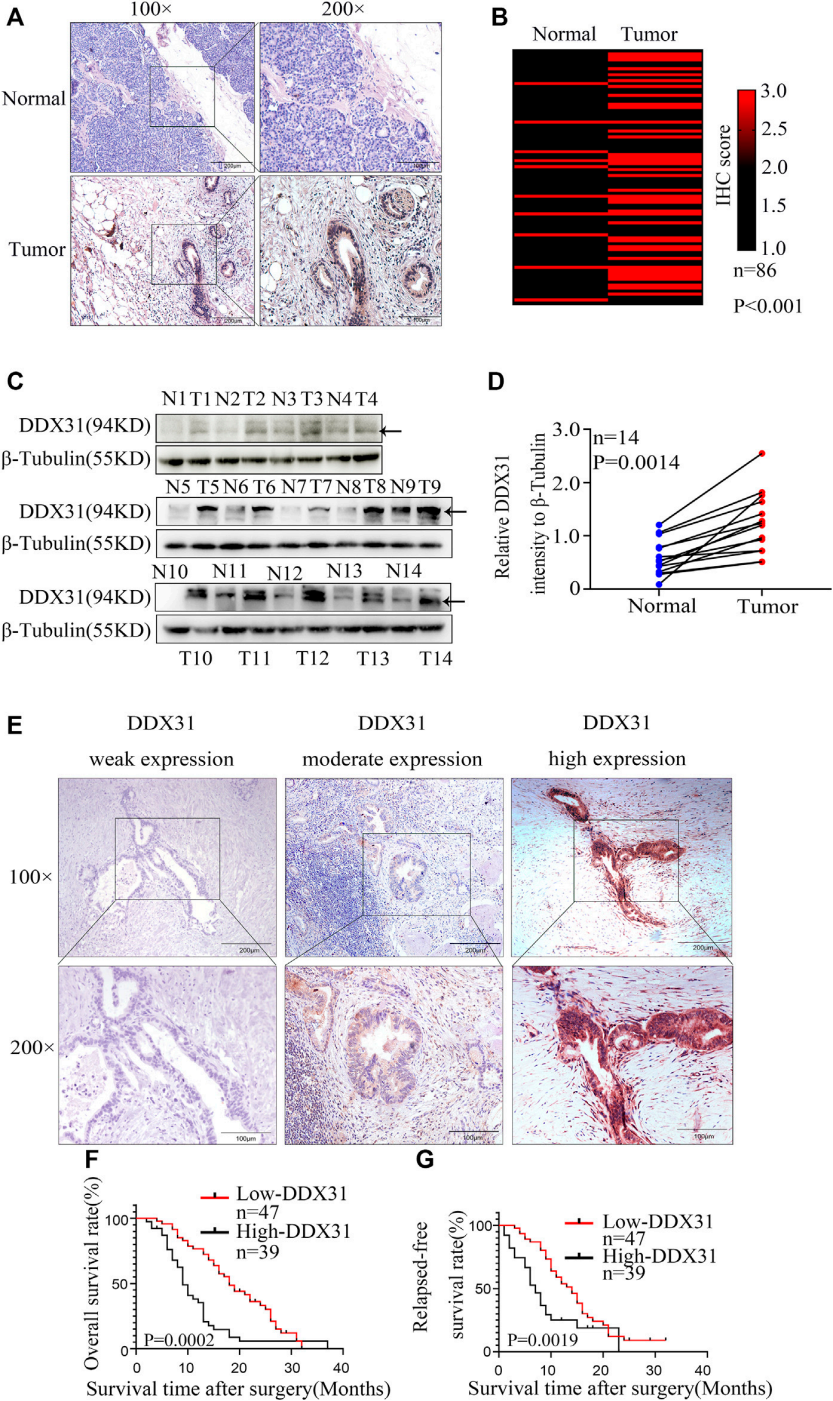
Expression of *DDX31* in PDAC tissue and its clinical significance. **(A)**: IHC analysis of *DDX31* in PDAC (×100 and ×200 magnification, bar = 200 and 100 μm). **(B)**: The differential expression of *DDX31* in 86 normal pancreas tissues and paired tumor tissues is shown in a heat map and was statistically analyzed by Wilcoxon signed rank tests. **(C)**: Fourteen paired fresh tumor tissues and normal tissues were attained, and Western blot analysis was performed to identify the elevated expression in PDAC tissues compared with normal tissues. **(D)**: Statistical result of relative *DDX31* expression to β-tubulin Western blot is shown in Figure 7D. **(E)**: IHC stain of *DDX31* in PDAC tissue paraffin section. Representative images for weak, moderate, and high expression of *DDX31* in PDAC tissues are shown (×100 and ×200 magnification, bar = 200 and 100 μm). **(F,G)**: Difference analysis of overall survival (OS, L) rate and relapse-free survival (RFS, R) rate between *DDX31* low- (weak and moderate expression) and high-expression group. *p < .05.

To our knowledge, no study has investigated the biological function of *DDX31* in PDAC. To investigate the expression of *DDX31* in PDAC specimens, we performed IHC analysis in 86 tumor tissues and paired normal pancreas tissues (Figures 5A,B). According to the results of IHC, 62.8% (54/86) of patients, the expression of DDX31 was higher in tumor tissues than in paired normal tissues. Interestingly, in this series of 86 patients, 16.7% (9/86) of patients, the expression of DDX31 was lower in tumor tissues than in paired normal tissues and the expression was the approximate level in normal and tumor tissues in 26.7% of (23/86) these patients. According to the results of IHC, we speculate that the reasons may be as follows : First, it may be the existence of heterogeneity among pancreatic cancer patients in our center ; furthermore, there may be some possible copy number variations in this part of the patients resulting in a decline in their expression levels. Moreover, of these tissues, 14 paired fresh tumor tissues and normal pancreatic tissues were selected randomly to perform Western blot analysis (Figures 5C,D). We found that the expression of *DDX31* was increased in tumor tissues. The 86 tumor tissues were divided into two groups according to the expression of *DDX31* (*DDX31*-Low and *DDX31*-High) (Figure 5E). According to the results of correlation analysis of *DDX31* expression and patients’ clinical pathological features, we found that high *DDX31* expression was strongly correlated with the tumor size (χ^2^ = 3.9681, *p* = 0.0464), lymph node metastasis (χ^2^ = 9.9801, *p* = 0.0016), and TNM grade (χ^2^ = 5.8091, *p* = 0.0159) of PDAC patients and histological grade of tumor tissues (χ^2^ = 4.3641, *p* = 0.0369) (Table 1). At the same time, the Kaplan–Meier analysis of TMA data showed that patients with high *DDX31* expression had significantly lower OS rate and relapsed-free survival (RFS) rate compared with patients with low *DDX31* expression (Figures 5F,G). Next, univariate and multivariate analyses of clinical follow-up data of PDAC patients were performed. The results indicated that the expression of *DDX31* was negatively correlated with OS and RFS in PDAC patients (Table 2). Together, our results indicated that high expression of *DDX31* might be a risk factor of poor prognosis in PDAC occurrence and progression.

**TABLE 2:** Univariate and multivariate analysis of clinicopathological factors for overall survival rate (OS) and relapsed-free survival rate (RFS).

Features	Univariate analysis					
	OS			RFS		
	HR (95%CI for HR)		*p*	HR (95%CI for HR)		*p*
Sex			0.531			0.629
Male	1			1		
Female	1.167 (0.720–1.892)			0.876 (0.511–1.500)		
Age			0.744			0.86
<60	1			1		
≥60	1.080 (0.681–1.711)			0.956 (0.583–1.570)		
Histological grade			0.02			0.018
G1	1			1		
G2/G3	2.626 (1.443–4.778)			2.042 (1.132–3.684)		
Tumor diameter			0.026			0.15
<5 cm	1			1		
≥5 cm	1.707 (1.066–2.734)			1.447 (0.875–2.394)		
Lymph node metastasis			<0.001			0.002
Negative	1			1		
Positive	2.722 (1.642–4.513)			2.266 (1.343–3.825)		
Vascular invasion			<0.001			<0.001
Negative	1			1		
Positive	3.502 (1.958–6.263)			3.164 (1.713–5.842)		
Nerve invasion			0.022			
Negative	1			1		0.199
Positive	2.768 (1.155–6.633)			1.974 (0.700–5.570)		
TNM stage			<0.001			<0.001
I	1			1		
II	3.844 (1.723–8.573)		0.001			
III	14.390 (6.130–33.783)		<0.001	3.275 (1.472–7.187)		<0.001
*DDX31* expression level			<0.0001	9.172 (3.869–21.744)		<0.001
Low expression	1			1		
High expression	2.337 (1.450–3.766)			2.165 (1.293–3.624)		
**Features**	**Multivariate analysis**					
Vascular invasion			0.038			0.019
Negative	1			1		
Positive	2.032 (1.040–3.967)			2.324 (1.148–4.707)		
TNM stage			<0.001			<0.001
I	1			1		
II	4.129 (1.810–9.421)		0.001	3.308 (1.458–7.501)		
III	12.196 (4.964–29.964)		<0.001	7.038 (2.835–17.470)		0.004
*DDX31* expression level			0.022			0.037
Weekly expression	1			1		
High expression	1.840 (1.090–2.987)			1.764 (1.036–3.003)		

### *DDX31* Promoted PDAC Cellular Migration

**FIGURE 6:**
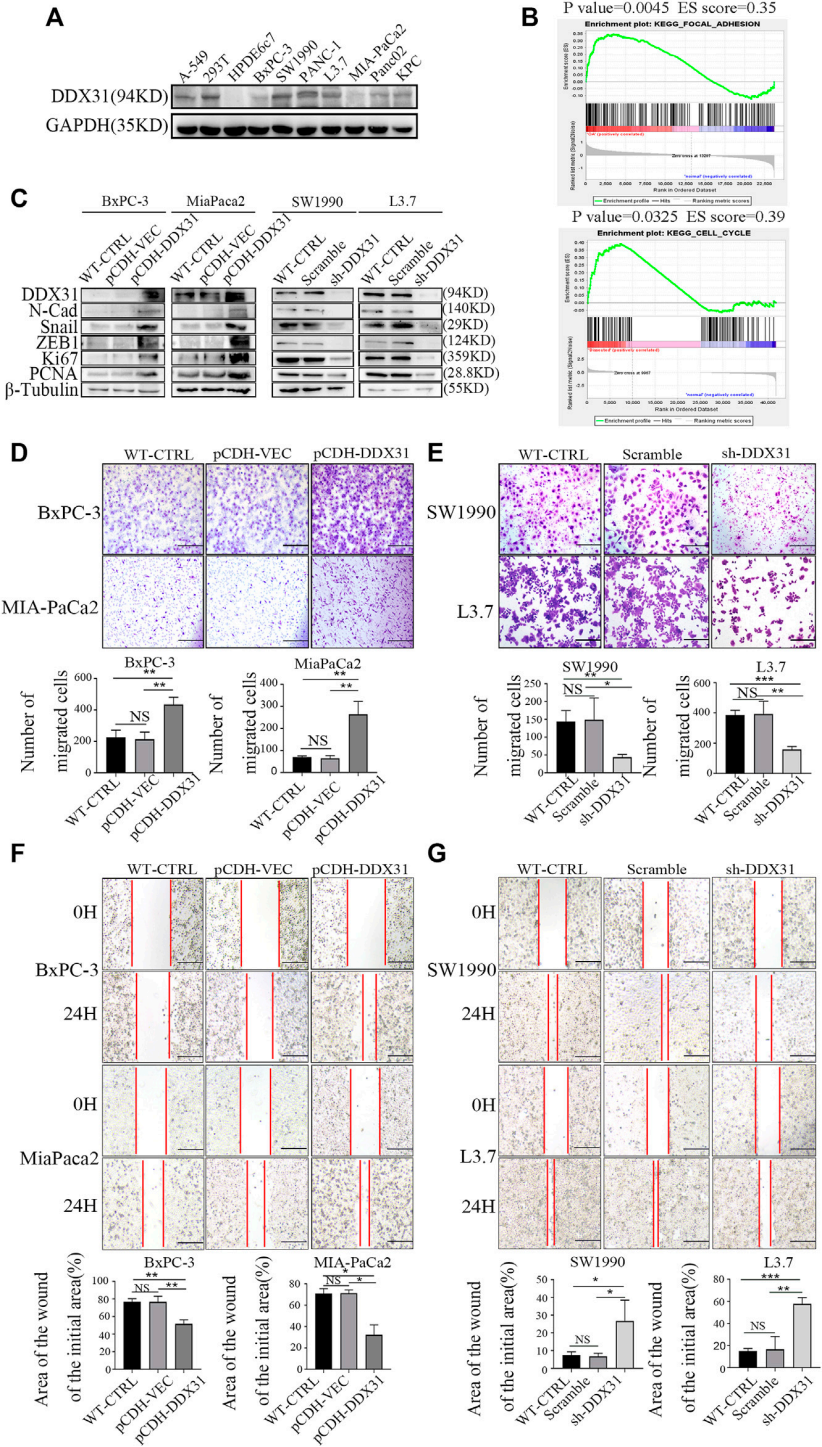
*DDX31* promoted PDAC cell migration. **(A)**: The basic protein expression of *DDX31* in all PDAC cell lines was measured with Western blot analysis. **(B)**: GSEA was carried out according to the expression level of *DDX31*; p value < .05, FDR < 0.25. **(C)**: Loss and gain-of-function studies according to the results of Western blot analysis were performed (*DDX31* overexpression on BxPC-3/MIA-PaCa2 cell lines, *DDX31* knockdown on SW1990/L3.7 cell lines). **(D,E)**: Cell migration was detected by transwell assays (bar = 200 μm) and wound healing experiments (bar = 200 μm) in *DDX31*-overexpression PDAC cell lines BxPC-3 and MIA-PaCa2. **(F,G)**: Transwell assay and wound healing assay were performed when *DDX31* was downregulated in PDAC cells (SW1990 and L3.7). Images were collected three times randomly from each experiment (overexpression, left; knockdown, right). The Figure ure at the bottom shows the corresponding statistical chart (unpaired t-tests). Values are presented as the means ± SDs of three independent experiments. *p < 0.05, **p < .01, ***p < .001, ****p < .0001, and NS means non significant.

To explore the potential roles of *DDX31* in PDAC cells, the basic protein expression of *DDX31* in all cell lines was determined (Figure 6A). We found that most PDAC cell lines showed higher protein expression of *DDX31* than the immortalized normal ductal epithelial cell line HPDE6c7. MIA-PaCa2 with low endogenous expression of *DDX31* and SW1990 with high endogenous expression of *DDX31* were chosen to confirm the cellular location of *DDX31* in PDAC cells (Supplementary Figure S5A). The results of immunofluorescence analysis revealed that *DDX31* was mainly localized to the nucleus in PDAC cells. GSEA was conducted according to the expression level of *DDX31* (Figure 6B). Finally, we found that the pathways that were positively correlated with *DDX31* were mainly enriched in cell adhesion molecules and cell cycle pathways. Then, we performed loss and gain-of-function studies according to the results of Western blot analysis (Figure 6C). BxPC-3-vector/*DDX31*-OE and MIA-PaCa2-vector/*DDX31*-OE cell lines were established, and related functional experiments were performed to elucidate the role of *DDX31* in PDAC. First, the protein expression levels of *DDX31* in BxPC-3-vector/*DDX31*-OE and MIA-PaCa2-vector/*DDX31*-OE cell lines were validated by Western blot analysis. To explore the function by which *DDX31* promotes PDAC cell migration and proliferation, we performed Western blot analysis with some confirmed EMT-related proteins and proliferation-related protein antibodies. As shown in Figure 6C, the proteins N-cadherin, Snail, ZEB1, Ki67, and PCNA were positively correlated with *DDX31* overexpression (Figure 6C, BxPC-3, MIA-PaCa2, L). The opposite results were observed in *DDX31*-knockdown PDAC cells (Figure 6C, SW 1990, L3.7, R). These results revealed that *DDX31* positively regulated these classical EMT and proliferation markers. To determine the role of *DDX31* in cellular migration, wound healing assay and transwell migration assay were performed. Compared with the WT PDAC cells and vector control PDAC cells, the results of transwell assay showed that the migration rate of PDAC cells significantly increased upon *DDX31* overexpression (Figure 6D, BxPC-3, MIA-PaCa2), and the overexpression of *DDX31* also significantly promoted cells’ wound closure (Figure 6F, BxPC-3, MIA-PaCa2). Next, we focused on the effects of *DDX31* knockdown on cellular mobility capacities in SW1990 and L3.7 cell lines (Figures 6E,G; the sequence of DDX31 shRNA was placed in Table 3). By contrast, the opposite results were obtained in *DDX31*-knockdown PDAC cell lines (Figures 6E,G, SW1990 and L3.7). According to the results of transwell assay and wound-closing procedure, the depletion of *DDX31* significantly decreased the capacity of cellular migration. Furthermore, the functional verification work of DDX31 had also conducted in HPDE6c7 (normal pancreatic ductal epithelial cells) (Supplementary Figures S7).

**TABLE 3:** shRNA sequences of *DDX31*.

shRNA sequences for stable knockdown cell lines	
Human *DDX31* shRNA1	CCG​GCC​CTT​CAA​GCA​ATG​GAG​TCA​ACT​CGA​GTT​GAC​TCC​ATT​GCT​TGA​AGG​GTT​TTT​G
Human *DDX31* shRNA2	CCG​GGG​ACA​TCA​CAG​TGA​TAC​TTA​ACT​CGA​GTT​AAG​TAT​CAC​TGT​GAT​GTC​CTT​TTT​G
Human *DDX31* shRNA3	CCG​GGC​TGA​AAT​CCT​ACG​TTC​GGA​ACT​CGA​GTT​CCG​AAC​GTA​GGA​TTT​CAG​CTT​TTT​G

### *DDX31* Promoted Cellular Proliferation and Cell Viability in PDAC Cells

**FIGURE 7:**
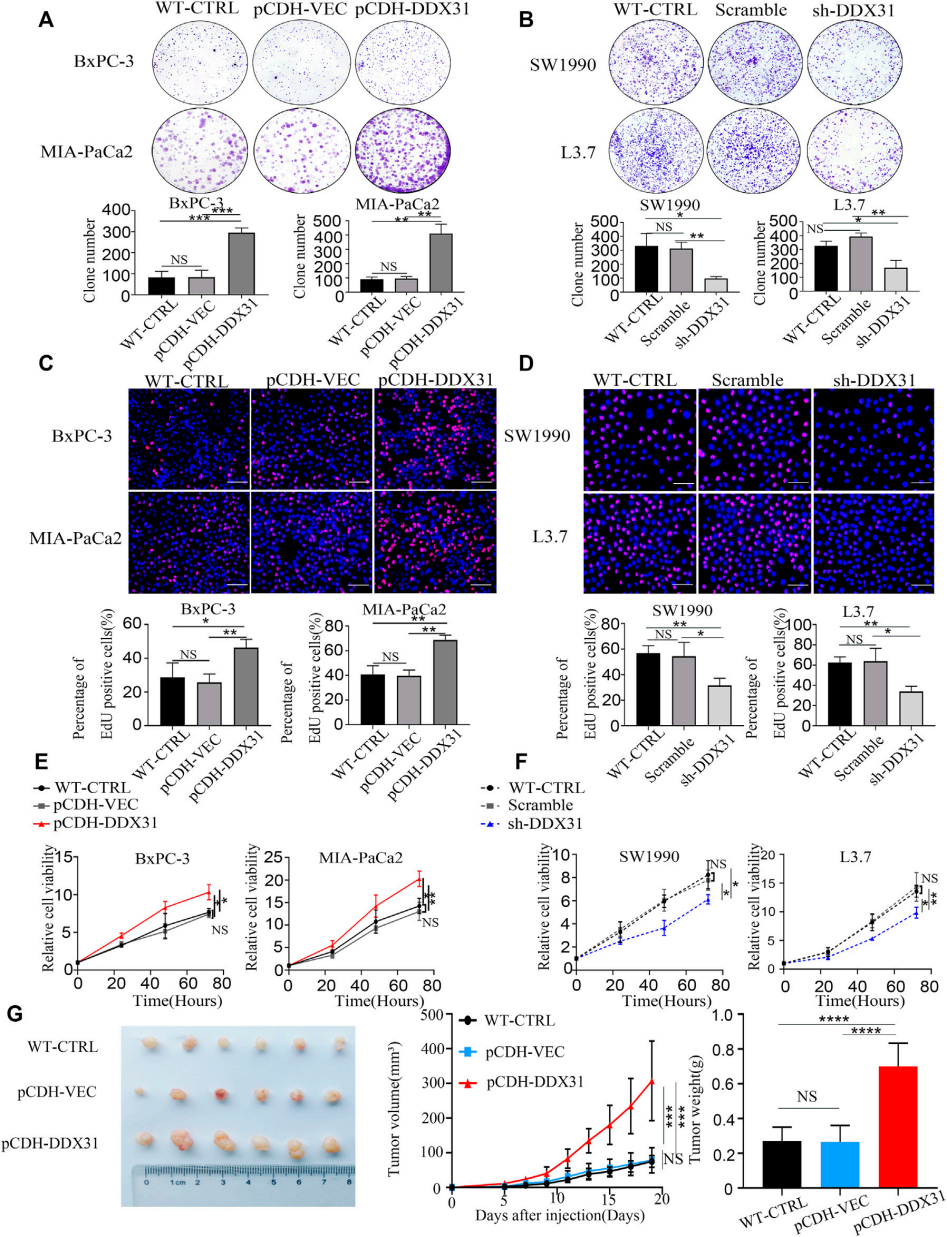
*DDX31* promoted PDAC cell proliferation and cell viability. **(A,D)**: Colony formation assays were performed in the indicated cell lines. Representative images and statistical analysis are shown (overexpression, left; knockdown, right). **(B,E)**: EdU staining assays (bar = 200 μm) were conducted in indicated cell lines. Representative images and statistical analysis are shown. **(C,F)**: CCK-8 assays were performed to test the *DDX31* function on cell viability in indicated cell lines. Representative growth curves are shown. **(G)**: *In vivo* subcutaneous tumor model was used to determine the role of *DDX31* in the cellular proliferation of BxPC-3 (infected with pCDH-*DDX31* and pCDH-VECTOR lentivirus). Mice were sacrificed after 19 days; the volume and mass of harvested tumors were measured three times a week. Each experiment was independently repeated three times, and the representative results are shown. Unpaired Student’s t-test was used for **(A–G)** analysis. *p < 0.05, **p < 0.01, and NS means non significant.

Crystal violet staining was conducted to evaluate the clone formation ability. *DDX31*-overexpressing PDAC cell lines demonstrated a greater number of colonies than the vector control and WT control (Figure 7A). EdU staining assays were also performed. As shown in Figure 7B, PDAC-*DDX31*-OE showed a higher percentage of EdU-positive cancer cells. This finding indicated that *DDX31* overexpression led to an increased cell proliferation capability in *DDX31*-OE PDAC cells (BxPC-3/MIA-PaCa2) compared with the WT-CTRL/pCDH-VEC control group. In addition, the overexpression of *DDX31* promoted the cell viability of BxPC-3 and MIA-PaCa2 cells by CCK-8 assay (Figure 7C). To further confirm the proliferation oncogene function of *DDX31*, clone formation and EDU stain assay was performed. The results revealed that *DDX31* downregulation suppressed the proliferation ability of *DDX31*-knockdown PDAC cells (SW1990/L3.7) (Figures 7D,E). However, the downregulation of *DDX31* obviously inhibited the cell viability of SW1990/L3.7 cells compared with the scramble group and WT PDAC cells by CCK-8 assay (Figure 7F). To further identify the role of *DDX31* in PDAC proliferation, *in vivo* the mouse xenograft experiment were used. Subcutaneous tumors in nude mice were formed by BxPC-3 infected with pCDH-*DDX31*, pCDH-VECTOR lentivirus, and WT-CTRL PDAC cell lines, and we isolated tumors after 19 and 5 days. As shown in Figure 7G, the results revealed that *DDX31* overexpression significantly promoted tumor growth.

### *DDX31* Affects Invasive Metastasis and Proliferation of Pancreatic Cancer by Activating MAPK Signaling Pathway

**FIGURE 8:**
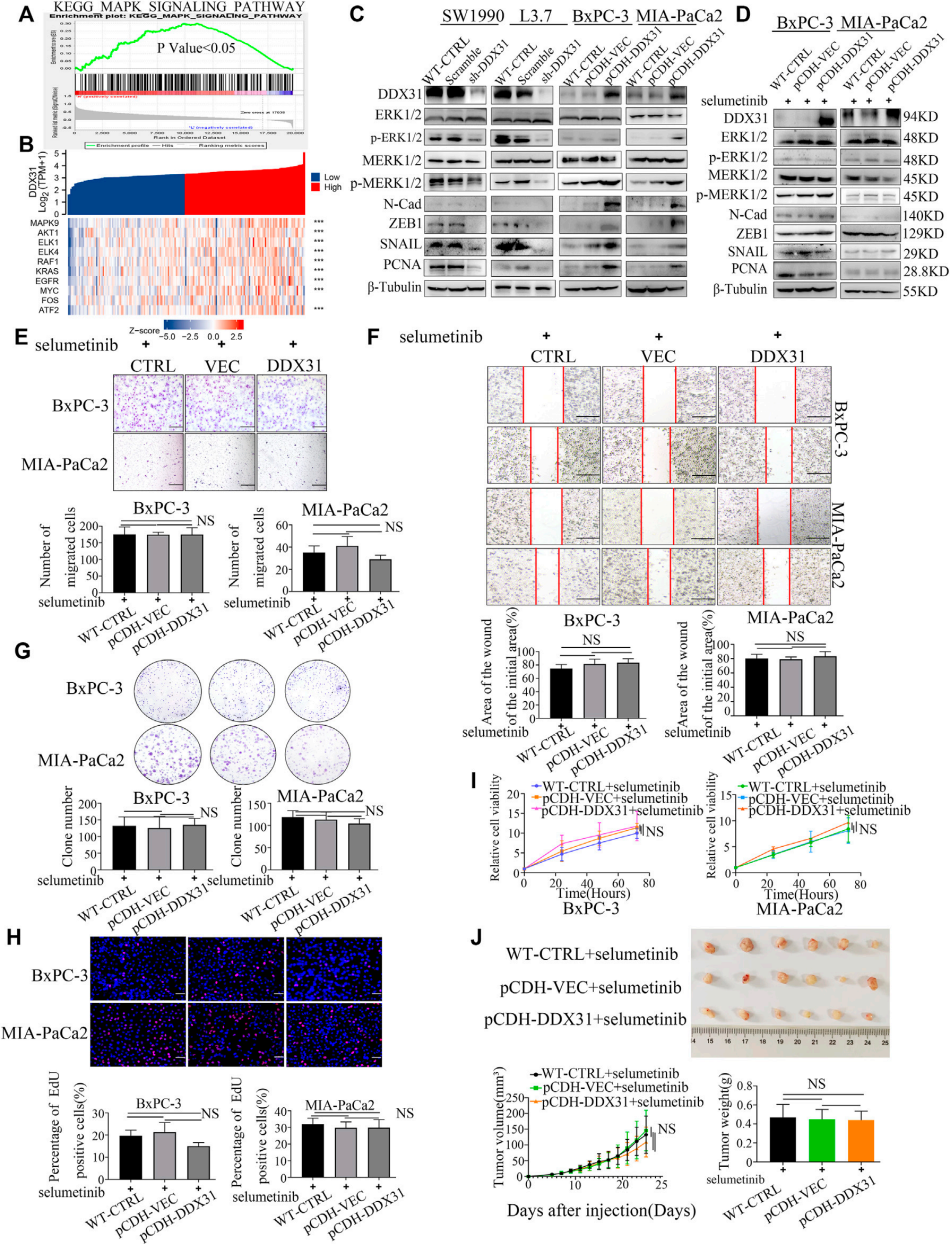
*DDX31* affects invasive metastasis and proliferation of pancreatic cancer by activating MAPK signaling pathway. **(A)**: GSEA was carried out according to the expression level of *DDX31*; p value < .05, FDR <0.25; **(B)**: Correlation analysis between DDX31 and the core genes of the MAPK signaling pathway (MAPK9, AKT1, ELK1, ELK4, RAF1, KRAS, EGFR, MYC, FOS); the results were shown with heat map; p value < .05; **(C)**: The protein expression of the core molecules in MAPK signaling pathway (ERK1/2, P-ERK1/2 and P38, P-P38) within loss and gain-of *DDX31* (*DDX3*1 overexpression on BxPC-3/MIA-PaCa2 cell lines, DDX31 knockdown on SW1990/L3.7 cell lines). EMT-related and proliferation markers (N-cadherin, Snail, ZEB1, and PCNA) were also showed according to the results of Western blot analysis. **(D)**: Effect of MAPK pathway inhibitor on PDAC cell lines of *DDX31* overexpression was shown with western blot assays. **(E,F)**: Cell migration was detected by transwell assays (bar = 200 μm) and wound healing experiments (bar = 200 μm) in *DDX31*-overexpression PDAC cell lines BxPC-3 and MIA-PaCa2 by giving MAPK pathway inhibitor (selumetinib). Representative images and statistical analysis are shown. **(G)**: Colony formation assays were performed in *DDX31*-overexpression PDAC cell lines BxPC-3 and MIA-PaCa2 by giving MAPK pathway inhibitor (selumetinib). Representative images and statistical analysis are shown. **(H)**: EDU staining assays (bar = 200 μm) were conducted in DDX31-overexpression PDAC cell lines BxPC-3 and MIA-PaCa2 by giving MAPK pathway inhibitor (selumetinib). **(I)**: the cell viability of BxPC-3 and MIA-PaCa2 cells were detected in the overexpression of *DDX31* by CCK-8 assay. **(J)**: Subcutaneous tumors in nude mice were estimated in *DDX31*-overexpression PDAC cell lines BxPC-3 by giving MAPK pathway inhibitor (selumetinib), and the inhibitor of MAPK signaling pathway was intraperitoneally injected three times a week; Representative images and statistical analysis are shown. Images were collected three times randomly from each experiment. The figures at the bottom show the corresponding statistical chart (unpaired t-tests). Values are presented as the means ± SDs of three independent experiments. *p < .05, **p < .01, ***p < .001, ****p < .0001, and NS means non significant.

To explore how the *DDX31* affects invasive metastasis and proliferation in PDAC cells, the GSEA was carried out according to the expression level of *DDX31* (Figure 8A). We found that MAPK signaling pathway was significantly enriched in high expression of *DDX31*. And then, correlation analysis between *DDX31* and the core genes of the *MAPK* signaling pathway were shown with heatmap (Figure 8B and Supplementary Figures S8A,B); the results indicated that *DDX31* was significantly positively correlated with these core genes *(MAPK9, AKT1, ELK1,- ELK4, RAF1, KRAS, EGFR, MYC, FOS, ATF2* and so on) which played vital roles in *MAPK* signaling pathway (Figure 8B); for further figuring out the link between *DDX31* and MAPK signaling pathway; *DDX31*-OE PDAC cells (BxPC-3/MIA-PaCa2) and *DDX31*-knockdown PDAC cells (SW1990/L3.7) were chosen to performed the western blot assays; the results of western blot assays revealed that *P-ERK1/2, P-P38*, EMT-related markers and proliferation markers (N-cadherin, Snail, ZEB1, and PCNA) were elevated with high expression of *DDX31*. To further validate the fact, the inhibitor of MAPK signaling pathway (selumetinib) was used for blocking to reverse the function of *DDX31*; finally, we found that the proteins *P-ERK1/2, P-P38*, EMT-related markers and proliferation markers (N-cadherin, *Snail, ZEB1, and PCNA*) in PDAC cells were consistently changed with the use of inhibitor of MAPK signaling pathway. The results indicated that *DDX31* exerted the function of invasive metastasis and proliferation were probably dependent on *MAPK* signaling pathway; for further validating the results, wound healing assay and transwell migration assay were performed. Compared with the WT PDAC cells and vector control PDAC cells, the results of transwell assay showed that the migration rate of PDAC cells did not significantly change upon *DDX31* overexpression by using the inhibitor of *MAPK* signaling pathway (Figure 8E, BxPC-3, MIA-PaCa2), and the PDAC cells’ wound closure showed the same results (Figure 8F, BxPC-3, MIA-PaCa2). According to the results of transwell assay and wound-closing procedure, the inhibitor of *MAPK* signaling pathway significantly blocked the function of *DDX31*. Crystal violet staining was conducted to evaluate the clone formation ability. *DDX31*-overexpressing PDAC cell lines demonstrated no obvious difference of colonies than the vector control and WT control by using the inhibitor of MAPK signaling pathway (Figure 8G). EdU staining assays were also performed. As shown in Figure 8H, PDAC-*DDX31*-OE did not show a higher percentage of EdU-positive cancer cells. This finding indicated that *DDX31* overexpression did not increased cell proliferation capability in *DDX31*-OE PDAC cells (BxPC-3/MIA-PaCa2) compared with the WT-CTRL/pCDH-VEC control group in the case of simultaneous use of the inhibitor of MAPK signaling pathway. In addition, the overexpression of *DDX31* did not change the cell viability of BxPC-3 and MIA-PaCa2 cells by CCK-8 assay (Figure 8I). To further identify the role of *DDX31* in PDAC proliferation, *in vivo* mouse xenograft experiment were used. Subcutaneous tumors in nude mice were formed by BxPC-3 infected with pCDH-*DDX31*, pCDH-VECTOR lentivirus, and WT-CTRL PDAC cell lines, and the inhibitor of MAPK signaling pathway was intraperitoneally injected three times a week; finally, we isolated tumors after 19 and 5 days. As shown in Figure 8J, the results revealed that *DDX31* overexpression did not show significant differences of promoting tumor growth in the case of simultaneous use of the inhibitor of MAPK signaling pathway.

## Discussion

Due to the proliferation and invasion of tumor, the treatment and diagnosis of PDAC face great challenges (Chen et al., 2019). The high proliferation capacity of PDAC leads to rapid tumor growth, which further promotes tumor metastasis and eventually leads to poor survival prognosis of PDAC patients (Hosein et al., 2020). Therefore, it is extremely urgent for us to find an effective therapeutic target to elucidate the molecular mechanism of PDAC proliferation and invasion.

In our study, a total 109 intersected DEGs were obtained by systematic analysis of multiple center datasets. Then, GO and KEGG pathway enrichment analysis were performed and indicated that the screened DEGs are associated with pathways of Cadherin Binding involved in cell-cell Adhesion, Ephrin Receptor Binding, and Proline-Rich Region Binding. Furthermore, Lasso regression analysis was performed to screen out four genes and these four genes were used to build a four-gene signature as a prognostic risk model. Moreover, the effectiveness and reliability of this risk model were tested in GEO dataset. Subsequently, WGCNA analysis was employed and we found that DDX31 was involved in liver and lymphatic invasion of PDAC. And then, we found DDX31 was tightly correlated with invasive metastasis and proliferation in PDAC cell lines by activating MAPK signaling pathway *in vivo* and vitro experiments.

CAP1, adenylate cyclase-associated protein 1, The protein encoded by this gene is related to the S. cerevisiae CAP protein, which is involved in the cyclic AMP pathway. The human protein is able to interact with other molecules of the same protein, as well as with CAP2 and actin. Alternatively spliced transcript variants have been identified (Schneider et al., 2021). The expression of CAP1 in non-small-cell lung cancer increased during growth of the primary tumor (Kolegova et al., 2021). besides, the Phosphorylation of CAP1 could promote the proliferation, migration and invasion in lung cancer (Zeng et al., 2022). Moreover, cap1 has been identified as a potential biomarker by the analysis of the pancreatic cancer proteome (Agrawal, 2017). CD59 is a glycosylphosphatidylinositol (GPI)-anchored membrane protein that could regulate the complement activation by preventing C9 from polymerizing and forming the membrane attack complex (Zhang et al., 2018). CD59 has been reported to be highly expressed in patients with pancreatic cancer (Schmitt et al., 1999), and Pancreatic cancer-educated macrophages could up-regulate CD59 to protect cancer cells from CDC through the IL-6R/STAT3 signaling pathway (Ronghua Zhang et al., 2019). YAP1, The Hippo-yes-associated protein (YAP) pathway, plays an important role in modulating metabolism (Zhao et al., 2018), tumorigenesis (McClelland et al., 1990). YAP1 is a transcriptional coactivator of the Hippo pathway, which can reduce the activity of YAP1 by promoting cytoplasmic localization of YAP1 (Shen et al., 2018). Hippo-YAP/TAZ signaling is a critical factor in tumor growth and metastasis (Zhang et al., 2009); particularly, YAP1 increase the ability of EMT and invasion of breast epithelial cells (Overholtzer et al., 2006). Activation of YAP1 could mediate Epithelial-to-Mesenchymal transition in Triple-Negative Breast Cancer (Kim et al., 2021). DEAD box protein family members have attracted considerable attention in recent years, and their functions in cancer pathogenesis and development were reported in previous studies.

DDX1 was first found in human neuroblastoma and retinoblastoma cell lines in 1998 (Godbout et al., 1998) and it was reported in cervical carcinoma cells in 2001, its’ location pattern in the nucleus of HeLa, this study also found that DDX1 was involved in the 3-end cleavage and polyadenylation of pre-mRNAs (Bléoo et al., 2001). In 2018, Tanaka K et al. found that DDX1 could promotes colorectal tumorigenesis through activation of the LGR5. DDX2 (also known as eIF4A) was reported as the key factor promoting the progression of T-cell acute lymphoblastic leukaemia and inhibition of DDX2 could effectively kill human leukemic cells (Wolfe et al., 2014). DDX5 (also known as p68) was identified by Mirsada Causevic that it was over-expressed in colorectal cancer (Causevic et al., 2001). In 1998, DDX6 (also known as rck/p54) was found high expression in human colorectal tumor tissues, but low expression in normal colorectal mucosa tissues (Nakagawa et al., 1999). The next year, DDX9 was found and mapped to the prostate cancer susceptibility locus at chromosome band 1q25 (Lee et al., 1999). DDX43 (also known as HAGE) had been reported as a promising biomarker indicated poor prognosis in breast cancer (Abdel-Fatah et al., 2014). Qing Xia and Xian-Tao Kong made the serological evaluation in 60 patients with pancreatic cancer, and other 200 individuals as control, including 30 with colorectal cancer, 30 with gastric cancer, 30 with hepatocellular cancer, 30 with lung cancer, 20 with chronic pancreatitis, and 60 healthy volunteers in 2005. Their results showed that DDX48 antibody reactivity 33.33% in pancreatic cancer patients, 10.00% in colorectal cancer patients, 6.67% in gastric cancer patients, and 6.67% in hepatocellular cancer patients (Xia et al., 2005). DDX53 was studied in drug-resistance, Youngmi Kim and et al. proved that DDX53 overexpression enhance the refractory to taxol in cervix cancer cells (Park et al., 2018). DDX56 was published as a functional oncogene which promote the early squamous cell lung cancer recurrence through miRNA modulating Wnt signaling pathway (Wu et al., 2021).

In 2014, Bish and Vogel (2014) found that DDX31 is involved in the growth and maintenance of medulloblastoma. In 2018, Daizumoto et al. (2018) demonstrated that DDX31 plays an essential role in the progression of muscle-invasive bladder cancer (MIBC) cooperates with mutp53 and EGFR, leading to migration and invasion. Moreover, DDX31 has also been reported to reduce HDM2 binding to p53 and affect cell cycle and apoptosis in renal cell carcinoma (Fukawa et al., 2012). These evidences indicate that DDX31 can help drive tumor progression and metastasis, and it also proves the rationality of our screening results. Subsequently, we conducted an in-depth study on whether DDX31 affects the proliferation and transfer capacity of PDAC.

In our study, DDX31 might be a potential risk factor affecting poor survival status of pancreatic cancer patients by univariate (HR = 1.517; CI = 1.003–2.296) and multivariate (HR = 1.229; CI = 0.795–1.900) cox regression analysis, but not significantly statistical difference in multivariate cox regression analysis. With respect to DDX31's HR value partially crossed 1, we found in the analysis results that a small number of patients with high expression of DDX31 had HR less than 1, but the analysis results of most patients showed that it might be a gene that promotes the risk of pancreatic cancer. we 'll explore the reasons for its low risk in subsequent experiments. Besides, what we need to mention here is that among the 86 clinical samples, in 9 of them, the expression level of DDX31 in the para-cancer tissues was higher than that in the cancer tissues, which we speculated should be caused by the heterogeneity between tumor samples or due to the lack of copy number variation in some tumor patients, we still need to further explore the effect of DDX31 genome level changes on transcriptional level and post-transcriptional translation (Figure 5B; Supplementary Figure S5). Then further investigation need to be conducted to explore it *in vivo* and vitro experiments. We first evaluated the association between DDX31 expression and OS/RFS in patients with PDAC and found a significant negative correlation between high DDX31 expression and the patients’ OS/RFS (Figures 5F,G). Furthermore, GO/KEGG enrichment analysis was conducted, and the results showed that the expression of DDX31 was positively correlated with proliferation and EMT in PDAC patients. Therefore, the validation was performed at the cell-line and the mouse xenograft experiment level. Overexpression of DDX31 in BxPC-3 and MIA PaCa-2 cell lines showed upregulated expression of N-cadherin (EMT-related marker), Snail (EMT-related marker), Ki67(proliferation-related marker), and PCNA (proliferation-related marker) (Figure 6C), as well as enhanced proliferation and invasion *in vivo* and *in vitro* (Figures 6D,G; Figures 7A,F). In contrast, low expression of DDX31 showed the opposite trend in SW1990 and L3.7 cell lines. Moreover, we found that overexpression of DDX31 in normal pancreatic cell lines (HPDE6c7) also significantly enhanced cell proliferation and migration (Supplementary Figure S7), suggesting that DDX31 can indeed promote the development of pancreatic cancer. All these phenotypic experiments demonstrated that DDX31 could indeed promote proliferation and invasion of PDAC. After that, we carried out the exploration of the mechanism.

Through gene set enrichment analysis (GSEA), we discovered that the MAPK signaling pathway was significantly positively correlated with DDX31 expression. Therefore, we postulated that the MAPK signaling pathway might be the downstream of DDX31 (Figures 8A,B). As is well known, MAPKs are a family of serine/threonine kinases, regulate a variety of cellular functions, including proliferation, apoptosis, and EMT (Guo et al., 2020), and also play critical roles in intracellular signal transduction in cells (de Leeuw et al., 2018). In our study, we found that DDX31 promotes the phosphorylation of ERK1/2 and MERK1/2, which may lead to increased tumor proliferation and invasion (Figures 8C,D). To further verify our hypothesis, we performed a blocking experiment using MAPK/MERK/ERK pathway inhibitor. The results showed that DDX31 overexpression induced cell proliferation and invasion were significantly inhibited compared with those without MAPK/MERK/ERK signaling pathway inhibitor (Figures 6D,G; Figures 7A,G; Figures 8E,J). Besides, we obtained the same conclusion through *in vivo* validation by conducting a tumor formation assay in mouse model. These evidences suggested that DDX31 promotes PDAC proliferation and invasion via the MAPK/MERK/ERK signaling pathway.

Upon validation using bioinformatics analysis and cell biology experiments, we confirmed that DDX31 could promote proliferation and invasion of PDAC cells *via* the MAPK/MERK/ERK signaling pathway. Our results indicate that DDX31 may be a potential therapeutic target and a promising biomarker for assessing the prognosis for patients with PDAC. Through our study, we identified DDX31 as a new research target, thereby providing a useful reference for PDAC proliferation and invasion. But we still have many problems need to be further figured out. DDX31 was absent in 25% of pancreatic cancer patients, and its expression in some cancer tissues was similar to that in paracancerous tissues. We speculate that there may be other modifications with it in the nucleus, or other effects at the transcriptional level, which we need to further explore in subsequent studies.

## Data Availability

The original contributions presented in the study are included in the article/Supplementary Material, further inquiries can be directed to the corresponding author.

## Supplementary Material

Supplementary Figure S1 The work flowchart of analysis. The concrete and distinct work flowchart of this research was presented.

Supplementary Figure S2 DEG analysis of TCGA and GEO datasets. **(A–C)**: Up- and downregulated DEGs are shown in the volcano plot. **(D)**: Unsupervised clustering of viable cells in the KPC-SCRNA dataset. **(E)**: DEGs between ductal cells and acinar cells are shown in the heat map.

Supplementary Figure S3 Validation of the prediction ability of the risk model. **(A)**: Survival and prognosis of patients in high- and low-risk groups were verified in the GSE62452 and GSE102238 dataset. Red represents the high-risk group, and blue represents the low-risk group. **(B)**: Time-dependent ROC curve prediction analysis of 1-, 3-, and 5-year risk scores. **(C)**: 1-, 3-, and 5-year predictive ability of risk score in the nomogram. **(D)**: 1-, 3-, and 5-year prediction ability of risk score with calibration analysis. **(E)**: DCA of the risk model. **(F)**: Univariate and multivariate cox regression analysis of risk model in GEO datasets.

Supplementary Figure S4 Screening of key phenotypic module and key genes by WGCNA. **(A)**: Hierarchical clustering dendrogram of samples from TCGA database. **(B)**: Analysis of scale-free fit index and the mean connectivity for various soft-thresholding powers. Testing the scale-free topology when *β* = 9. **(C)**: Hierarchical clustering dendrogram of genes with dissimilarity on the basis of topological overlap. Modules are the branches of the clustering tree. **(D)**: Correlation between module eigengenes and clinical traits. Each row corresponds to a module eigengene, and columns represent clinical traits. Each cell contains the correlation and p value, and the yellow module containing DDX31 is selected. The red bar represents positive correlation, while the blue bar represents negative correlation. **(E)**: Gene correlation analysis within the expression of blue module. **(F)**: The heat map describes the TOM among the 2544 genes selected in WGCNA. Gene modules are represented by horizontal and vertical coordinates, and highly overlapping modules are marked by high-bright parts.

Supplementary Figure S5 Single-nucleotide variation (SNV) and copy number variation (CNV) of targeted genes in TCGA samples. **(A)**: Percentage and types of SNV in TCGA samples. **(B)**: Top ranking genes with high percentage of SNV in high-risk score group of TCGA samples. **(C)**: The percentage of CNV of *CAP1*, *CD59*, *DDX31*, and *YAP1* in PDAC samples of TCGA.

Supplementary Figure S6 Immunofluorescence of *DDX31* in PC cell lines. **(A)**: Immunofluorescence of *DDX31* in SW1990 and MIA-PaCa2 cell lines.

Supplementary Figure S7 The functional verification work of DDX31 in HPDE6c7 (normal pancreatic ductal epithelial cells).

Supplementary Figure S8 The spearman’s correlation analysis between *DDX31* and the core molecules in *MAPK* signaling pathway. **(A)**: The spearman’s correlation analysis between *DDX31* and the top50 genes in *MAPK* signaling pathway; **(B)**: The spearman’s correlation analysis between *DDX31* and the top50 to top100 genes in *MAPK* signaling pathway; p value<.001; R>.3.

## References

[B1] Abdel-Fatah T. M. A. McArdle S. E. B. Johnson C. Moseley P. M. Ball G. R. Pockley A. G. (2014). HAGE (DDX43) Is a Biomarker for Poor Prognosis and a Predictor of Chemotherapy Response in Breast Cancer. Br. J. Cancer 110 (10), 2450–2461. 10.1038/bjc.2014.168 24755885 PMC4021517

[B2] Abdelhaleem M. (2004). Over-Expression of RNA Helicases in Cancer. Anticancer Res. 24 (6), 3951–3953. 15736437

[B3] Agrawal S. (2017). Potential Prognostic Biomarkers in Pancreatic Juice of Resectable Pancreatic Ductal Adenocarcinoma. Wjco 8 (3), 255–260. 10.5306/wjco.v8.i3.255 28638795 PMC5465015

[B4] Anderson E. M. Thomassian S. Gong J. Hendifar A. Osipov A. (2021). Advances in Pancreatic Ductal Adenocarcinoma Treatment. Cancers 13 (21), 5510. 10.3390/cancers13215510 34771675 PMC8583016

[B5] Andolino C. Hess C. Prince T. Williams H. Chernin M. (2018). Drug-induced Keratin 9 Interaction with Hsp70 in Bladder Cancer Cells. Cell Stress Chaperones 23 (5), 1137–1142. 10.1007/s12192-018-0913-2 29802537 PMC6111075

[B6] Balachandran V. P. Beatty G. L. Dougan S. K. (2019). Broadening the Impact of Immunotherapy to Pancreatic Cancer: Challenges and Opportunities. Gastroenterology 156 (7), 2056–2072. 10.1053/j.gastro.2018.12.038 30660727 PMC6486864

[B7] Barros-Silva J. D. Linn D. E. Steiner I. Guo G. Ali A. Pakula H. (2018). Single-Cell Analysis Identifies LY6D as a Marker Linking Castration-Resistant Prostate Luminal Cells to Prostate Progenitors and Cancer. Cel. Rep. 25 (12), 3504–3518.e6. 10.1016/j.celrep.2018.11.069 PMC6315111 30566873

[B8] Bish R. Vogel C. (2014). RNA Binding Protein-Mediated post-Transcriptional Gene Regulation in Medulloblastoma. Mol. Cell 37 (5), 357–364. 10.14348/molcells.2014.0008 PMC4044306 24608801

[B9] Blanche P. Dartigues J.-F. Jacqmin-Gadda H. (2013). Estimating and Comparing Time-Dependent Areas under Receiver Operating Characteristic Curves for Censored Event Times with Competing Risks. Statist. Med. 32 (30), 5381–5397. 10.1002/sim.5958 24027076

[B10] Bléoo S. Sun X. Hendzel M. J. Rowe J. M. Packer M. Godbout R. (2001). Association of Human DEAD Box Protein DDX1 with a Cleavage Stimulation Factor Involved in 3′-End Processing of Pre-mRNA. MBoC 12 (10), 3046–3059. 10.1091/mbc.12.10.3046 11598190 PMC60154

[B11] Causevic M. Hislop R. G. Kernohan N. M. Carey F. A. Kay R. A. Steele R. J. C. (2001). Overexpression and Poly-Ubiquitylation of the DEAD-Box RNA Helicase P68 in Colorectal Tumours. Oncogene 20 (53), 7734–7743. 10.1038/sj.onc.1204976 11753651

[B12] Chen H. Li L. Hu J. Zhao Z. Ji L. Cheng C. (2019). UBL4A Inhibits Autophagy-Mediated Proliferation and Metastasis of Pancreatic Ductal Adenocarcinoma via Targeting LAMP1. J. Exp. Clin. Cancer Res. 38 (1), 297. 10.1186/s13046-019-1278-9 31288830 PMC6617940

[B13] Dai S. Zhang J. Huang S. Lou B. Fang B. Ye T. (2017). HNRNPA2B1 Regulates the Epithelial-Mesenchymal Transition in Pancreatic Cancer Cells through the ERK/snail Signalling Pathway. Cancer Cel. Int. 17, 12. 10.1186/s12935-016-0368-4 PMC5223355 28077929

[B14] Daizumoto K. Yoshimaru T. Matsushita Y. Fukawa T. Uehara H. Ono M. (2018). A DDX31/Mutant-P53/EGFR Axis Promotes Multistep Progression of Muscle-Invasive Bladder Cancer. Cancer Res. 78 (9), 2233–2247. 10.1158/0008-5472.can-17-2528 29440146

[B15] de Leeuw R. McNair C. Schiewer M. Neupane N. Brand L. Augello M. (2018). MAPK Reliance via Acquired CDK4/6 Inhibitor Resistance in Cancer. Clin. Cancer Res. : official J. Am. Assoc. Cancer Res. 24 (17), 4201–4214. 10.1158/1078-0432.ccr-18-0410 PMC6125187 29739788

[B16] Dey S. Liu S. Factora T. Taleb S. Riverahernandez P. Udari L. (2020). Global Targetome Analysis Reveals Critical Role of miR-29a in Pancreatic Stellate Cell Mediated Regulation of PDAC Tumor Microenvironment. BMC cancer 20 (1), 651. 10.1186/s12885-020-07135-2 32660466 PMC7359459

[B17] Elyada E. Bolisetty M. Laise P. Flynn W. F. Courtois E. T. Burkhart R. A. (2019). Cross-Species Single-Cell Analysis of Pancreatic Ductal Adenocarcinoma Reveals Antigen-Presenting Cancer-Associated Fibroblasts. Cancer Discov. 9 (8), 1102–1123. 10.1158/2159-8290.cd-19-0094 31197017 PMC6727976

[B18] Fenocchio E. Filippi R. Lombardi P. Quarà V. Milanesio M. Aimar G. (2019). Is There a Standard Adjuvant Therapy for Resected Pancreatic Cancer? Cancers 11 (10), 1547. 10.3390/cancers11101547 PMC6826876 31614884

[B19] Friedman J. Hastie T. Tibshirani R. (2010). Regularization Paths for Generalized Linear Models via Coordinate Descent. J. Stat. Softw. 33 (1), 1–22. 10.18637/jss.v033.i01 20808728 PMC2929880

[B20] Fukawa T. Ono M. Matsuo T. Uehara H. Miki T. Nakamura Y. (2012). DDX31 Regulates the P53-HDM2 Pathway and rRNA Gene Transcription through its Interaction with NPM1 in Renal Cell Carcinomas. Cancer Res. 72 (22), 5867–5877. 10.1158/0008-5472.can-12-1645 23019224

[B21] Godbout R. Packer M. Bie W. (1998). Overexpression of a DEAD Box Protein (DDX1) in Neuroblastoma and Retinoblastoma Cell Lines. J. Biol. Chem. 273 (33), 21161–21168. 10.1074/jbc.273.33.21161 9694872

[B22] Guo Y. Pan W. Liu S. Shen Z. Xu Y. Hu L. (2020). ERK/MAPK Signalling Pathway and Tumorigenesis. Exp. Ther. Med. 19 (3), 1997–2007. 10.3892/etm.2020.8454 32104259 PMC7027163

[B23] Guy S. Sermon-Cadd A. Shepherd F. Kitchen S. Bowyer A. (2019). A Cost-Effective Approach to Factor Assay Calibration Using a Truncated Live Calibration Curve. Int. J. Lab. Hematol. 41 (5), 679–683. 10.1111/ijlh.13087 31421012

[B24] Hayashi A. Hong J. Iacobuzio-Donahue C. A. (2021). The Pancreatic Cancer Genome Revisited. Nat. Rev. Gastroenterol. Hepatol. Gastroenterol. Hepatol. 18 (7), 469–481. 10.1038/s41575-021-00463-z 34089011

[B25] Hosein A. Brekken R. Maitra A. (2020). Pancreatic Cancer Stroma: an Update on Therapeutic Targeting Strategies. Nat. rev. Gastroenterol. Hepatol. 17 (8), 487–505. 10.1038/s41575-020-0300-1 32393771 PMC8284850

[B26] Ito K. Murphy D. (2013). Application of Ggplot2 to Pharmacometric Graphics. CPT: Pharmacometrics Syst. Pharmacol. 2 (10), 79. 10.1038/psp.2013.56 PMC3817376 24132163

[B27] Jankowsky E. (2011). RNA Helicases at Work: Binding and Rearranging. Trends Biochem. Sci. 36 (1), 19–29. 10.1016/j.tibs.2010.07.008 20813532 PMC3017212

[B28] Kanehisa M. Goto S. (2000). KEGG: Kyoto Encyclopedia of Genes and Genomes. Nucleic Acids Res. 28 (1), 27–30. 10.1093/nar/28.1.27 10592173 PMC102409

[B29] Kim J. Jang G. Sim S. Park I. Kim K. Park C. (2021). SMARCA4 Depletion Induces Cisplatin Resistance by Activating YAP1-Mediated Epithelial-To-Mesenchymal Transition in Triple-Negative Breast Cancer. Cancers 13 (21), 5474. 10.3390/cancers13215474 34771636 PMC8582548

[B30] Klein A. P. (2021). Pancreatic Cancer Epidemiology: Understanding the Role of Lifestyle and Inherited Risk Factors. Nat. Rev. Gastroenterol. Hepatol. 18 (7), 493–502. 10.1038/s41575-021-00457-x 34002083 PMC9265847

[B31] Kolegova E. Kakurina G. Shashova E. Yunusova N. Spirina L. Sidenko E. (2021). Relationship of Intracellular Proteolysis with CAP1 and Cofilin1 in Non-Small-Cell Lung Cancer. J. Biosci. 46, 55. 10.1007/s12038-021-00177-z 34148878

[B32] Lee C. Eki T. Okumura K. Nogami M. Soares V. C. Murakami Y. (1999). The Human RNA Helicase A (DDX9) Gene Maps to the Prostate Cancer Susceptibility Locus at Chromosome Band 1q25 and its Pseudogene (DDX9P) to 13q22, Respectively. Somatic Cel. Mol. Genet. 25 (1), 33–39. 10.1023/b:scam.0000007138.44216.3a 10925702

[B33] Linder P. Jankowsky E. (2011). From Unwinding to Clamping - the DEAD Box RNA Helicase Family. Nat. Rev. Mol. Cel. Biol. 12 (8), 505–516. 10.1038/nrm3154 21779027

[B34] McClelland R. Finlay P. Walker K. Nicholson D. Robertson J. Blamey R. (1990). Automated Quantitation of Immunocytochemically Localized Estrogen Receptors in Human Breast Cancer. Cancer Res. 50 (12), 3545–3550. 2187598

[B35] Nakagawa Y. Morikawa H. Hirata I. Shiozaki M. Matsumoto A. Maemura K. (1999). Overexpression of Rck/p54, a DEAD Box Protein, in Human Colorectal Tumours. Br. J. Cancer 80, 914–917. 10.1038/sj.bjc.6690441 10360675 PMC2362290

[B36] Neoptolemos J. P. Springfeld C. Hackert T. (2021). A Review of Pancreatic Cancer. JAMA 326 (23), 2436. 10.1001/jama.2021.20065 34932083

[B37] Nguyen T. Hamada A. Yamada K. Higaki M. Shintani T. Yoshioka Y. (2021). Enhanced KRT13 Gene Expression Bestows Radiation Resistance in Squamous Cell Carcinoma Cells. Vitro Cell. Dev. Biol. Anim. 57 (3), 300–314. 10.1007/s11626-020-00542-6 33537930

[B38] Overholtzer M. Zhang J. Smolen G. Muir B. Li W. Sgroi D. (2006). Transforming Properties of YAP, a Candidate Oncogene on the Chromosome 11q22 Amplicon. Proc. Natl. Acad. Sci. United States Am. 103 (33), 12405–12410. 10.1073/pnas.0605579103 PMC1533802 16894141

[B39] Park S. Kim W. Byun J. Lee J. Jeoung D. Park S. (2018). Role of DDX53 in Taxol-Resistance of Cervix Cancer Cells *In Vitro* . Biochem. Biophys. Res. Commun. 506 (3), 641–647. 10.1016/j.bbrc.2018.10.145 30454700

[B40] Ritchie M. E. Phipson B. Wu D. Hu Y. Law C. W. Shi W. (2015). Limma powers Differential Expression Analyses for RNA-Sequencing and Microarray Studies. Nucleic Acids Res. 43 (7), e47. 10.1093/nar/gkv007 25605792 PMC4402510

[B42] Schmitt C. Schwaeble W. Wittig B. Meyer zum Büschenfelde K. Dippold W. (1999). Expression and Regulation by Interferon-Gamma of the Membrane-Bound Complement Regulators CD46 (MCP), CD55 (DAF) and CD59 in Gastrointestinal Tumours. Eur. J. Cancer 35 (1), 117–124. 10.1016/s0959-8049(98)00290-1 10211099

[B43] Schneider F. Metz I. Khudayberdiev S. Rust M. (2021). Functional Redundancy of Cyclase-Associated Proteins CAP1 and CAP2 in Differentiating Neurons. Cells 10 (6), 1525. 10.3390/cells10061525 34204261 PMC8234816

[B44] Shen J. Cao B. Wang Y. Ma C. Zeng Z. Liu L. (2018). Hippo Component YAP Promotes Focal Adhesion and Tumour Aggressiveness via Transcriptionally Activating THBS1/FAK Signalling in Breast Cancer. J. Exp. Clin. Cancer Res. 37 (1), 175. 10.1186/s13046-018-0850-z 30055645 PMC6064138

[B46] Shintani Y. Fukumoto Y. Chaika N. Grandgenett P. Hollingsworth M. Wheelock M. (2008). ADH-1 Suppresses N-cadherin-dependent Pancreatic Cancer Progression. Int. J. Cancer 122 (1), 71–77. 10.1002/ijc.23027 17721921

[B47] Singhi A. D. Wood L. D. (2021). Early Detection of Pancreatic Cancer Using DNA-Based Molecular Approaches. Nat. Rev. Gastroenterol. Hepatol. Gastroenterol. Hepatol. 18 (7), 457–468. 10.1038/s41575-021-00470-0 34099908

[B48] Sodir N. Kortlever R. Barthet V. Campos T. Pellegrinet L. Kupczak S. (2020). MYC Instructs and Maintains Pancreatic Adenocarcinoma Phenotype. Cancer Discov. 10 (4), 588–607. 10.1158/2159-8290.cd-19-0435 31941709

[B49] Subramanian A. Tamayo P. Mootha V. K. Mukherjee S. Ebert B. L. Gillette M. A. (2005). Gene Set Enrichment Analysis: a Knowledge-Based Approach for Interpreting Genome-wide Expression Profiles. Proc. Natl. Acad. Sci. 102 (43), 15545–15550. 10.1073/pnas.0506580102 16199517 PMC1239896

[B50] Sun Y. Liu W.-Z. Liu T. Feng X. Yang N. Zhou H.-F. (2015). Signaling Pathway of MAPK/ERK in Cell Proliferation, Differentiation, Migration, Senescence and Apoptosis. J. Receptors Signal Transduction 35 (6), 600–604. 10.3109/10799893.2015.1030412 26096166

[B51] Tan J. Wang W. Song B. Song Y. Meng Z. (2020). Integrative Analysis of Three Novel Competing Endogenous RNA Biomarkers with a Prognostic Value in Lung Adenocarcinoma. Biomed. Res. Int. 2020, 2837906. 10.1155/2020/2837906 32802839 PMC7424383

[B52] Thiery J. P. Acloque H. Huang R. Y. J. Nieto M. A. (2009). Epithelial-Mesenchymal Transitions in Development and Disease. Cell 139 (5), 871–890. 10.1016/j.cell.2009.11.007 19945376

[B53] Van Calster B. Wynants L. Verbeek J. Verbakel J. Christodoulou E. Vickers A. (2018). Reporting and Interpreting Decision Curve Analysis: A Guide for Investigators. Eur. Urol. 74 (6), 796–804. 10.1016/j.eururo.2018.08.038 30241973 PMC6261531

[B54] Wang J. Wang Y. Xu J. Song Q. Shangguan J. Xue M. (2021). Global Analysis of Gene Expression Signature and Diagnostic/prognostic Biomarker Identification of Hepatocellular Carcinoma. Sci. Prog. 104 (3), 003685042110294. 10.1177/00368504211029429 PMC10450782 34315286

[B55] Wolfe A. Singh K. Zhong Y. Drewe P. Rajasekhar V. Sanghvi V. (2014). RNA G-Quadruplexes Cause eIF4A-dependent Oncogene Translation in Cancer. Nature 513 (7516), 65–70. 10.1038/nature13485 25079319 PMC4492470

[B56] Wu Q. Luo X. Terp M. Li Q. Li Y. Shen L. (2021). DDX56 Modulates Post-Transcriptional Wnt Signaling through miRNAs and Is Associated with Early Recurrence in Squamous Cell Lung Carcinoma. Mol. Cancer 20 (1), 108. 10.1186/s12943-021-01403-w 34446021 PMC8393456

[B57] Xia Q. Kong X. Zhang G. Hou X. Qiang H. Zhong R. (2005). Proteomics-based Identification of DEAD-Box Protein 48 as a Novel Autoantigen, a Prospective Serum Marker for Pancreatic Cancer. Biochem. Biophys. Res. Commun. 330 (2), 526–532. 10.1016/j.bbrc.2005.02.181 15796914

[B58] Yang S. He P. Wang J. Schetter A. Tang W. Funamizu N. (2016). A Novel MIF Signaling Pathway Drives the Malignant Character of Pancreatic Cancer by Targeting NR3C2. Cancer Res. 76 (13), 3838–3850. 10.1158/0008-5472.can-15-2841 27197190 PMC4930741

[B59] Yang M.-W. Tao L.-Y. Jiang Y.-S. Yang J.-Y. Huo Y.-M. Liu D.-J. (2020). Perineural Invasion Reprograms the Immune Microenvironment through Cholinergic Signaling in Pancreatic Ductal Adenocarcinoma. Cancer Res. 80 (10), canres.2689.2019–2003. 10.1158/0008-5472.can-19-2689 32098780

[B60] Yu G. Wang L.-G. Han Y. He Q.-Y. (2012). clusterProfiler: an R Package for Comparing Biological Themes Among Gene Clusters. OMICS: A J. Integr. Biol. 16 (5), 284–287. 10.1089/omi.2011.0118 PMC3339379 22455463

[B61] Zeng J. Li X. Liang L. Duan H. Xie S. Wang C. (2022). Phosphorylation of CAP1 Regulates Lung Cancer Proliferation, Migration, and Invasion. J. Cancer Res. Clin. Oncol. 148 (1), 137–153. 10.1007/s00432-021-03819-9 34636991 PMC8752530

[B41] Zhang R. Liu Q. Peng J. Wang M. Gao X. Liao Q. (2019). Pancreatic Cancer-Educated Macrophages Protect Cancer Cells from Complement-Dependent Cytotoxicity by Up-Regulation of CD59. Cell Death Dis. 10 (11), 836. 10.1038/s41419-019-2065-4 31685825 PMC6828776

[B45] Zhang S. Tong Y. Zhang X. Zhang Y. Xu X. Xiao A. (2019). A Novel and Validated Nomogram to Predict Overall Survival for Gastric Neuroendocrine Neoplasms. J. Cancer 10 (24), 5944–5954. 10.7150/jca.35785 31762804 PMC6856574

[B62] Zhang X. Milton C. Humbert P. Harvey K. (2009). Transcriptional Output of the Salvador/warts/hippo Pathway Is Controlled in Distinct Fashions in *Drosophila melanogaster* and Mammalian Cell Lines. Cancer Res. 69 (15), 6033–6041. 10.1158/0008-5472.can-08-4592 19584286

[B63] Zhang Y. Zhou M. Wei H. Zhou H. He J. Lu Y. (2017). Furin Promotes Epithelial-Mesenchymal Transition in Pancreatic Cancer Cells via Hippo-YAP Pathway. Int. J. Oncol. 50 (4), 1352–1362. 10.3892/ijo.2017.3896 28259973

[B64] Zhang R. Liu Q. Liao Q. Zhao Y. (2018). CD59: a Promising Target for Tumor Immunotherapy. Future Oncol. 14 (8), 781–791. 10.2217/fon-2017-0498 29521526

[B65] Zhao M. Li S. Zhou L. Shen Q. Zhu H. Zhu X. (2018). Prognostic Values of Excision Repair Cross-Complementing Genes mRNA Expression in Ovarian Cancer Patients. Life Sci. 194, 34–39. 10.1016/j.lfs.2017.12.018 29247747

[B66] Zhao T. Jin F. Xiao D. Wang H. Huang C. Wang X. (2020). IL-37/STAT3/HIF-1alpha Negative Feedback Signaling Drives Gemcitabine Resistance in Pancreatic Cancer. Theranostics 10 (9), 4088–4100. 10.7150/thno.42416 32226541 PMC7086367

