# Supplementary material for: Identification of DDX31 as a Potential Oncogene of Invasive Metastasis and Proliferation in PDAC
Source: Front Cell Dev Biol. 2022 Feb 14;10:762372. doi: 10.3389/fcell.2022.762372 (PMC8883474; doi:10.3389/fcell.2022.762372)

Figure7：

IHC images

Classical case- sample 9：

Tumor Part Low expression(score=1)

Tumor Part High expression(score=3)

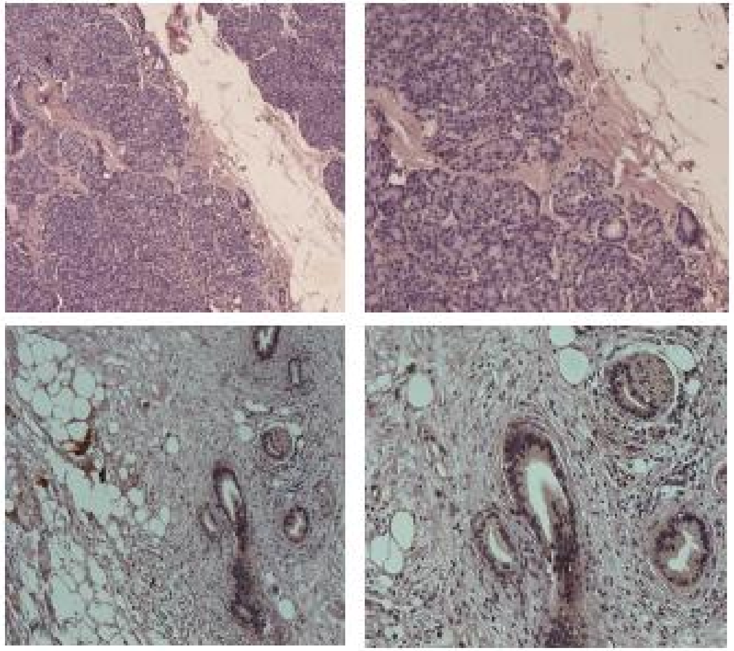

Classical case Sample1

Tumor Part Low expression(score=1)

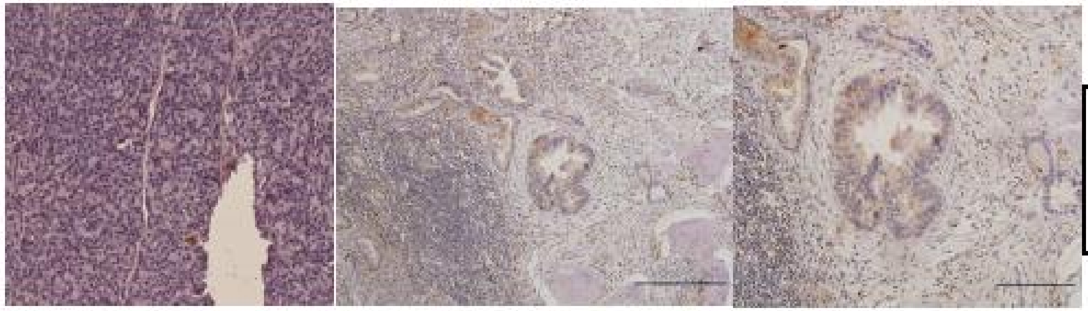

Tumor Part moderate expression(score=2)

Classical Sample3

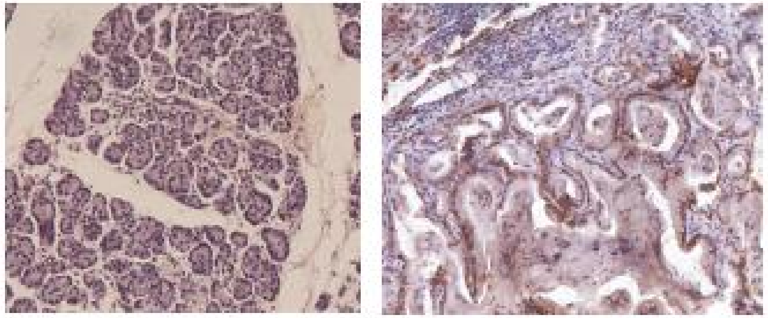

Tumor Part High expression(score=3)

Classical Sample5

Normal Part Low Expression(score=1)

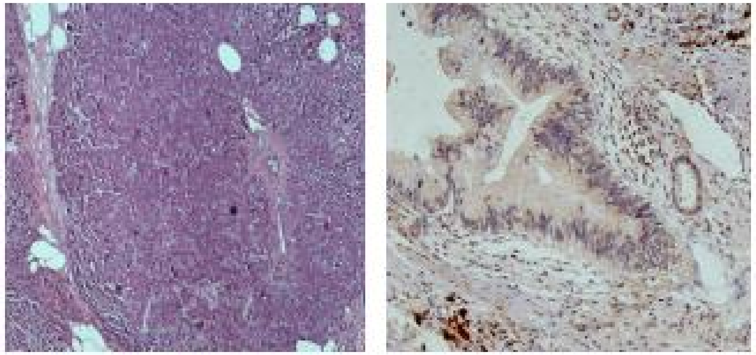

Tumor Part Moderate expression(score=2)

Sample6

Tumor Part Low expression(score=1)

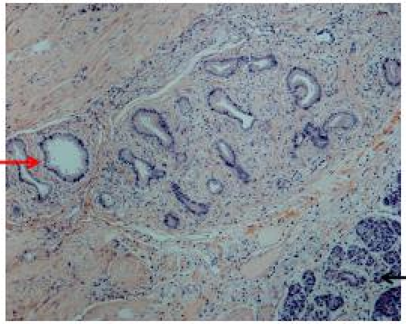

Normal Part Low Expression(score=1)

Sample7

Normal Part moderate expression(score=2)

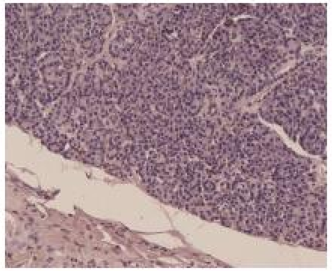

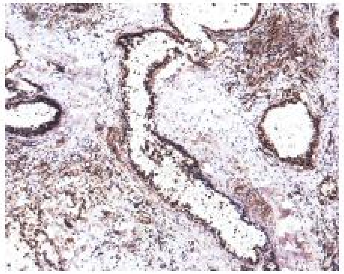

Tumor Part High expression(score=3)

Sample8

Normal Part Low expression(score=1)

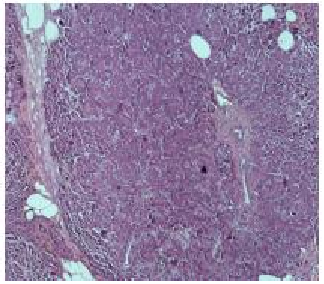

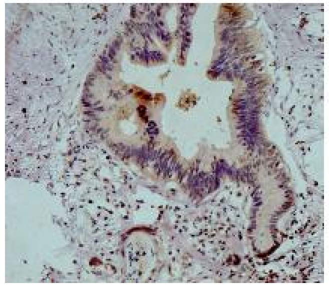

Tumor Part Moderate expression(score=2)

Sample 10

Normal Part Low expression(score=1)

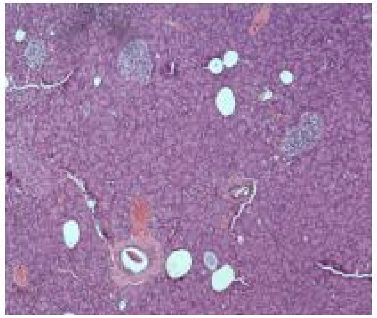

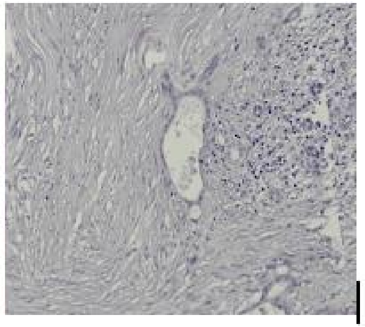

Tumor Part High expression(score=3)

Sample11

Normal Part Low Expression(score=1)

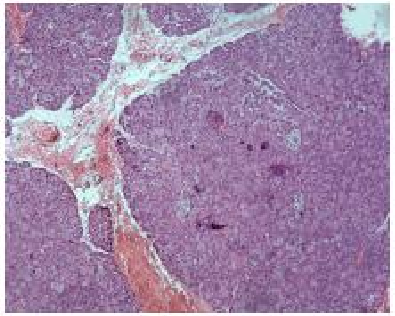

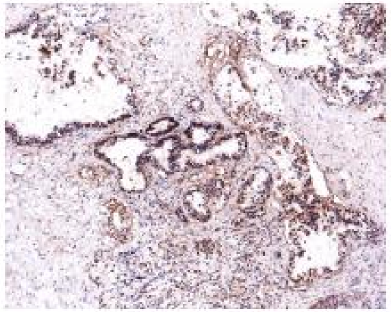

Tumor Part Low expression(score=1)

Sample15

Tumor Part Low expression(score=1)

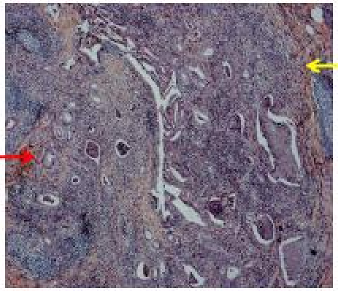

Normal Part Moderate Expression(score=2)

Sample14

Normal Part Low Expression(score=1)

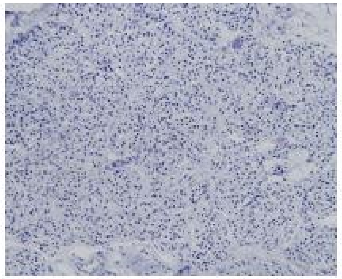

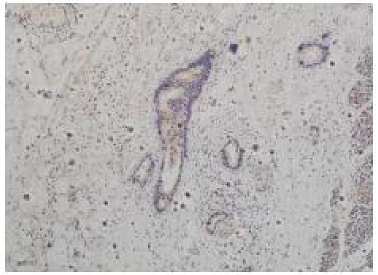

Tumor Part Moderate expression(score=2)

Sample28

Normal Part Low Expression(score=1)

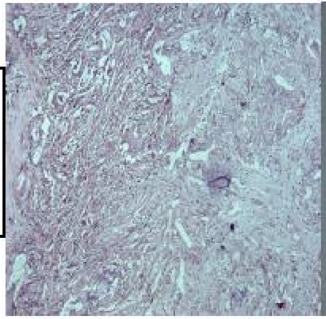

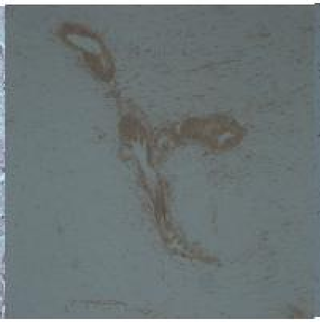

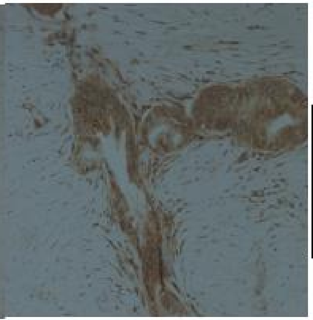

Tumor Part High expression(score=3)

Sample35

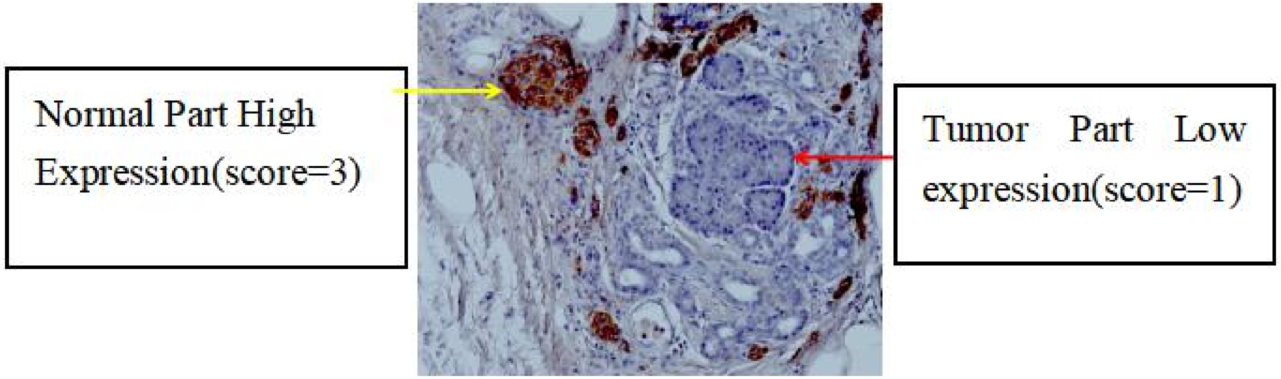

Sample79

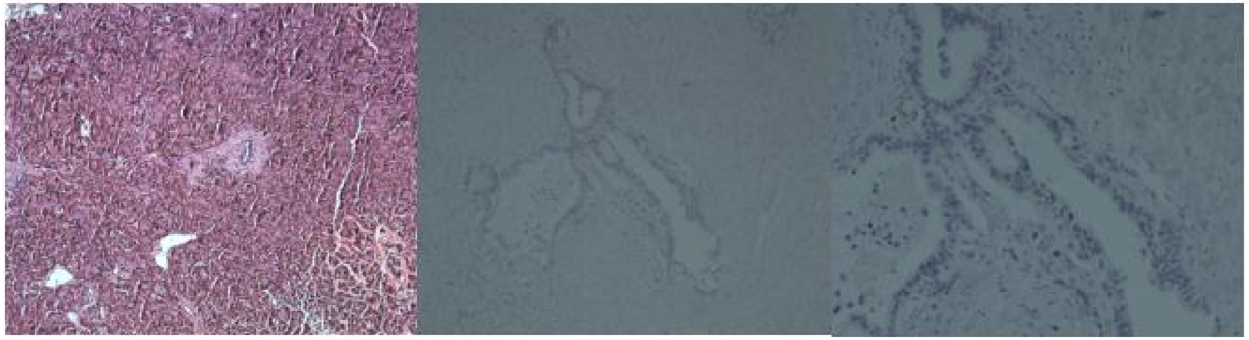

IHC scores

Table format: Grouped	Group A	Group B
	N	T
sample1	1	2
sample2	1	3
sample3	2	3
sample4	1	3
sample5	1	2
sample6	1	1
sample7	2	3
sample8	1	2
sample9	1	3
sample10	1	1
sample11	1	3
sample12	3	1
sample13	1	3
sample14	1	2
sample15	2	1
sample16	1	3
sample17	1	2

Table format: Grouped	Group A	Group B
	N	T
sample18	1	2
sample19	1	3
sample20	2	3
sample21	1	2
sample22	1	1
sample23	1	2
sample24	1	2
sample25	3	3
sample26	1	2
sample27	1	1
sample28	1	3
sample29	1	1
sample30	2	3
sample31	1	1
sample32	1	2
sample33	1	2
sample34	1	2

Table format: Grouped	Group A	Group B
	N	T
sample35	3	1
sample36	2	3
sample37	1	3
sample38	3	3
sample39	1	3
sample40	3	2
sample41	1	3
sample42	1	1
sample43	2	3
sample44	2	1
sample45	1	1
sample46	1	2
sample47	2	1
sample48	1	3
sample49	1	2
sample50	3	3
sample51	1	3

Table format: Grouped	Group A	Group B
	N	T
sample52	1	3
sample53	1	2
sample54	1	1
sample55	1	3
sample56	3	3
sample57	1	1
sample58	1	1
sample59	1	3
sample60	1	1
sample61	1	2
sample62	1	1
sample63	3	1
sample64	1	3
sample65	1	3
sample66	1	2
sample67	1	3
sample68	2	3

Table format: Grouped	Group A	Group B
	N	T
sample69	1	1
sample70	1	1
sample71	1	3
sample72	1	1
sample73	1	1
sample74	3	3
sample75	1	3
sample76	2	3
sample77	1	3
sample78	1	3
sample79	1	1
sample80	2	3
sample81	1	3
sample82	1	2
sample83	1	3
sample84	2	2
sample85	3	1

Table format: Grouped	Group A	Group B
	N	T
sample72	1	1
sample73	1	1
sample74	3	3
sample75	1	3
sample76	2	3
sample77	1	3
sample78	1	3
sample79	1	1
sample80	2	3
sample81	1	3
sample82	1	2
sample83	1	3
sample84	2	2
sample85	3	1
sample86	1	2

Figure7 c

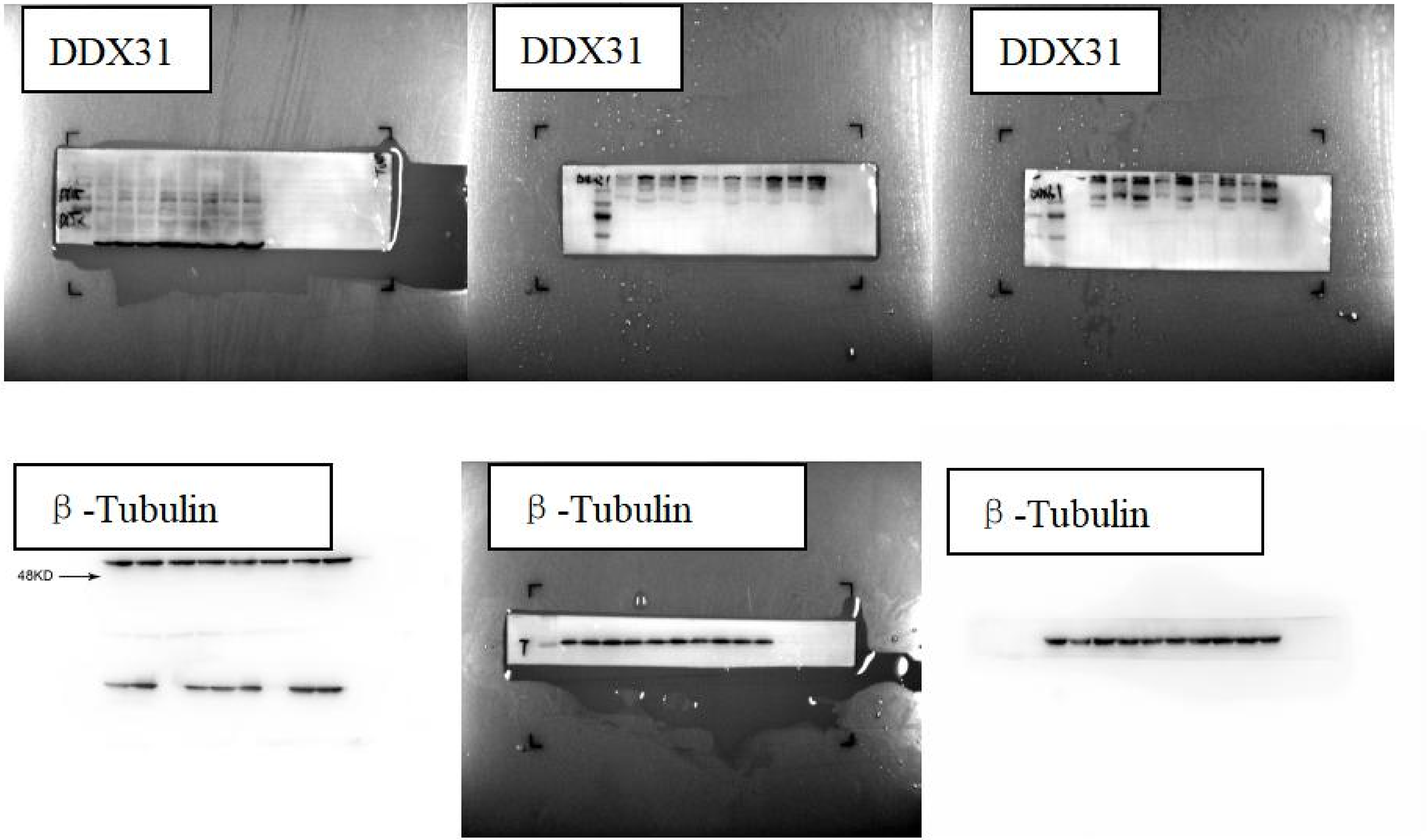

Figure8 a

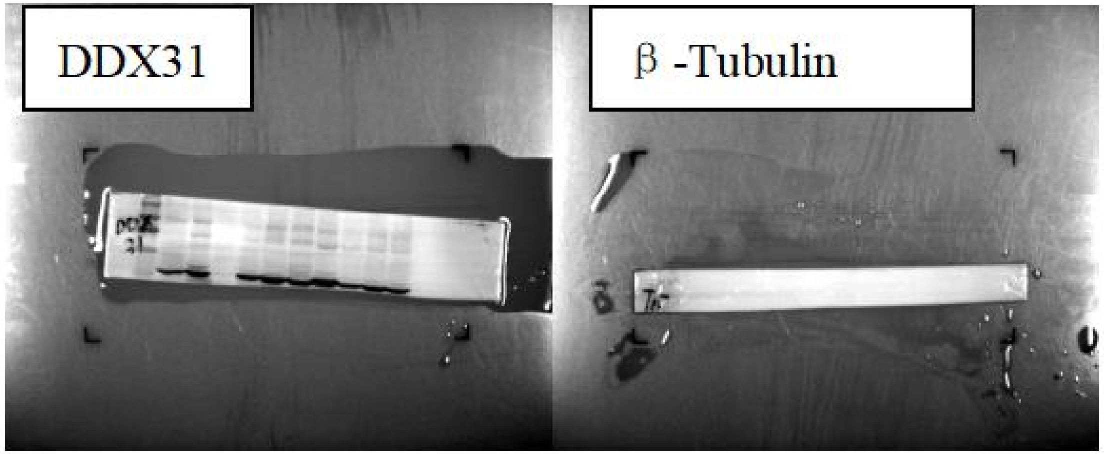

Figure8 c

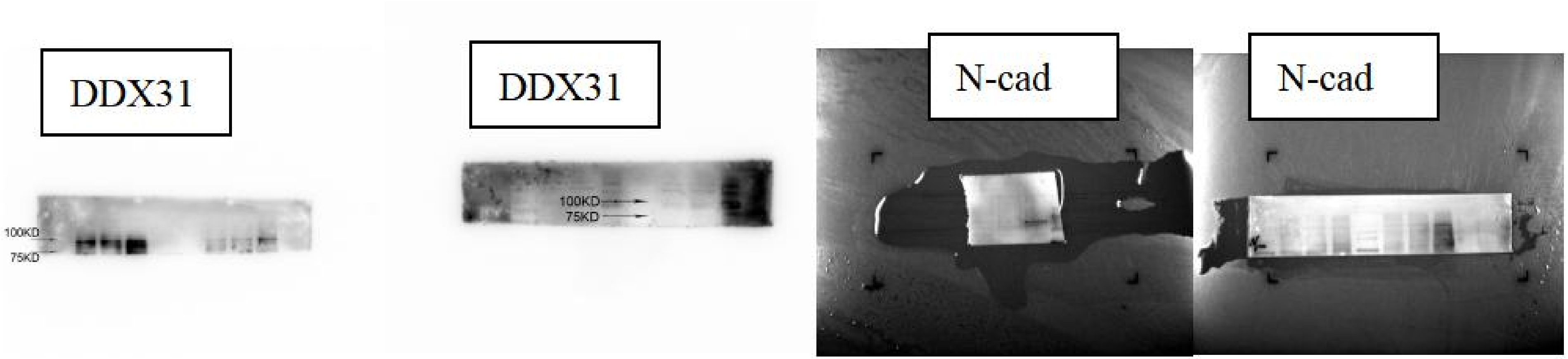

Snail

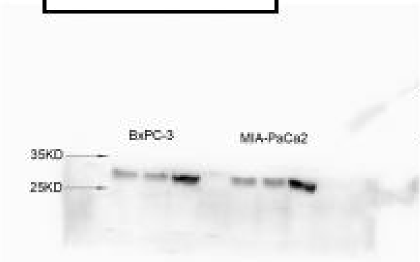

ZEB1

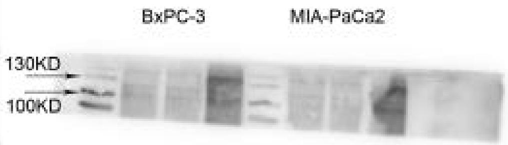

Ki-67

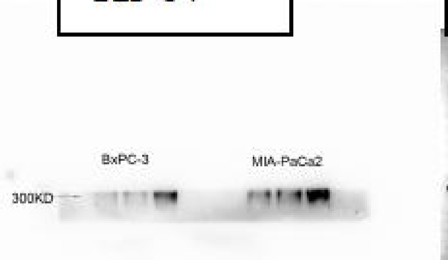

Ki-67 merge

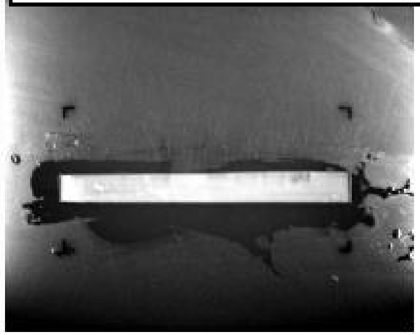

Ki-67 merge

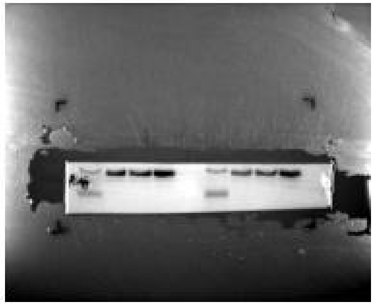

Ki-67

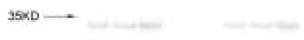

β -Tubulin

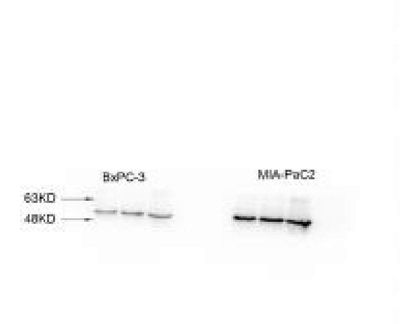

Figure8 c Sh-DDX31

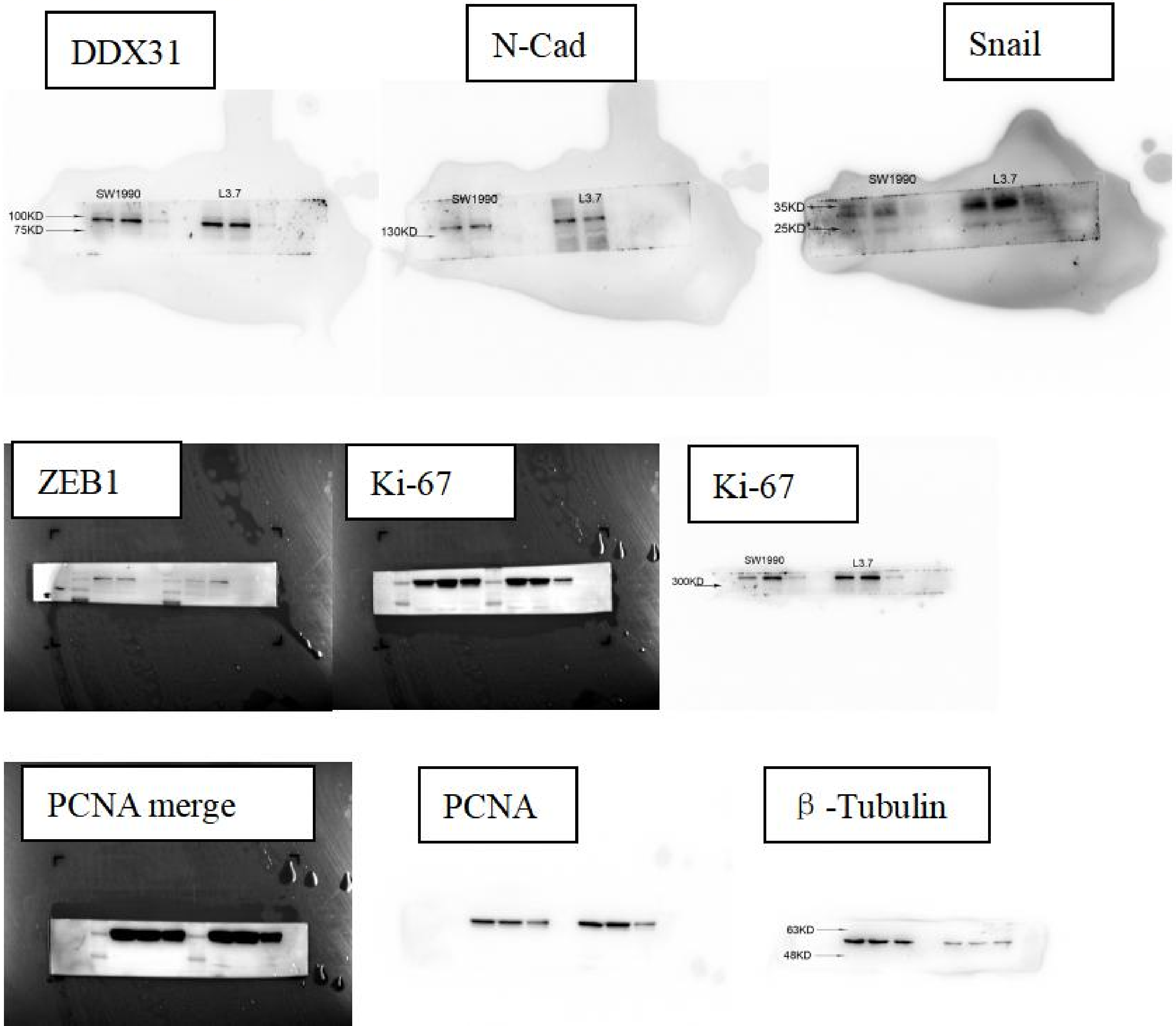

Figure8 d

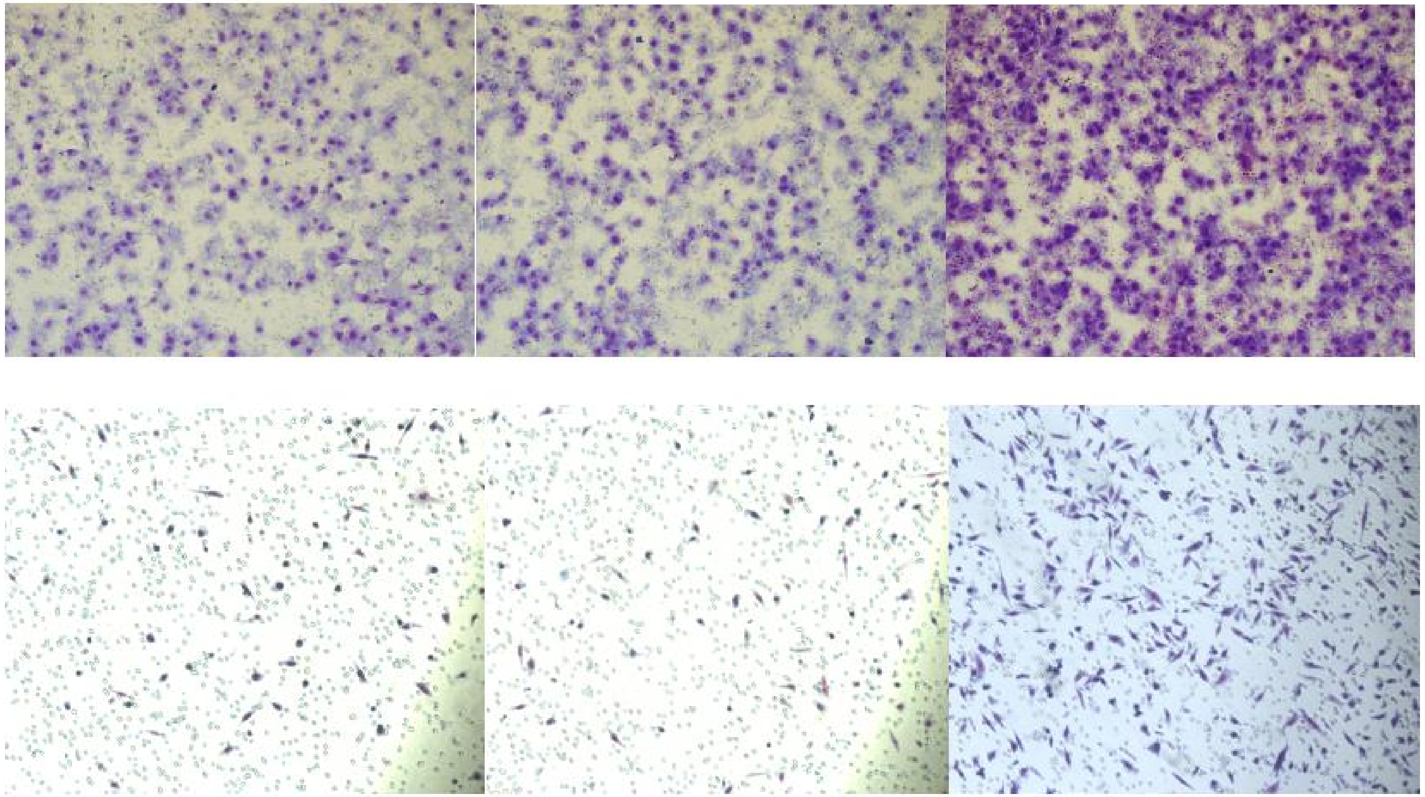

Data Tables: **BxPC-3**

	X	Group A			Group B			Group C		
	X Title	WT			pCDH-VECTOR			pCDH-DDX31		
	X	A:Y1	A:Y2	A:Y3	B:Y1	B:Y2	B:Y3	C:Y1	C:Y2	C:Y3
1 Title		175	234	267	214	168	259	445	386	475
2 Title										

Data Tables: **MIA-PaCa2**

Table format: Grouped	Group A			Group B			Group C		
	WT			pCDH-VECTOR			pCDH-DDX31		
	A:1	A:2	A:3	B:1	B:2	B:3	C:1	C:2	C:3
1 Title	75	64	71	54	62	78	258	210	326
2									

Figure8 e

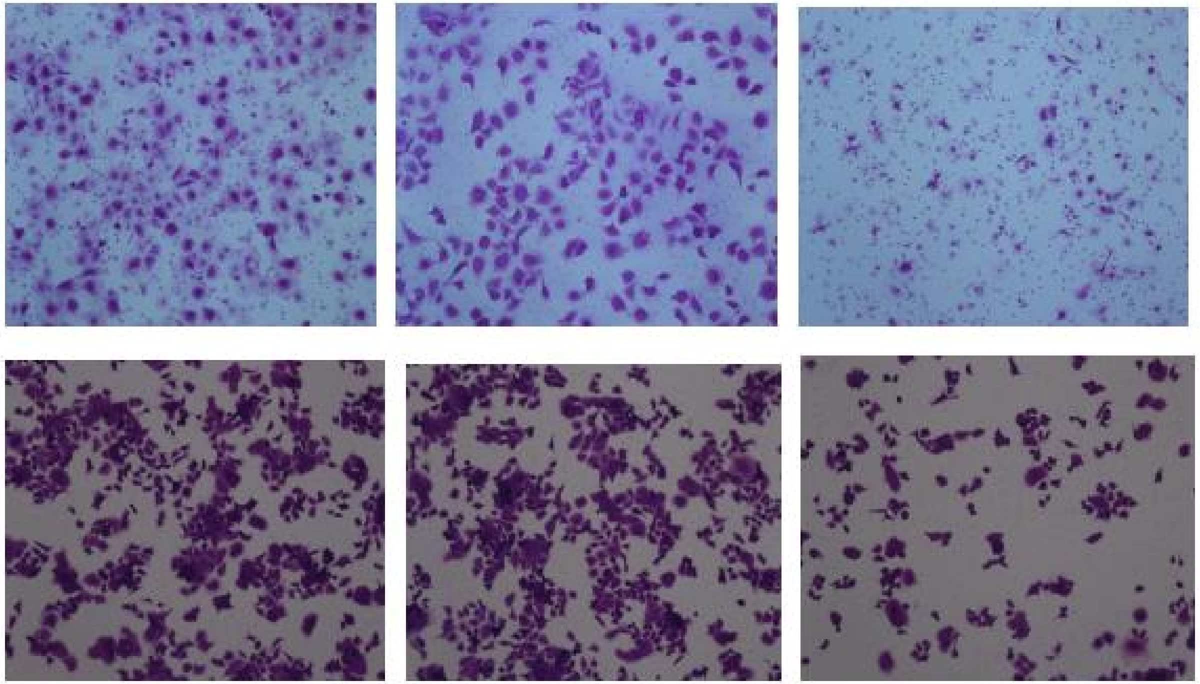

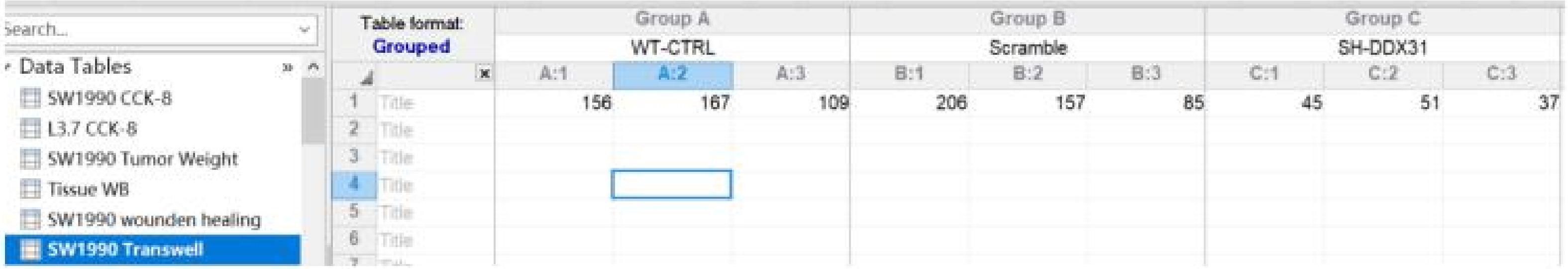

Data Tables
- SW1990 CCK-8
- L3.7 CCK-8
- SW1990 Tumor Weight
- Tissue WB
- SW1990 wounden healing
- SW1990 Transwell

Table format: Grouped	Group A WT-CTRL			Group B Scramble			Group C SH-DDX31		
	A:1	A:2	A:3	B:1	B:2	B:3	C:1	C:2	C:3
1 Title	156	167	109	206	157	85	45	51	37
2 Title									
3 Title									
4 Title									
5 Title									
6 Title									

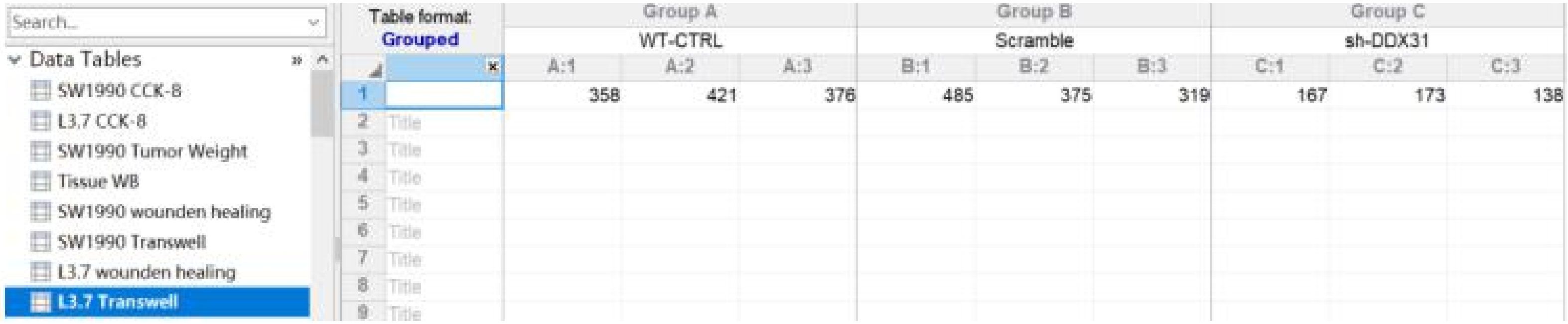

Data Tables
- SW1990 CCK-8
- L3.7 CCK-8
- SW1990 Tumor Weight
- Tissue WB
- SW1990 wounden healing
- SW1990 Transwell
- L3.7 wounden healing
- L3.7 Transwell

Table format: Grouped	Group A WT-CTRL			Group B Scramble			Group C sh-DDX31		
	A:1	A:2	A:3	B:1	B:2	B:3	C:1	C:2	C:3
1	358	421	376	485	375	319	167	173	138
2 Title									
3 Title									
4 Title									
5 Title									
6 Title									
7 Title									
8 Title									
9 Title									

Figure8 f

BxPC-3

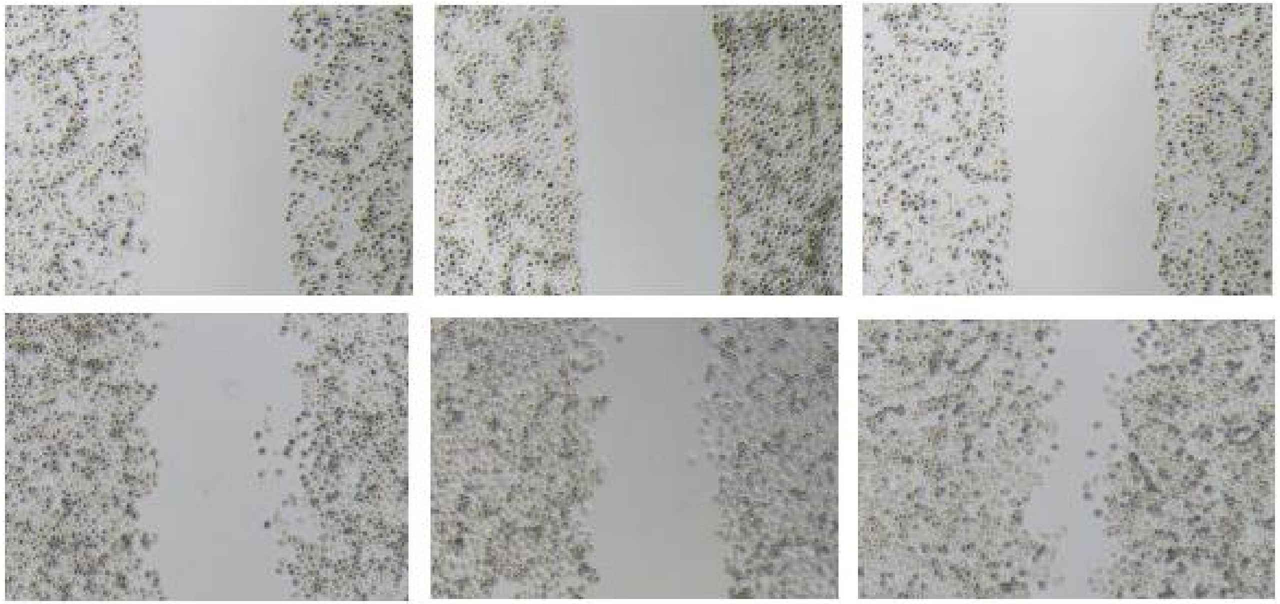

MIA-PaCa2

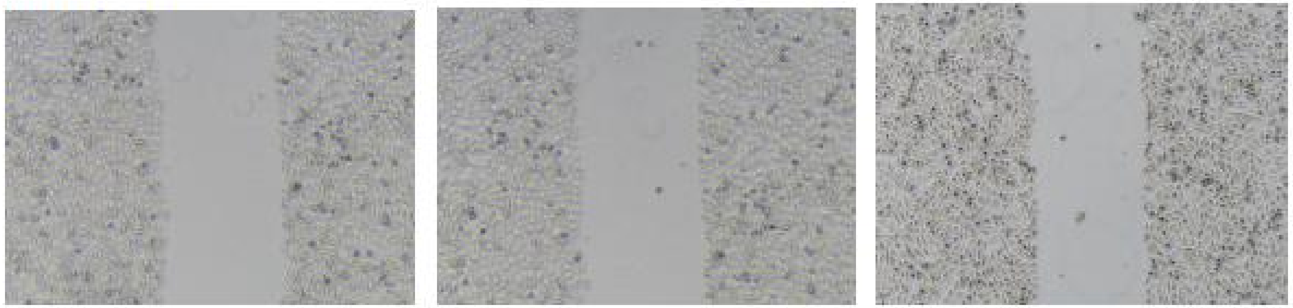

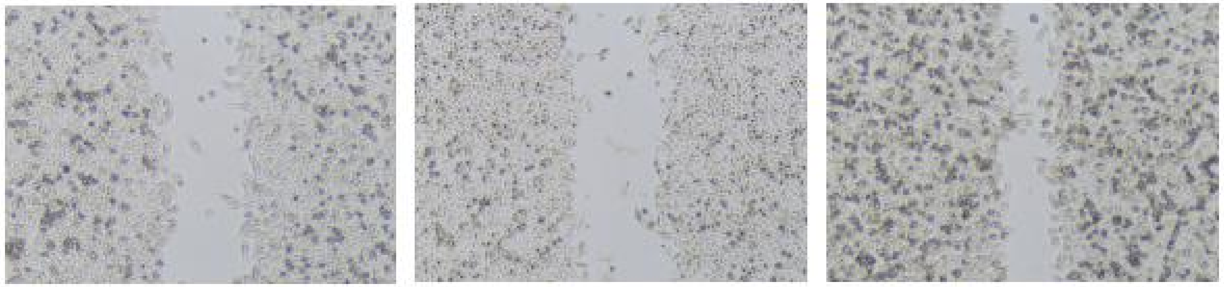

Search...

Data Tables

MIA-PaCa2 Wounden Healing

BxPC-3 Wounden Healing

	X	Group A			Group B			Group C		
	X Title	WT			pCDH-VECTOR			pCDH-DDX31		
	X	A:Y1	A:Y2	A:Y3	B:Y1	B:Y2	B:Y3	C:Y1	C:Y2	C:Y3
1 Title		77	73	80	72	84	74	47	52	56
2 Title										

Data Tables

MIA-PaCa2 Wounden Healing

BxPC-3 Wounden Healing

	XY									
	X Title	WT			pCDH-VECTOR			pCDH-DDX31		
	X	A:Y1	A:Y2	A:Y3	B:Y1	B:Y2	B:Y3	C:Y1	C:Y2	C:Y3
1 Area of the		66	75	72	72	74	68	26	28	43
2 Title										

SW1990

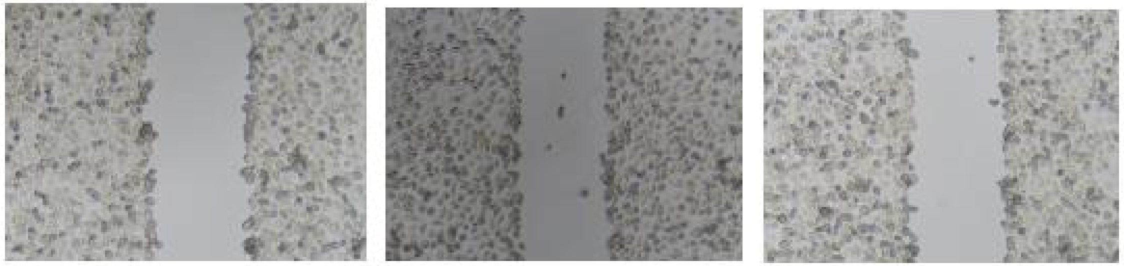

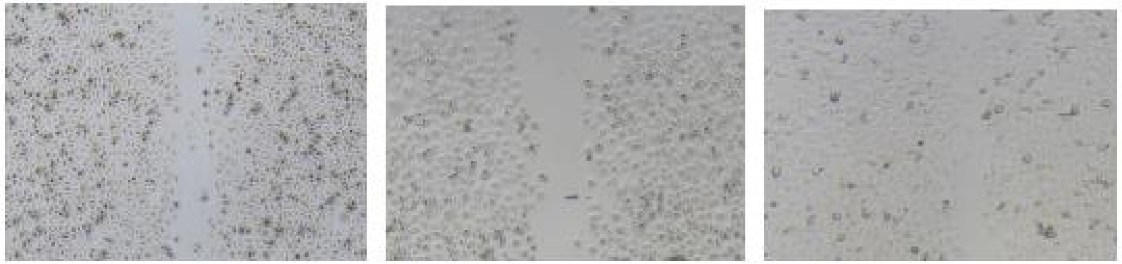

L3.7

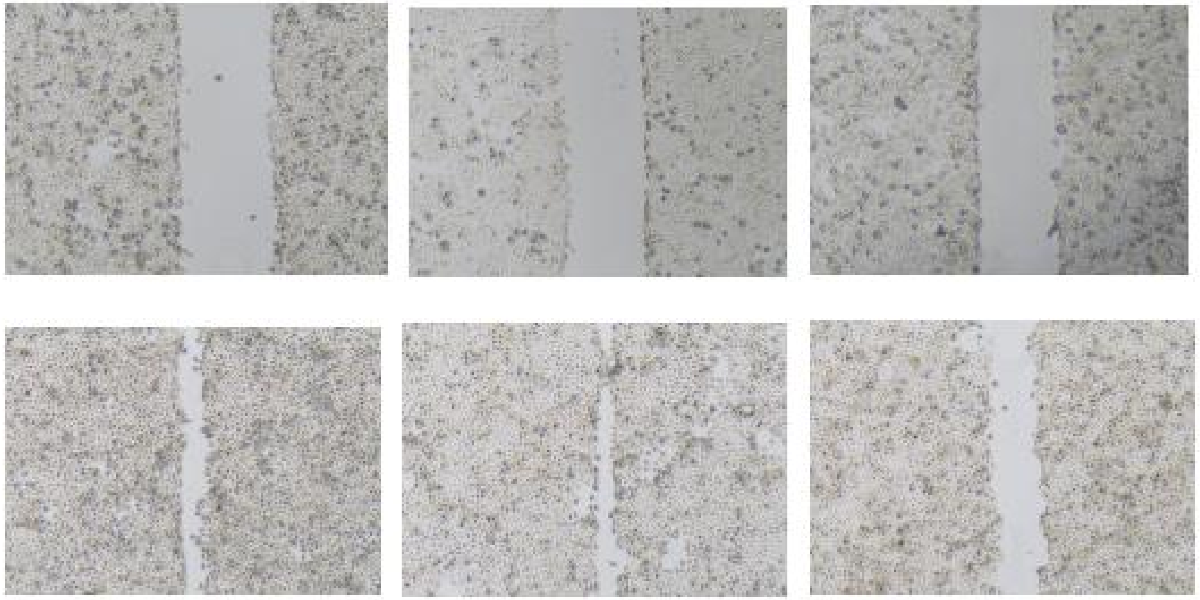

Search...

Data Tables
- SW1990 CCK-8
- L3.7 CCK-8
- SW1990 Tumor Weight
- Tissue WB
- **SW1990 wounden healing**

Table format: **Grouped**

	Group A<br>WT-CTRL			Group B<br>VECTOR			Group C<br>SH-DDX31		
	A:1	A:2	A:3	B:1	B:2	B:3	C:1	C:2	C:3
1 Title	5.6	7.3	9.4	8.7	6.3	5.6	26.3	15.3	38.7
2 Title									
3 Title									
4 Title									
5 Title									

Search...

Data Tables
- SW1990 CCK-8
- L3.7 CCK-8
- SW1990 Tumor Weight
- Tissue WB
- **SW1990 wounden healing**

Table format: **Grouped**

	Group A<br>WT-CTRL			Group B<br>VECTOR			Group C<br>SH-DDX31		
	A:1	A:2	A:3	B:1	B:2	B:3	C:1	C:2	C:3
1 Title	5.6	7.3	9.4	8.7	6.3	5.6	26.3	15.3	38.7
2 Title									
3 Title									
4 Title									
5 Title									

Figure9 a

Bx PC-3

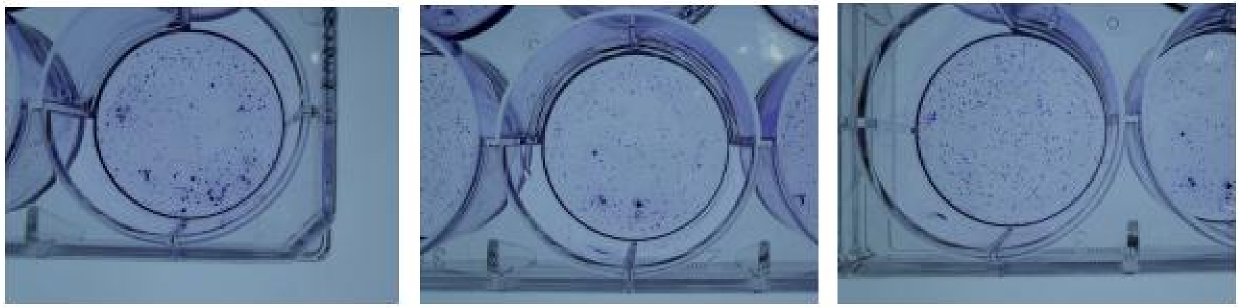

MIA-PaCa2

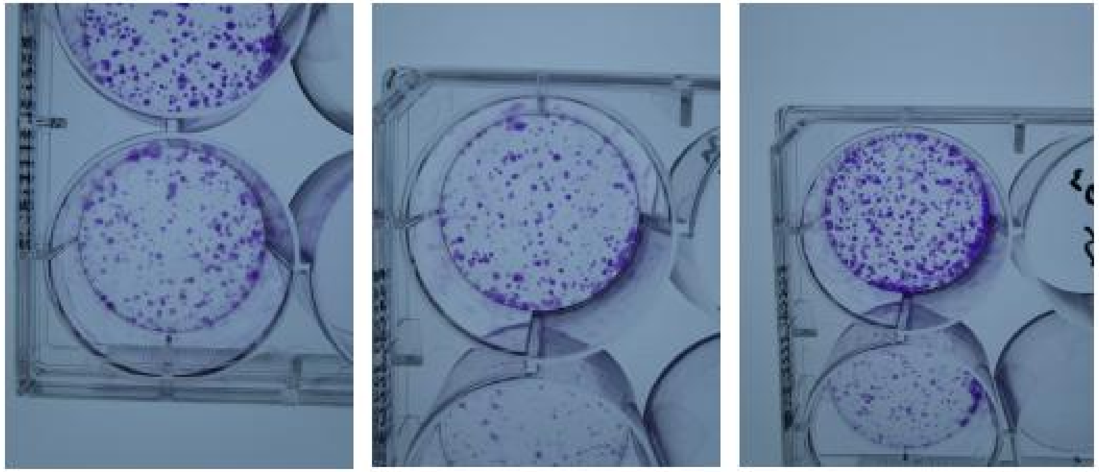

Search...

Data Tables

MIA-PaCa2 clone formation

BxPC-3 clone formation

	X	Group A			Group B			Group C		
	X Title	WT-CTRL			PCDH-VECTOR			PCDH-DDX31		
	X	A:Y1	A:Y2	A:Y3	B:Y1	B:Y2	B:Y3	C:Y1	C:Y2	C:Y3
1	Title	115	74	58	121	71	59	290	320	276
2	Title									

Search...

Data Tables

MIA-PaCa2 clone formation

BxPC-3 clone formation

	X	Group A			Group B			Group C		
	X Title	WT-CTRL			PCDH-VECTOR			PCDH-DDX31		
	X	A:Y1	A:Y2	A:Y3	B:Y1	B:Y2	B:Y3	C:Y1	C:Y2	C:Y3
1	Title	89	75	107	110	94	83	382	485	362
2	Title									

SW1990

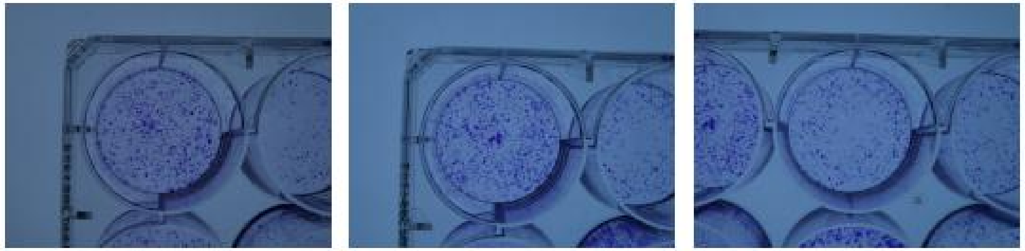

L3.7

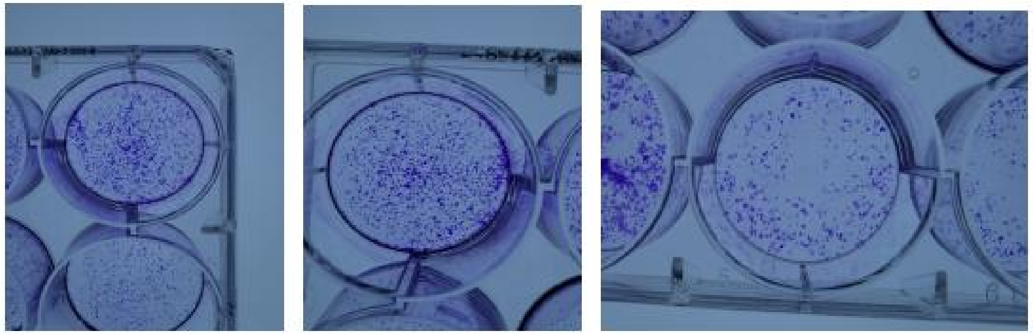

Search...

Data Tables
- SW1990 CCK-8
- L3.7 CCK-8
- SW1990 Tumor Weight
- Tissue WB
- SW1990 wounden healing
- SW1990 Transwell
- L3.7 wounden healing
- L3.7 Transwell
- SW1990 Clone formation

Table format: Grouped	Group A			Group B			Group C		
	WT-CTRL			Scramble			sh-DDX31		
	A:1	A:2	A:3	B:1	B:2	B:3	C:1	C:2	C:3
1 Title	376	390	227	365	278	295	113	95	87

Search...

Data Tables
- SW1990 CCK-8
- L3.7 CCK-8
- SW1990 Tumor Weight
- Tissue WB
- SW1990 wounden healing
- SW1990 Transwell
- L3.7 wounden healing
- L3.7 Transwell
- SW1990 Clone formation
- L3.7 clone formation

Table format: Grouped	Group A			Group B			Group C		
	WT-CTRL			Scramble			sh-DDX31		
	A:1	A:2	A:3	B:1	B:2	B:3	C:1	C:2	C:3
1 Title	337	287	354	423.0	376.0	381.0	125.0	153.0	229.0

Figure9 c

BxPC-3

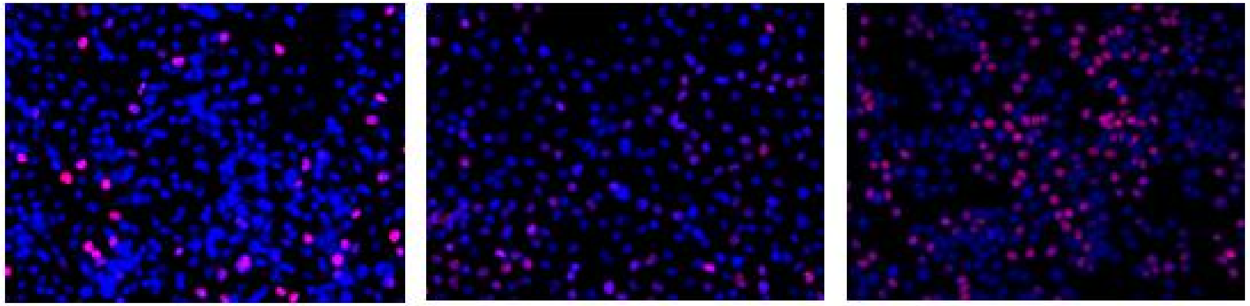

MIA-PaCa2

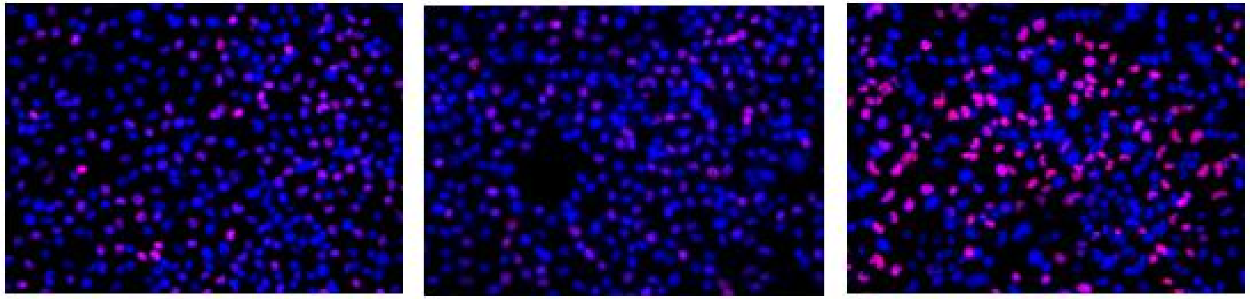

Data Tables: **BxPC-3 EdU Stain**, MIA-PaCa2 EdU Stain

	X	Group A			Group B			Group C		
	X Title	WT			pCDH-VECTOR			pCDH-DDX31		
	X	A:Y1	A:Y2	A:Y3	B:Y1	B:Y2	B:Y3	C:Y1	C:Y2	C:Y3
1	Title	21	38	27	25	21	31	43	52	44
2	Title									

Data Tables: BxPC-3 EdU Stain, **MIA-PaCa2 EdU Stain**

Table format: Grouped		Group A			Group B			Group C		
		WT			pCDH-VECTOR			pCDH-DDX31		
		A:1	A:2	A:3	B:1	B:2	B:3	C:1	C:2	C:3
1	Title	33	47	42	36	38	45	68	73	65
2	Title									

SW1990

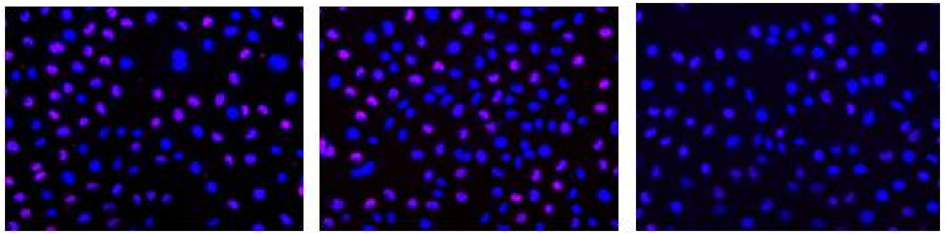

L3.7

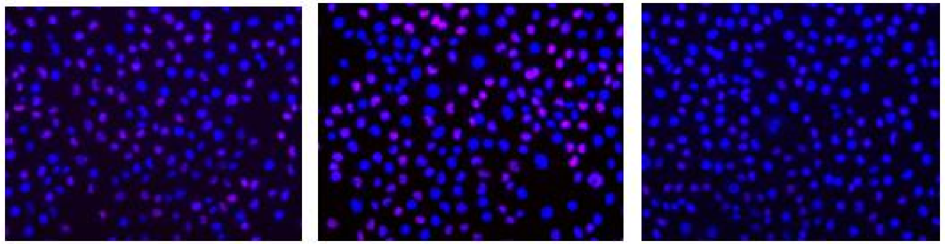

Search...

Data Tables

- SW1990 CCK-8
- L3.7 CCK-8
- SW1990 Tumor Weight
- Tissue WB
- SW1990 wounden healing
- SW1990 Transwell
- L3.7 wounden healing
- L3.7 Transwell
- SW1990 Clone formation
- L3.7 clone formation
- **SW EDU**

Table format: Grouped	Group A WT-CTRL			Group B Scramble			Group C sh-DDX31		
	A:1	A:2	A:3	B:1	B:2	B:3	C:1	C:2	C:3
1 Title	63.0	56.3	51.6	54.6	65.3	43.8	26.7	37.7	30.4
2 Title									
3 Title									
4 Title									
5 Title									
6 Title									
7 Title									
8 Title									
9 Title									
10 Title									
11 Title									
12 Title									

Search...

Data Tables

- SW1990 CCK-8
- L3.7 CCK-8
- SW1990 Tumor Weight
- Tissue WB
- SW1990 wounden healing
- SW1990 Transwell
- L3.7 wounden healing
- L3.7 Transwell
- SW1990 Clone formation
- L3.7 clone formation
- SW EDU
- **L3.7 EDU**

Table format: Grouped	Group A WT-CTRL			Group B Scramble			Group C sh-DDX31		
	A:1	A:2	A:3	B:1	B:2	B:3	C:1	C:2	C:3
1 Title	68.6	61.4	57.8	54.2	78.1	59.8	34.5	28.9	38.8
2 Title									
3 Title									
4 Title									
5 Title									
6 Title									
7 Title									
8 Title									
9 Title									
10 Title									
11 Title									
12 Title									
13 Title									

Vivo experiment

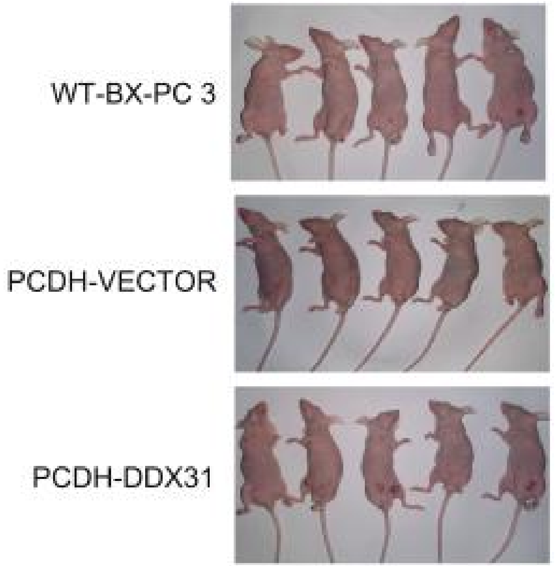

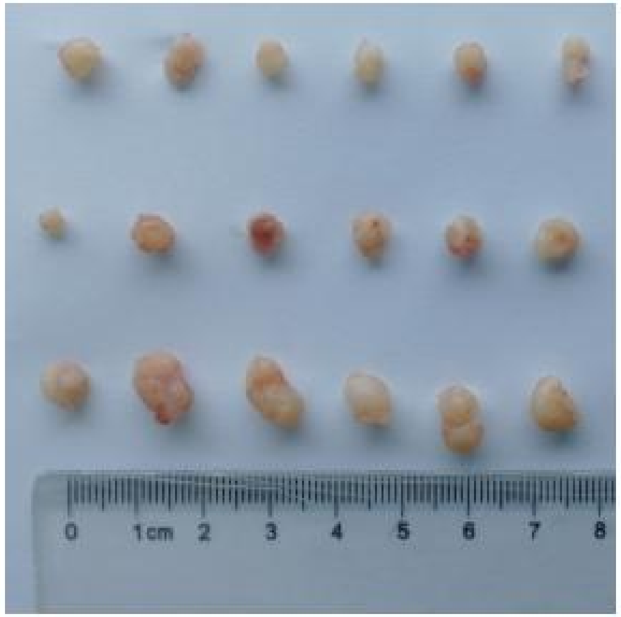

Supplement: Supplementary file 1 [file DataSheet2.PDF]
