# Supplementary figures and images for: Identification of DDX31 as a Potential Oncogene of Invasive Metastasis and Proliferation in PDAC
Source: Front Cell Dev Biol. 2022 Feb 14;10:762372. doi: 10.3389/fcell.2022.762372 (PMC8883474; doi:10.3389/fcell.2022.762372)

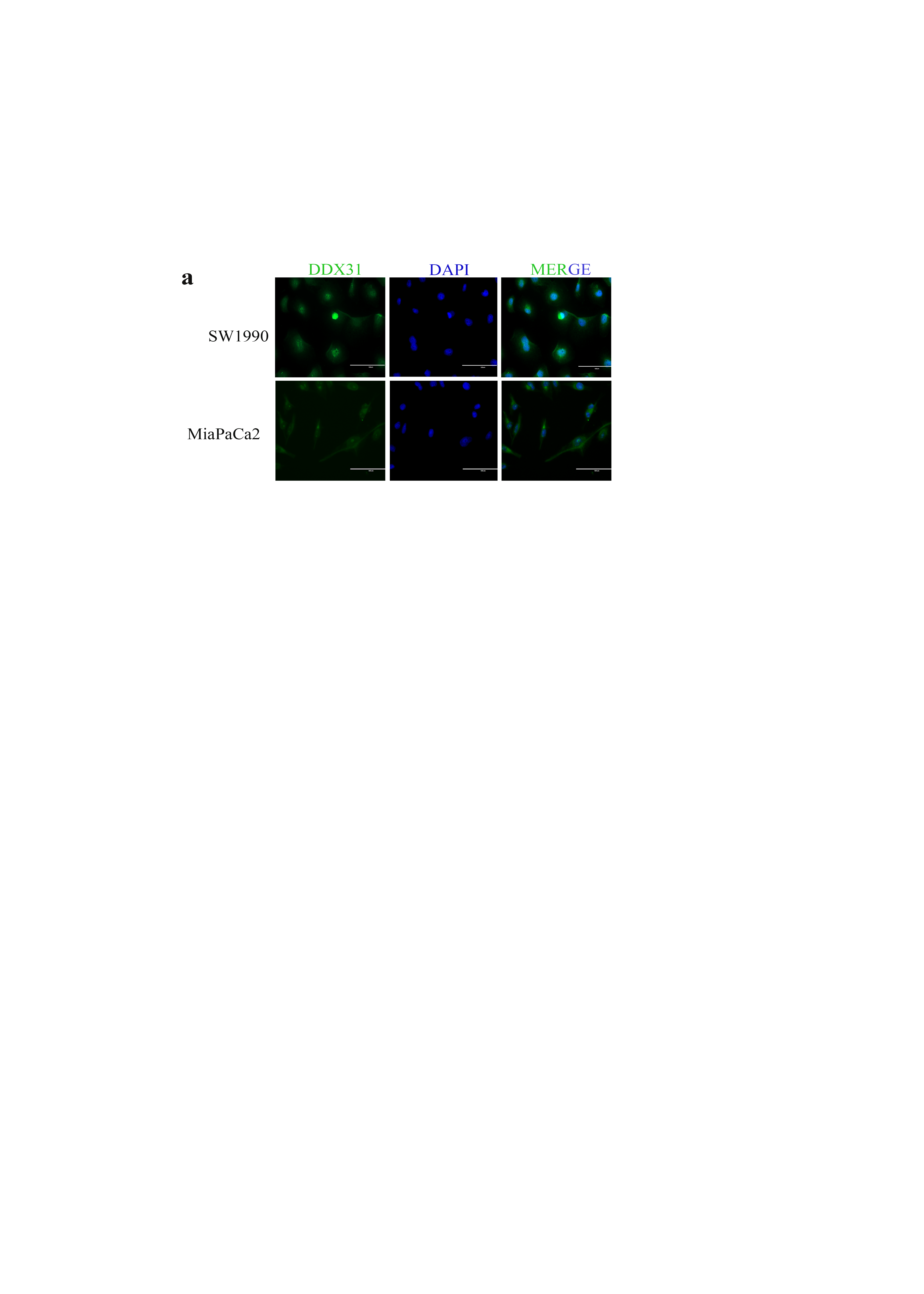

Supplement: Supplementary file 5 [file Image6.TIF]

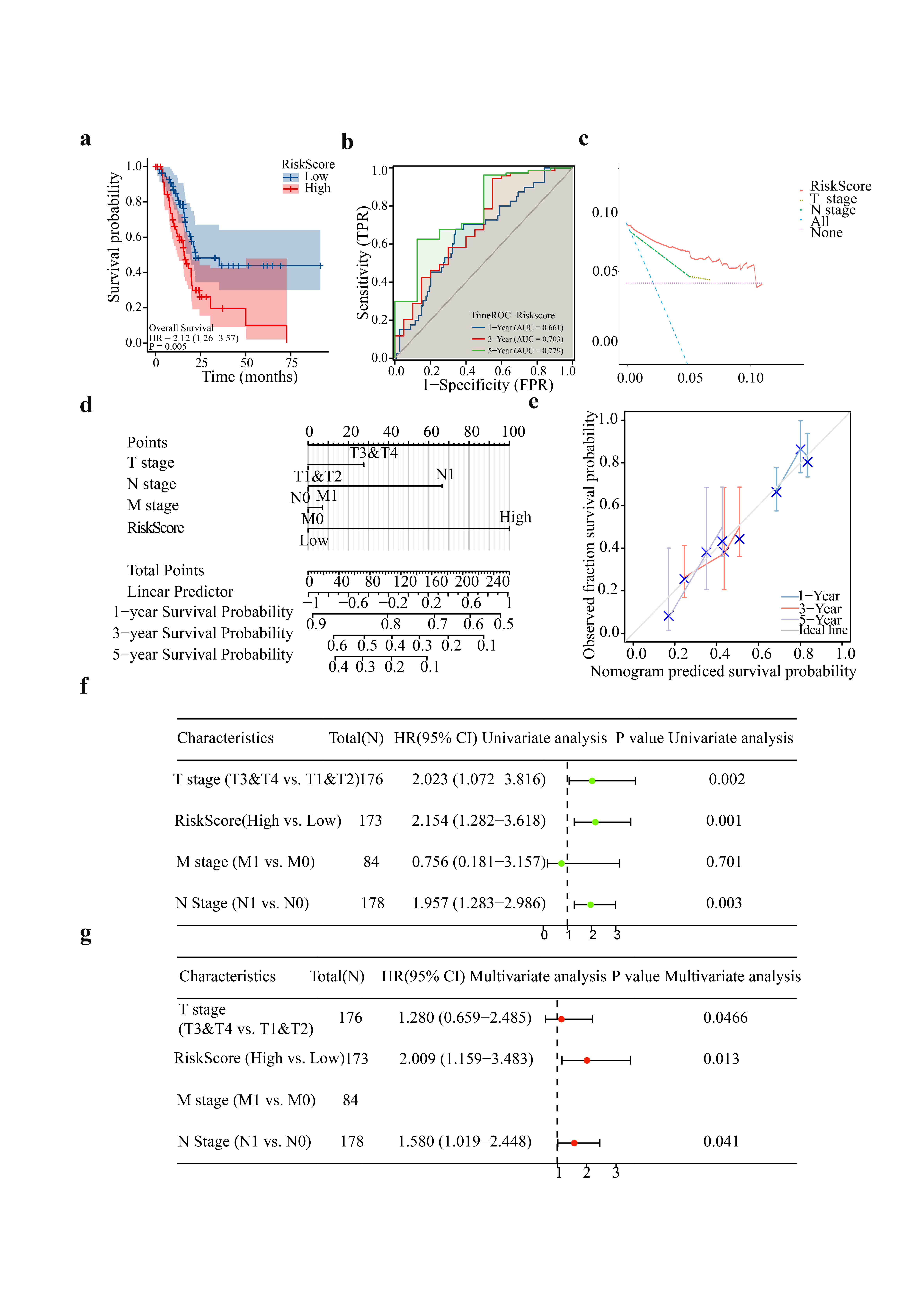

Supplement: Supplementary file 6 [file Image3.TIF]

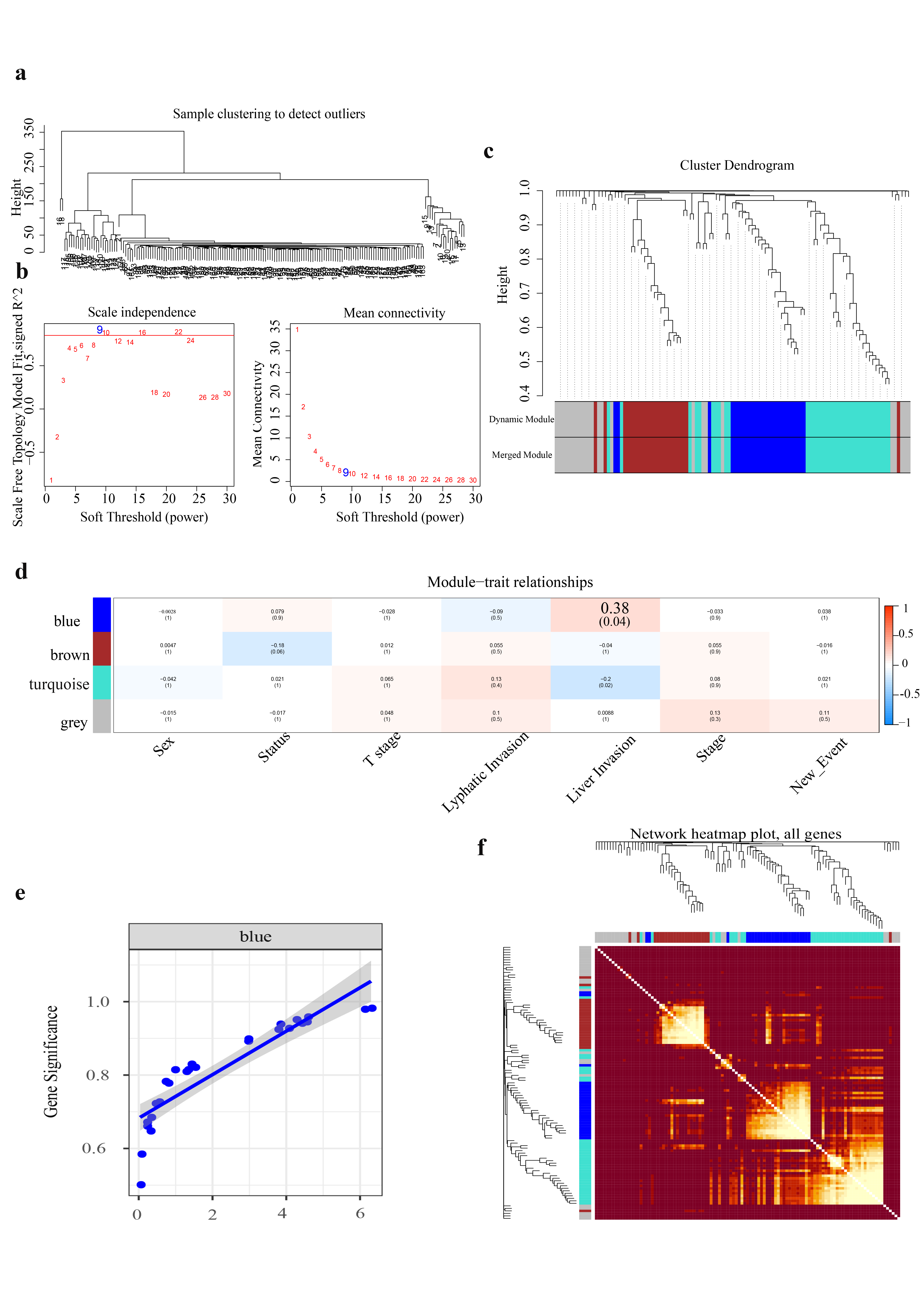

Supplement: Supplementary file 7 [file Image4.TIF]

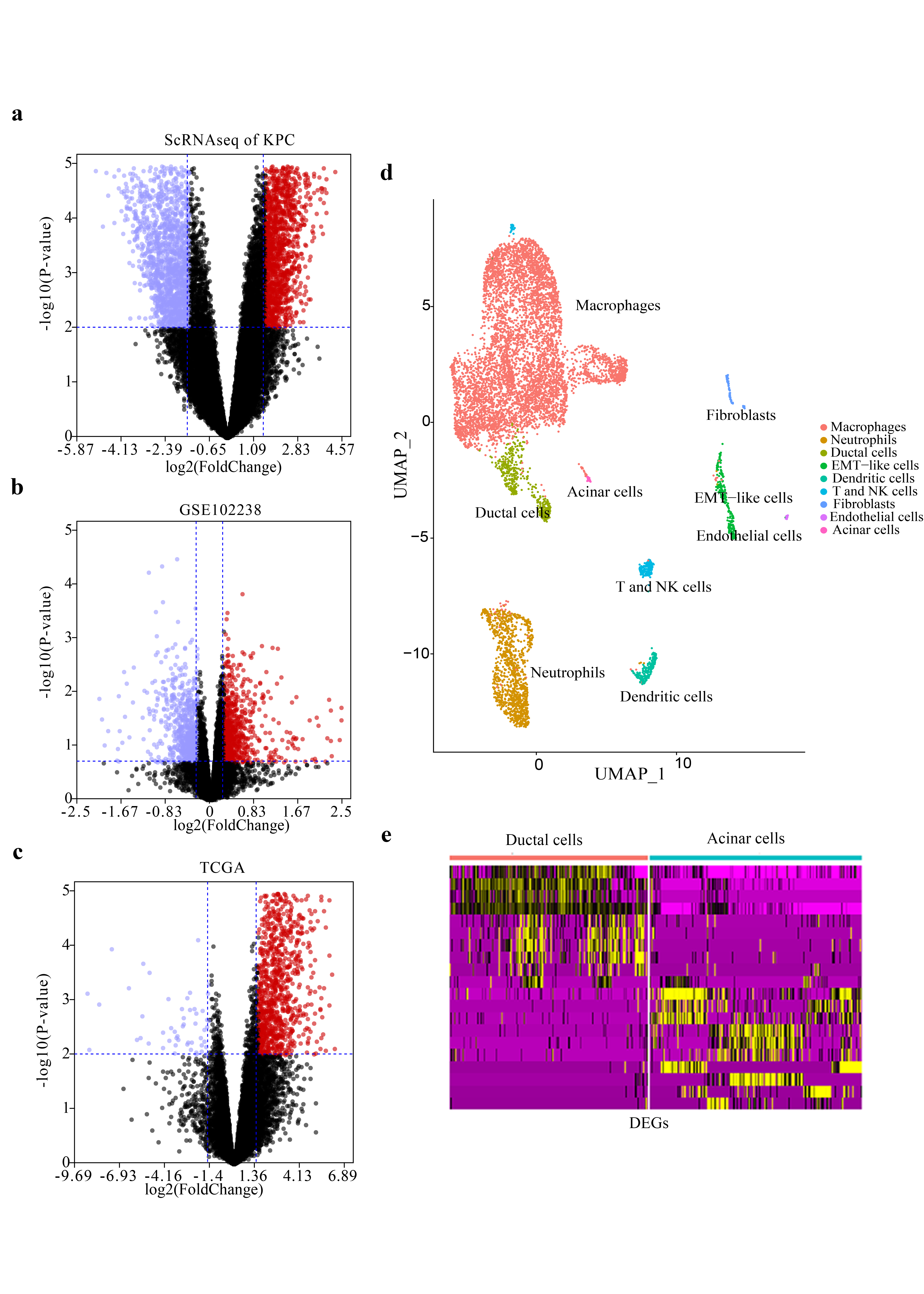

Supplement: Supplementary file 9 [file Image2.TIF]

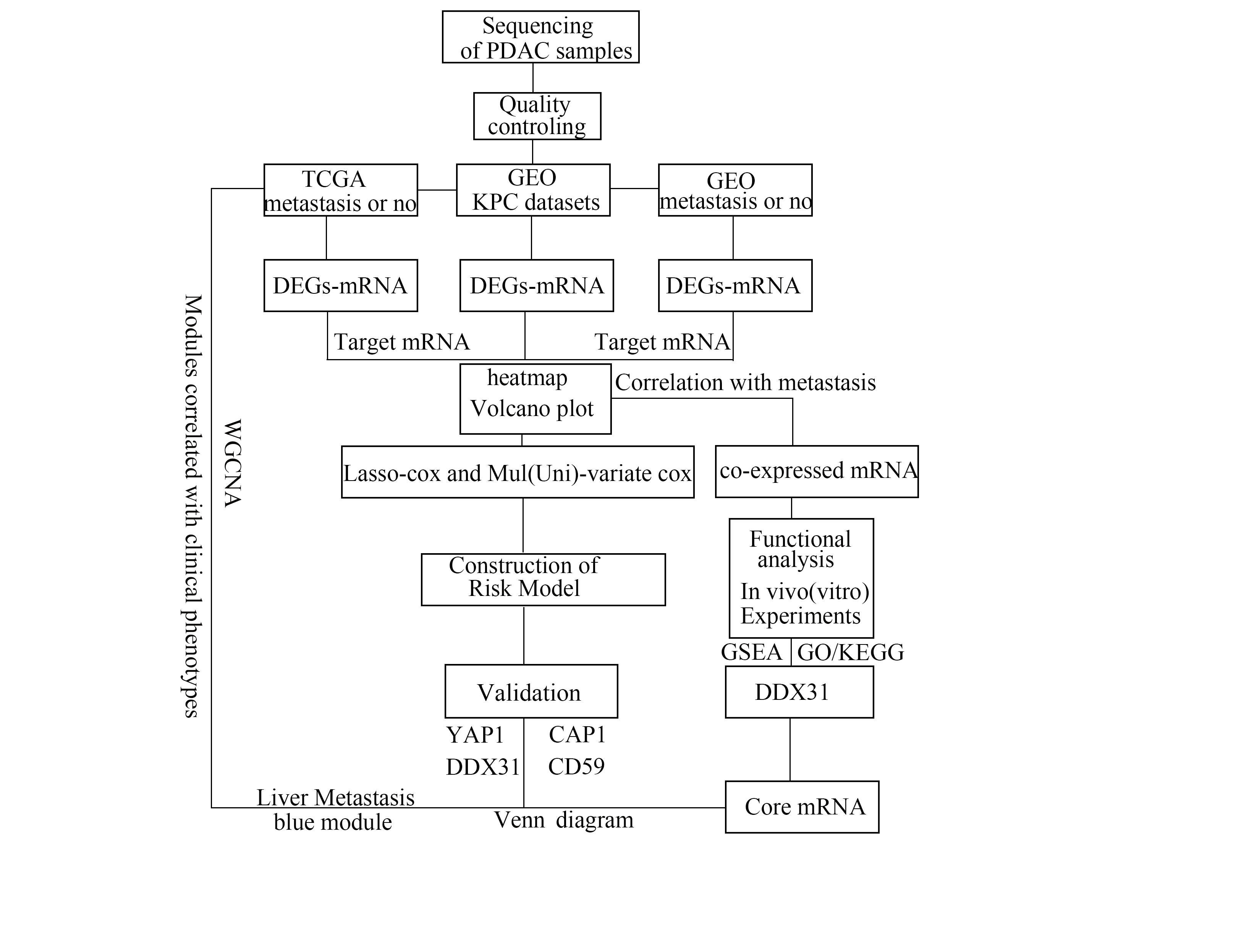

Supplement: Supplementary file 11 [file Image1.TIF]

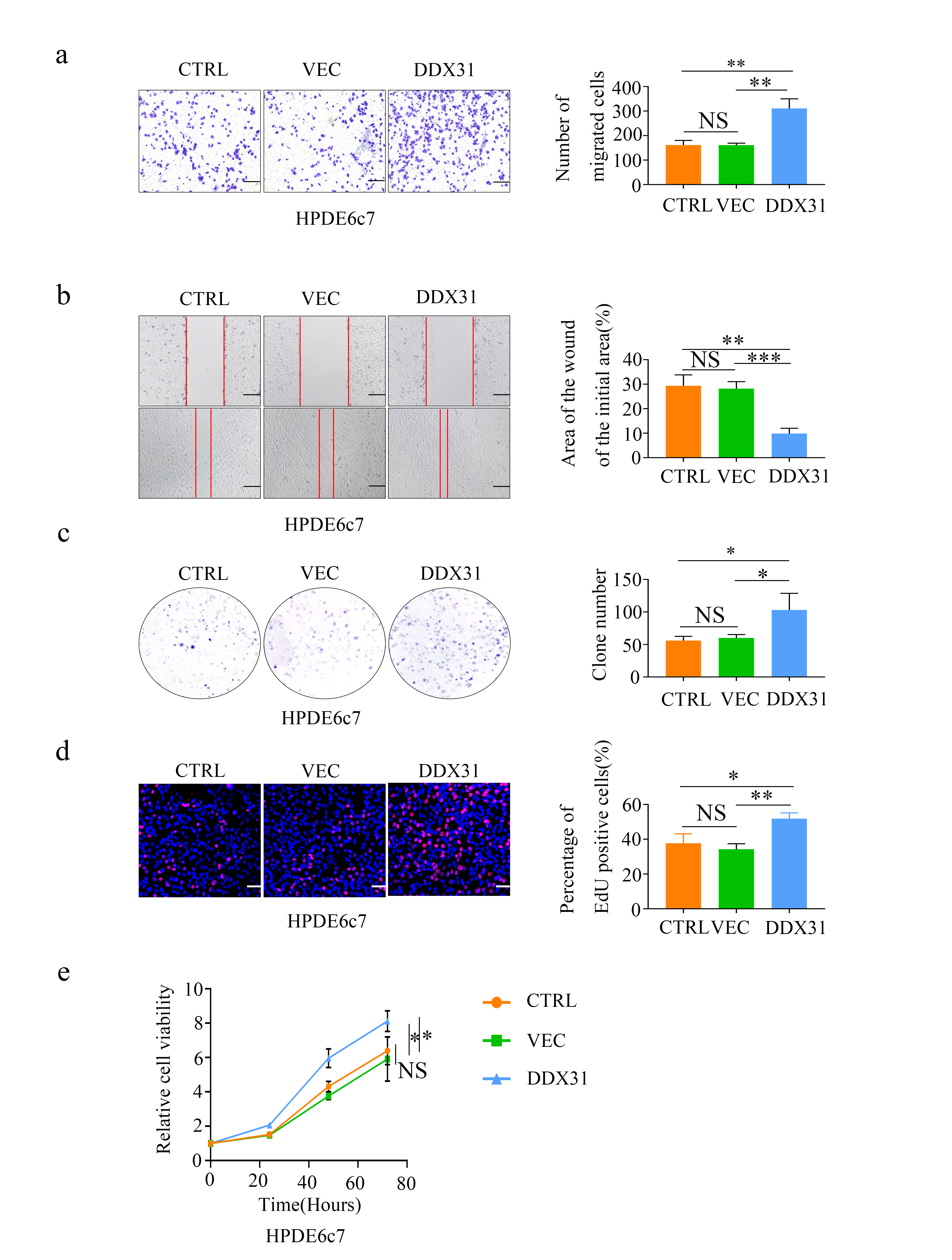

Supplement: Supplementary file 12 [file Image7.TIF]

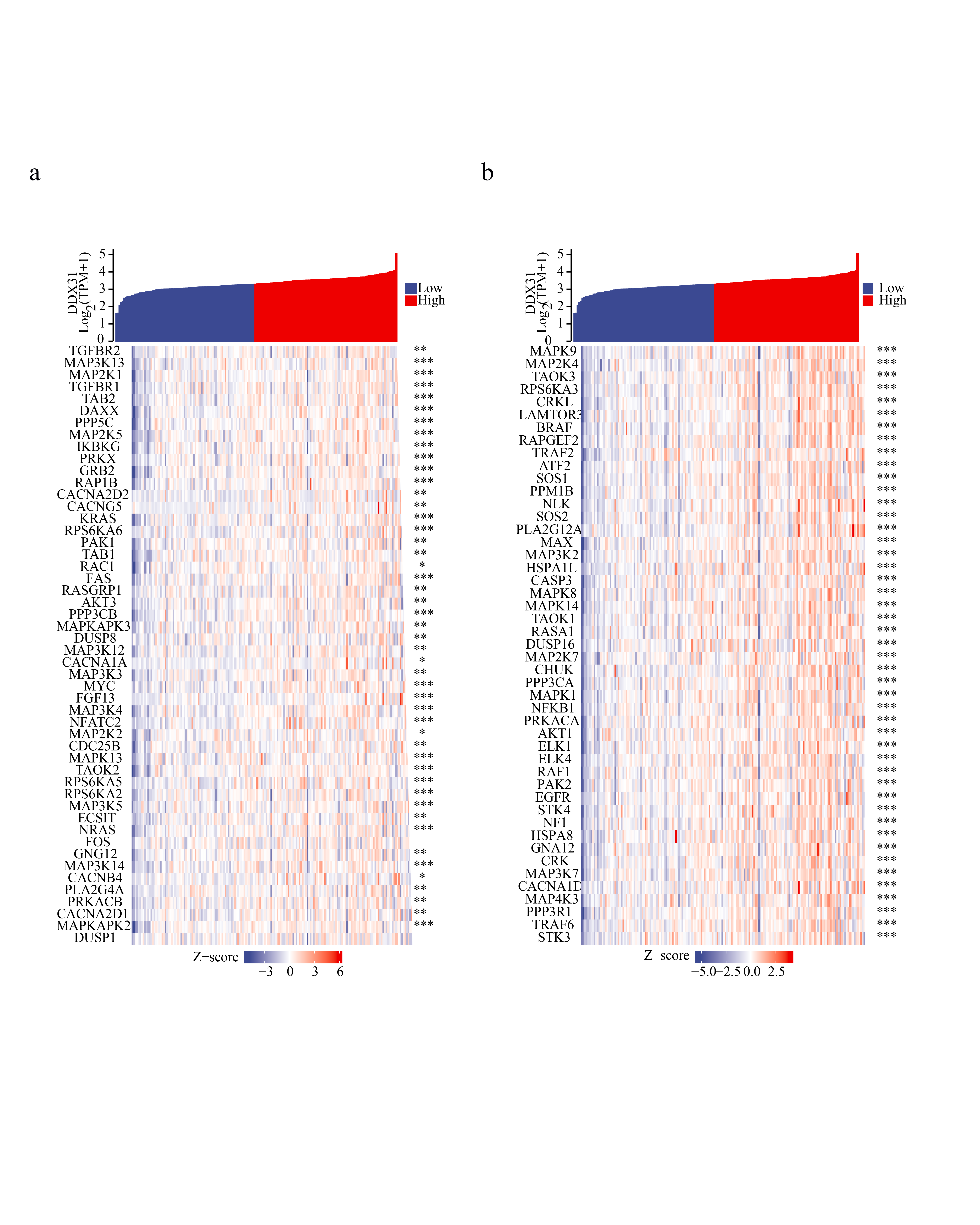

Supplement: Supplementary file 17 [file Image8.TIF]

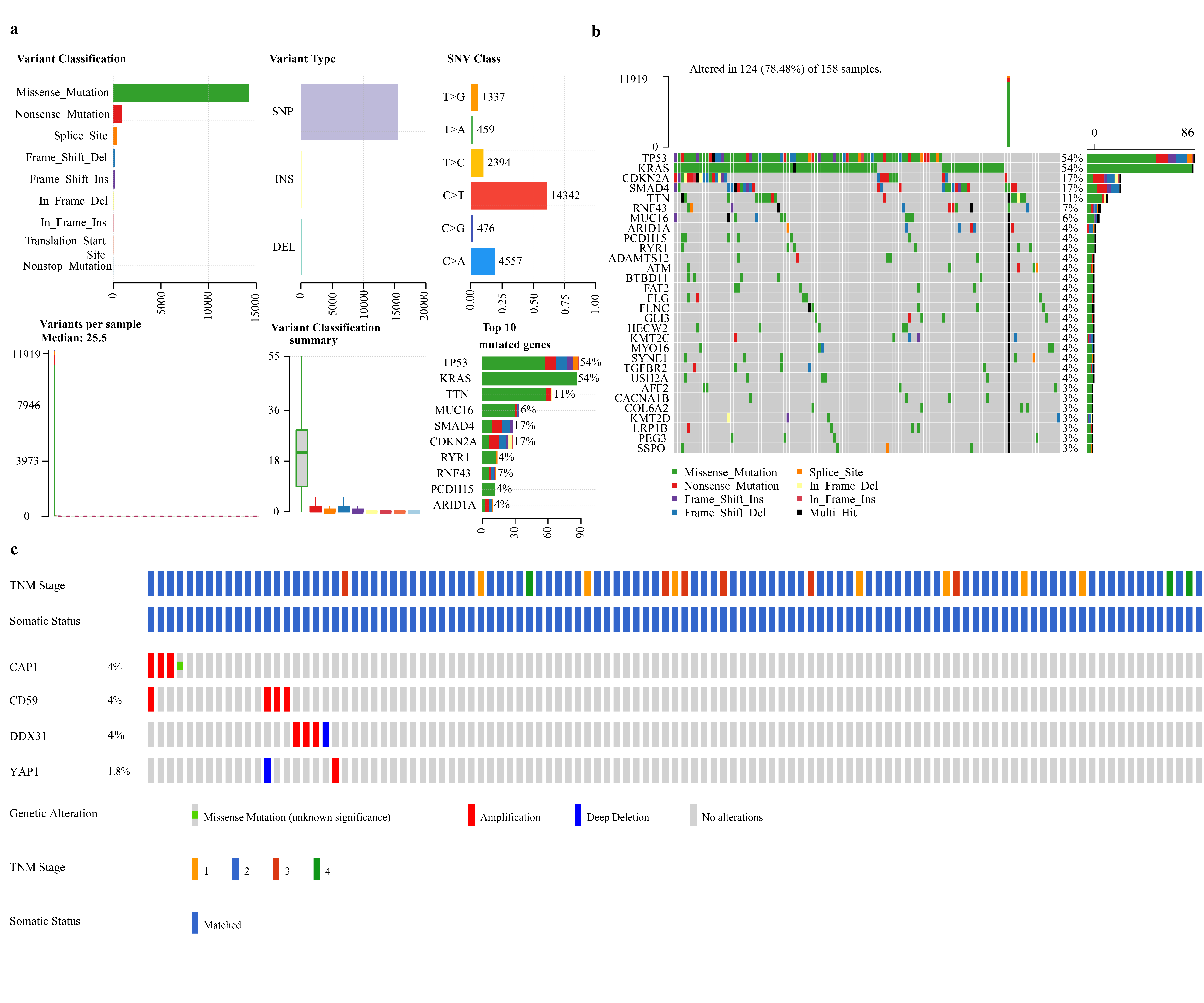

Supplement: Supplementary file 18 [file Image5.TIF]
