# Supplementary material for: Identification of DDX31 as a Potential Oncogene of Invasive Metastasis and Proliferation in PDAC
Source: Front Cell Dev Biol. 2022 Feb 14;10:762372. doi: 10.3389/fcell.2022.762372 (PMC8883474; doi:10.3389/fcell.2022.762372)
Supplement: Supplementary file 14 [file DataSheet1.PDF]

1. Single cell RNA sequencing used in this research**(GSE129455)**

Series GSE129455	Query DataSets for GSE129455
Status	Public on Jun 19, 2019
Title	Cross-species single-cell analysis of pancreatic ductal adenocarcinoma reveals cancer-associated fibroblasts expressing MHC class II
Organism	Mus musculus
Experiment type	Expression profiling by high throughput sequencing
Summary	This study used 10X Genomics, single-cell RNA-sequencing to examine the cell types present in the KrasLSL-G12D; Trp53LSL-R172H; Pdx1-Cre (KPC) mouse model for pancreatic ductal adenocarcinoma. The study analyzed tumors from 4 different mice. For each tumor, we performed flow sorting to isolate all viable cells, and to isolate a fibroblast-enriched population of cells for single-cell RNA-seq to determine the transcriptomes of individual cells in KPC pancreatic ductal adenocarcinoma tumors.
Overall design	Single-cell RNA-sequencing analysis (10X Genomics and Illumina sequencing) of the viable and fibroblast-enriched cell populations taken from 4 replicate mice of the KPC mouse model of pancreatic ductal adenocarcinoma
Contributor(s)	Tuveson DA, Baker LA
Citation(s)	Elyada E, Bolisetty M, Laise P, Flynn WF et al. Cross-Species Single-Cell Analysis of Pancreatic Ductal Adenocarcinoma Reveals Antigen-Presenting Cancer-Associated Fibroblasts. *Cancer Discov* 2019 Aug;9(8):1102-1123. PMID: 31197017

Platforms (1)	GPL21103	Illumina HiSeq 4000 (Mus musculus)
Samples (8) Less...	GSM3713177	KPC1_Viable
	GSM3713178	KPC2_Viable
	GSM3713179	KPC1_FibroblastEnriched
	GSM3713180	KPC2_FibroblastEnriched
	GSM3713181	KPC3_Viable
	GSM3713182	KPC4_Viable
	GSM3713183	KPC3_FibroblastEnriched
	GSM3713184	KPC4_FibroblastEnriched

Relations

BioProject	PRJNA531464
SRA	SRP191615

Download family	Format
SOFT formatted family file(s)	SOFT
MINiML formatted family file(s)	MINiML
Series Matrix File(s)	TXT

Supplementary file	Size	Download	File type/resource
GSE129455_All_Viable_expression.csv.gz	192.1 Mb	(ftp)(http)	CSV
GSE129455_Fibroblast-enriched_expression.csv.gz	191.8 Mb	(ftp)(http)	CSV

2.160 PDAC samples were obtained in the **TCGA** database

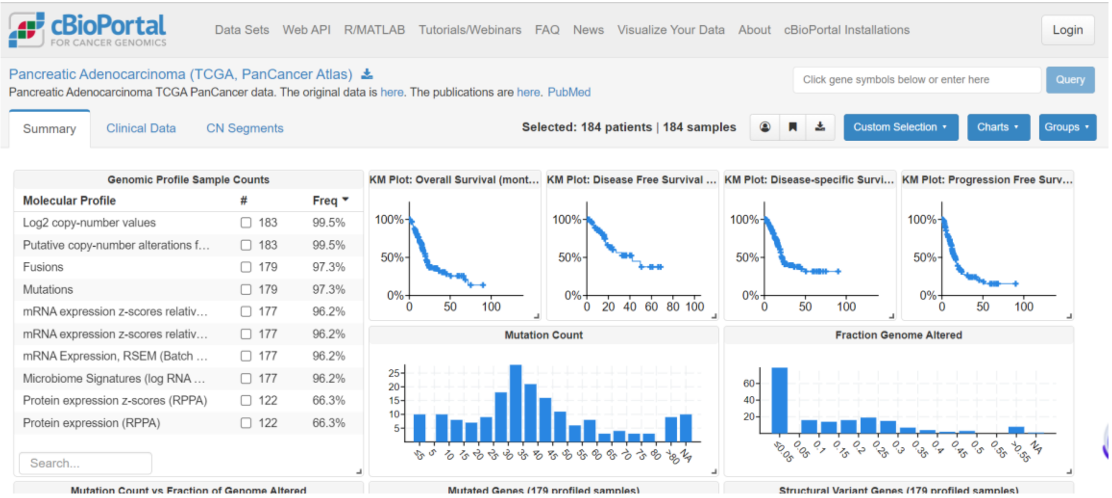

Tags	TCGA-IB-/	TCGA-HZ-	TCGA-2J-/	TCGA-LB-.	TCGA-HZ-	TCGA-S4-	TCGA-3A-	TCGA-HZ-	TCGA-3A-	TCGA-HV-	TCGA-Q3-	TCGA-FB-.	TCGA-LB-.	TCGA-IB-/	TCGA-HV-	TCGA-FB-.	TCGA-I
RAB4B	2.384631	5.722387	1.561563	2.025077	3.284771	3.749104	5.534028	4.274886	10.33748	3.741115	8.001372	4.995055	6.761126	4.979701	10.55151	3.364132	2.2064
C12orf5	1.055911	5.474452	1.236821	1.961533	4.295307	4.729819	2.500603	3.315708	3.916511	2.396826	3.711136	2.643867	1.323633	4.406253	0.950496	3.635824	4.0743
RNF44	8.300917	12.01996	1.498508	4.916926	10.19815	13.18598	10.50974	11.10975	7.279862	8.667191	20.34316	7.933696	10.30182	9.302457	1.447368	10.16018	9.4739
DNAH3	0.040443	0.420755	0.016774	0.078275	0.089607	0.284105	0.165348	0.158229	0.08445	0.059549	0.03267	0.008787	0.599303	0.036591	0.110337	0.214583	0.0301
RPL23A	113.0245	218.2678	202.5684	102.0235	106.8148	191.5151	114.5564	125.7515	145.4592	104.052	289.5944	139.3674	148.5857	120.1012	824.772	177.972	173.46
ARL8B	12.76509	13.89095	5.57864	16.14304	21.70558	29.11267	16.91677	28.0296	35.42008	23.28441	20.63123	24.09256	15.91652	26.27813	15.73682	20.24872	28.289
CALB2	0.955551	10.68795	0.486248	2.13597	1.605181	15.59823	40.6234	2.201327	0.180164	33.25804	15.02766	29.60998	0.590537	6.940585	0.448731	3.495375	0.8378
MFSD3	6.168452	14.58817	9.731762	8.842488	11.15988	12.39208	9.313654	8.629522	28.89888	9.197933	7.49449	7.65794	12.78219	7.406785	15.8455	11.94163	4.2981
PIGV	3.63959	3.288075	2.292681	3.086721	3.948167	2.083139	4.066593	4.400408	4.866928	5.531612	3.560241	3.070631	8.111076	3.710729	2.2415	4.928252	4.6336
ZNF708	1.254467	1.14836	0.253161	0.638454	1.288785	0.876231	0.666814	3.489626	2.581955	1.477964	1.824386	1.304992	1.247759	1.124977	0.339228	1.02522	1.4749
MYADML2	0.008488	0.096031	0	0.070953	0.042774	0.018795	0.032203	0.027647	0.007343	0.016108	0.009038	0.010696	0.470084	0.024745	0	0.034596	
PHEX	0.035948	0.061624	0.007861	0.066346	0.065877	0.047037	0.077494	0.157895	0.192243	0.037212	0.160073	0.074128	0.378747	0.101625	0.019153	0.119884	0.1097
MOGAT2	0.241947	0.766725	0.127316	0.108351	0.107959	0.21207	0.052592	2.462738	0.004361	1.047471	0.230795	0.069872	2.093744	0.146949	0.051697	0.118136	0.8225
PFN2	8.648389	4.117037	3.785887	8.082815	8.244577	7.057263	16.05539	31.35024	61.45304	13.96705	6.73565	19.50412	40.63668	12.07018	5.704759	12.7	23.680
RMND5A	6.521948	9.143234	1.917775	5.690411	6.192623	5.679677	7.55895	13.74118	20.41164	6.095554	5.795457	4.242698	13.2461	4.997018	4.043328	5.517938	9.3343
RAD23A	22.98409	44.94689	12.98215	24.7113	37.22166	51.76275	31.49724	29.84311	37.305	32.35864	36.23038	28.72774	35.10631	33.78469	18.38547	42.97015	35.051
OR2D2	0	0	0	0	0	0	0	0	0.032519	0	0.020014	0	0	0	0	0	

3.**GSE102238** datasets downloaded from GEO database.

Scope: Self Format: HTML Amount: Quick GEO accession: GSE102238 GO

Series GSE102238 Query DataSets for GSE102238

Status	Public on Aug 04, 2017
Title	Gene expression signatures associated with Perineural Invasion in pancreatic ductal adenocarcinoma
Organism	Homo sapiens
Experiment type	Expression profiling by array
Summary	Perineural invasion (PNI) is a prominent characteristic of pancreatic ductal adenocarcinoma (PDAC) and indicates poor prognosis. The invasion of the surrounding nerves by pancreatic cancer cells not only provides route for metastasis but also contributes to neural remodeling and changes in the neuronal milieu that can profoundly influenced the microenvironment of pancreatic cancer. To investigate the downstream molecules associated with PNI, the experiment analyzed mRNA expression of 50 pairs of pancreatic ductal adenocarcinoma tissue and paired adjacent non-tumor tissue, among which 28 pairs of cases diagnosed with PNI by experienced pathologist. Results provide new insight into molecular basis for the influence of PNI on the microenvironment of pancreatic cancer.
Overall design	The aim of our work was to characterize downstream molecules influenced by the perineural invasion of pancreatic cancer. For this purpose, we generated and compared expression profiles of PDAC tissue from patients diagnosed with PNI (n=28) and PDAC tissue from patients without PNI (n=22).

4.**GSE62452** datasets downloaded from GEO database.

Scope: Self | Format: HTML | Amount: Quick | GEO accession: GSE62452 | GO

**Series GSE62452**	Query DataSets for GSE62452
Status	Public on Jul 01, 2016
Title	Microarray gene-expression profiles of 69 pancreatic tumors and 61 adjacent non-tumor tissue from patients with pancreatic ductal adenocarcinoma
Organism	Homo sapiens
Experiment type	Expression profiling by array
Summary	In order to identify biologically relevant tumor markers , we analyzed gene expression profiling of tumor and adjacent non-tumor tissues from PDAC cases. We compared the microarray gene-expression profiles of MIF-high and MIF low expressing tumors as detrmined by qRT-PCR. Affymetrix gene-expression analysis was done in two sets. Affymetrix data from sample number 1-90 were earlier submited by us as GEO accession#: GSE28735. The batch effect between the two sets of data was removed using Partek Genomic Suite and this normalized data was submitted to GEO in this submission. All the analysis was performed using the merged data set.
Overall design	We compared gene expression profile of 69 pancreatic tumor and adjacent non-tumor tissues using Affymetrix GeneChip Human Gene 1.0 ST arrays to identify microRNA targets associated with MIF signaling. Using Partek, we peformed ANOVA and Cox-regression analysis to identify differentially expressed genes that were also associated with survival. The list of genes was then subjected to pathway and biomarker analyses using Ingenuity Pathways Analysis (IPA).
