# Supplementary material for: Identification of DDX31 as a Potential Oncogene of Invasive Metastasis and Proliferation in PDAC
Source: Front Cell Dev Biol. 2022 Feb 14;10:762372. doi: 10.3389/fcell.2022.762372 (PMC8883474; doi:10.3389/fcell.2022.762372)
Supplement: Supplementary file 20 [file Table10.DOCX]

Supplementary table1 Differentially expressed genes among muti-central databases

| ID | Gene | | | | |
| --- | --- | --- | --- | --- | --- |
| DEGs | TUBA1A | CNPY4 | VAT1 | CBX1 | CDH2 |
|  | SMAD7 | SLC39A6 | DCLK2 | BEND6 | MAP1A |
|  | UBE2E2 | ARHGEF17 | ATP6AP2 | RAB2B | WASF1 |
|  | PHLDB2 | AXIN2 | RBFOX2 | BICD2 | VIM |
|  | MXRA7 | VASH1 | KATNAL1 | ID1 | FTO |
|  | ADAMTS2 | BIRC5 | SOX2 | ITGA5 | NDST1 |
|  | TMEM200 | MEIS3 | TCEAL4 | MAD2L1 | EHD3 |
|  | CERCAM | MSN | PRKACA | MARCKS | KIF3C |
|  | LAMB1 | GLI2 | ANXA6 | TIMP2 | FADS1 |
|  | SGTB | BBS9 | YWHAQ | ELOVL5 | FAM127C |
|  | OAZ2 | FAP | NREP | EFHA4 | DDX31 |
|  | ASAP2 | ERRFL1 | MSLN | SAPCD2 | CAP1 |
|  | BAIAP2 | USP43 | PODH1 | SNAI1 | CD59 |
|  | ANXA8 | DUSP4 | PLEK2 | SFN | WFDC2 |
|  | DNAJB5 | TMX3 | ADAM19 | WNT3B | YAP1 |
|  | SCGB2A2 | LRRC53 | BPIFB2 | ACTL6B | CALY |
|  | GAST | TMEM179 | RUNDC3A | VWA5B2 | PCSK1N |
|  | MYC | BPIFB4 | MMD2 | DEFA5 | KRT9 |
|  | TCP11X2 | DLX3 | USP26 | LY6D | MAGEC1 |
|  | KRT13 | HOXC12 | UPK3B | REG3G | PRB1 |
|  | SPINK7 | WIF1 | KLK5 | FAM83A | GFY |
|  | KRT74 | SPRR1B | CYP4F22 | REG3A |  |
